# Clinical Note–Extracted Psychosocial Factors for Predicting Suicide Attempt Among ED Patients With Suicidal Ideation

**DOI:** 10.1001/jamanetworkopen.2026.0589

**Published:** 2026-03-04

**Authors:** Hyunjoon Lee, Ketan Jadhav, Michael Ripperger, Peyton L. Coleman, Theodore J. Morley, Samuel A. Palmer, Lide Han, Qingxia Chen, Cosmin A. Bejan, Douglas M. Ruderfer, Colin G. Walsh

**Affiliations:** 1 Department of Biomedical Informatics, Vanderbilt University Medical Center, Nashville, Tennessee; 2 Center for Digital Genomic Medicine, Department of Medicine, Vanderbilt University Medical Center, Nashville, Tennessee; 3 Vanderbilt Genetics Institute, Vanderbilt University Medical Center, Nashville, Tennessee; 4 Department of Biostatistics, Vanderbilt University Medical Center, Nashville, Tennessee; 5 Department of Psychiatry and Behavioral Sciences, Vanderbilt University Medical Center, Nashville, Tennessee; 6 Department of Medicine, Vanderbilt University Medical Center, Nashville, Tennessee

## Abstract

**Importance:**

The Joint Commission recommends universal suicide screening in emergency departments (EDs), which emphasizes the need to identify at-risk individuals. Existing suicide risk prediction models rely primarily on clinical data and demonstrate limited performance. The potential of incorporating psychosocial information to enhance predictive performance remains understudied.

**Objective:**

To evaluate whether augmenting clinical data–based risk scores with psychosocial factors improves the prediction of suicide attempt (SA).

**Design, Setting, and Participants:**

This retrospective prognostic study based on electronic health record data included 4661 ED patients discharged after presentation for suicidal ideation (SI) from middle Tennessee hospitals between June 1, 2018, and February 27, 2024.

**Main Outcomes and Measures:**

The primary outcome was SA within 90 days of ED admission and time-to-event in days. Clinical data–based Vanderbilt Suicide Attempt and Ideation Likelihood (VSAIL) score and 6 psychosocial factors (homelessness, financial insecurity, chronic stress, social isolation, loneliness, and adverse childhood experiences) derived from clinical notes were integrated using a Cox proportional hazards regression model. Performance metrics included area under the receiver operating curve (AUROC), area under the precision-recall curve (AUPRC), positive predictive value (PPV), negative predictive value, sensitivity, and specificity. Performance was evaluated for models trained on (1) VSAIL, (2) psychosocial factors, and (3) VSAIL plus psychosocial factors.

**Results:**

This study included 3382 Vanderbilt University Hospital (VUH) (mean [SD] age, 26.1 [15.6] years; 1751 males [51.8%]) and 1279 Regional Health Systems (RHS) (mean [SD] age, 34.5 [18.0] years; 715 males [55.9%]) ED visits for SI. Within 90 days, SAs were reported in 160 (4.7%) VUH and 34 (2.7%) RHS ED visits for SI. Compared with VSAIL alone, VSAIL plus psychosocial factors was associated with significantly increased median AUROC (VUH: 0.645 [IQR, 0.645-0.645] vs 0.734 [IQR, 0.719-0.747]; *P* < .001; RHS: 0.547 [IQR, 0.547-0.547] vs 0.680 [IQR, 0.672-0.687]; *P* < .001), AUPRC (VUH: 0.083 [IQR, 0.083-0.083] vs 0.122 [IQR, 0.111-0.137]; *P* < .001; RHS: 0.029 [IQR, 0.029-0.029] vs 0.054 [IQR, 0.052-0.058]; *P* < .001), and PPV (VUH: 0.093 [IQR, 0.082-0.094] vs 0.143 [IQR, 0.123-0.161]; *P* < .001; RHS: 0.042 [IQR, 0.040-0.043] vs 0.112 [IQR, 0.096-0.129]; *P* < .001) while maintaining specificities above 0.90. Chronic stress emerged as the strongest predictor of SA (β = 0.643 [95% CI, 0.427-0.859]; *P* < .001).

**Conclusions and Relevance:**

In this prognostic study of patients discharged from the ED after presentation for SI, augmenting a clinical data–based suicide risk prediction model with clinical note–extracted psychosocial factors was associated with significantly higher predictive performance. These findings suggest that psychosocial factors can enhance risk stratification and support targeted interventions, such as therapies addressing chronic stress.

## Key Points

**Question:**

Is the addition of psychosocial factors to a clinical data–based suicide risk prediction model associated with better performance in predicting suicide attempts among patients presenting to the emergency department (ED) for suicidal ideation?

**Findings:**

In this electronic health record–based prognostic study of 4661 patients discharged from the ED after presentation for suicidal ideation, incorporating psychosocial factors was associated with significantly higher performance in predicting suicide attempt, with chronic stress as the strongest predictor.

**Meaning:**

This study suggests that, for ED patients with suicidal ideation, identifying and using psychosocial factors may be key to accurate risk stratification and may help guide targeted interventions such as therapies addressing chronic stress.

## Teaser

This prognostic study evaluates whether augmenting clinical database risk scores with psychosocial factors was associated with improved prediction of suicide attempt among emergency department patients.

## Introduction

Suicide represents a critical public health crisis in the US, ranking among the top 5 causes of death for individuals aged 10 to 54 years.^1^ Despite more than 5 decades of research on risk factors and prevention strategies,^2,3^ national suicide rates increased by 37% between 2000 and 2022.^4^

Over half of individuals who die by suicide have a health care encounter in the month prior, underscoring these visits as critical opportunities to identify and intervene in suicide risk.^5,6^ The Joint Commission recommends screening patients at high risk using validated tools such as the 9-item Patient Health Questionnaire (PHQ-9).^7^ Following The Joint Commission guidelines, many health care systems implement universal screening for patients presenting to emergency departments (EDs), but screening tools have shown limited ability to predict suicide attempt (SA). For instance, the positive predictive value (PPV) of a patient reporting daily suicidal ideation (SI) on the PHQ-9 for predicting SA within 1 year is approximately 0.04.^8^

Multiple efforts have sought to bolster risk prognostication by using electronic health record (EHR) data and statistical models to identify individuals at high risk of SA. However, these approaches have struggled to achieve clinically actionable performance.^9,10^ A key challenge is significant class imbalance; while suicide ranks among the leading causes of death,^1^ it is a rare event at health system scale. Therefore, EHR-based models often produce PPVs of less than 0.01.^11^ Nevertheless, these models may hold particular relevance in EDs, where individuals at elevated suicide risk are overrepresented.^10,12,13^ More than 8% of ED visits involve psychiatric or substance use–related conditions, and more than 1% (1.5 million) involve SI or SA.^14,15^ In ED settings, EHR-based models show higher PPVs; for example, Sheu et al^16^ reported a PPV of 0.09 for predicting SA (prevalence, 1.6%) within 180 days of psychiatric ED visits.

Incorporating psychosocial factors holds promise for improving EHR-based suicide risk prediction models. Substantial evidence indicates that psychosocial factors such as adverse childhood experiences (ACEs) and homelessness are strong predictors of SA.^17,18^ However, studies show *International Statistical Classification of Diseases and Related Health Problems, Tenth Revision* (*ICD-10*) Z codes for psychosocial factors are heavily underused.^19,20,21,22^ In contrast, unstructured clinical notes provide a more comprehensive repository of psychosocial factor–related information.^18^ To address these gaps, prior studies applied natural language processing (NLP) to clinical notes to enhance suicide risk prediction, extracting psychosocial factors such as marital status, family support, and social stress using Unified Medical Language System Concept Unique Identifiers (CUIs).^23,24^ However, CUI-based extraction lacks contextual nuances. Advanced NLP techniques, such as those using vector embeddings, may enable more precise and context-sensitive, yet scalable, extraction of psychosocial factors.

This study evaluated whether augmenting risk scores from a clinical data–based suicide risk prediction model with clinical note–extracted psychosocial factors was associated with improved prediction of SA. We focused on patients discharged from the ED after presentation for SI because the existing universal suicide screening in EDs provides a standardized workflow conducive to the integration of risk prediction models. We integrated risk scores from the Vanderbilt Suicide Attempt and Ideation Likelihood (VSAIL) model and psychosocial factors extracted from clinical notes using an NLP algorithm developed, validated, and deployed at Vanderbilt University Medical Center (VUMC).^25,26,27,28,29^ Our study had 2 objectives: (1) to assess whether augmenting VSAIL scores with clinical note–extracted psychosocial factors was associated with higher predictive performance and (2) to identify predictors associated most significantly with suicide risk.

## Methods

This study, approved by the institutional review board at VUMC with a waiver of consent due to the use of deidentified data for which reconsent was not feasible, used VUMC Synthetic Derivative, a deidentified EHR data repository of more than 3.9 million patients spanning 20 years.^30^ This study followed the Transparent Reporting of a Multivariable Prediction Model for Individual Prognosis or Diagnosis (TRIPOD) reporting guideline.^31^

### Study Settings

As the primary sites for training the models, we included Vanderbilt and Monroe Carell Jr Children’s Hospital EDs (Vanderbilt University Hospitals [VUH]), both major urban tertiary care centers located in Nashville, Tennessee. To assess generalizability, we used the Regional Health System (RHS) in middle Tennessee, including Vanderbilt Bedford Hospital, Tullahoma-Harton Hospital, and Wilson County Hospital as external validation sites. These facilities are separate rural hospitals with distinct sets of health care professionals that serve patient populations that are geographically and demographically different from those at VUH.

### Study Population

We included all ED visits discharged after presentation for SI from June 1, 2018, to February 27, 2024, at VUH and RHS, after implementation of universal suicide screening. SI was identified using *International Classification of Diseases, Ninth Revision* (*ICD-9*) code V62.84, *ICD-10* code R45.851, and clinical notes using a validated NLP algorithm.^26,28^ For patients with multiple ED visits for SI, we randomly selected 1 visit to avoid overrepresentation of patients with frequent ED visits for SI. Sensitivity analysis from prior research demonstrated similar model performance when sampling 1 visit per patient vs all visits.^16^ ED visits for SI with concurrent SA were excluded to focus on predicting SA among patients experiencing SI, most of whom do not make an attempt. Visits with missing or abnormal body mass index (BMI; calculated as weight in kilograms divided by height in meters squared) (<10 or >70) or Area Deprivation Index that remained unresolved after imputation using previsit data were excluded. Last, visits overlapping with the VSAIL training dataset were excluded (eFigure 1 in Supplement 1).

### NLP Algorithm

The process to generate each NLP algorithm was developed using a semiautomated approach to extract SI, SA, and psychosocial factors from clinical notes.^25,26,27,28^ Algorithmic development began with seed query terms (eg, *suicide attempt* and *homelessness*) and expanded the query terms using contextual embeddings and statistical lexical association. Next, the system ranked text expressions from clinical notes based on their alignment with the expanded query terms. Finally, experts manually reviewed the top 50–ranked text expressions and repeated several iterations of this process to refine the query terms.

Once the query terms were finalized, they were input into a search engine applied to all clinical notes in the EHR, initiating a fully automated score generation process. Clinical notes and query terms were encoded as multidimensional term frequency-inverse document frequency–weighted vectors, and their similarity was assessed using the cosine similarity metric. The algorithm applied negation filtering to reduce false positives and assigned a relevance score to each document that contained at least 1 positive assertion of a query term. Higher scores indicate stronger evidence of the psychosocial factor’s presence, not severity. Validation studies demonstrated a 90-fold increase in psychosocial factor detection (PPVs >0.9) compared with diagnostic codes.^25^

### Outcomes

The outcome was SA within 90 days of ED admission for SI, identified using *ICD-9* and *ICD-10* codes (eTable 1 in Supplement 1) and clinical notes analyzed with a validated NLP algorithm.^26,28^ SA date was determined by *ICD-9* and *ICD-10* code entry date and the clinical note indicating strong evidence of SA. We defined 2 outcome variables: (1) a binary SA indicator (SA vs no SA) and (2) time to SA in days (censored at 90 days for nonevents).

### Predictors

#### VSAIL Score

Our clinical data–based predictor was VSAIL score, a composite SA risk estimate generated by a random forest model trained on structured EHR data at VUMC.^29^ VSAIL was trained using data from an adult patient population of more than 600 000 individuals and incorporated more than 800 predictors, including diagnoses, medications, procedures, demographics (eg, age, sex, race and ethnicity), BMI, and Area Deprivation Index (Census 2000 version) by 3-digit patient zip code (low resolution due to deidentification).^32^ Race and ethnicity (Asian, Black, Hispanic or Latino, White, and other [American Indian or Alaska Native, Native Hawaiian and Other Pacific Islander, and those declining to answer]) were determined by demographic tables in the EHR. We reported race and ethnicity (along with age and sex) to communicate the demographic composition of our study population. Although VSAIL was originally developed to predict SA over a 30-day period, its performance has been validated to be consistent across multiple prediction horizons (eg, 60, 90, and 180 days).^29,33^ We selected a 90-day period to balance a time frame short enough for actionable insights with a sufficient number of SAs for statistical power.

#### Psychosocial Factor Relevance Scores

We derived 6 psychosocial factor relevance scores from clinical notes using a validated NLP algorithm: homelessness, financial insecurity, chronic stress, social isolation, loneliness, and ACEs.^25,27^ Psychosocial factor relevance score serves as a proxy for the potential history or presence of the corresponding psychosocial factor. Patients whose clinical notes did not contain at least 1 positive assertion of a psychosocial factor query term were treated as having no identifiable psychosocial factors. When psychosocial factor was not documented, we assigned a value of zero. To reduce the skew of the exponentially distributed relevance scores and accommodate mathematical validity, we applied a log (x + 1) transformation.

### Statistical Analysis

As defined by Sheu et al,^16^ we adopted a “landmark modeling” framework, which mirrors the data available to health care professionals at the time of prediction and estimates prospective risk from a designated landmark visit. Using the Python package lifelines (Python, version 3.11.9; Python Software Foundation), we trained Cox proportional hazards regression models anchored at the ED admission for SI (landmark visit).^34^

Both VSAIL and psychosocial factor relevance scores were derived using data preceding the ED visit for SI; VSAIL used up to 5 years of prior data, and psychosocial factor scores were derived from all prior clinical notes. We compared the distributions of VSAIL and log (x + 1)–transformed psychosocial factor relevance scores between ED visits for SI with and without subsequent SA (SA group vs no-SA group) using a 2-sample permutation test using the R coin package (R, version 4.4.2; R Project for Statistical Computing).^35^ This nonparametric method was chosen because the distributions were nonnormal.

To simulate the constraints of VSAIL, which is implemented in production and operates in real time,^36^ we avoided retraining and instead incorporated the 6 psychosocial factor relevance scores alongside the VSAIL score as covariates in the Cox proportional hazards regression models. Each predictor was standardized to have a mean (SD) value of 0 (1). To evaluate the association of adding psychosocial factors with the performance yield, we compared performances of 3 configurations: (1) VSAIL, (2) psychosocial factors, and (3) VSAIL plus psychosocial factors.

Performance was assessed using the area under the receiver operating characteristic curve (AUROC) and the area under the precision-recall curve (AUPRC). In addition, for classification based on the top decile of risk, we evaluated PPV, negative predictive value, specificity, and sensitivity. The top decile threshold was chosen based on previous works,^24,37^ which showed that this cutoff effectively captured most SA cases while achieving a specificity of approximately 0.9. Performance metrics were estimated using 250 iterations of stratified bootstrap resampling with replacement, ensuring that the outcome prevalence was preserved across resampling iterations.^38,39^ Specifically, VUH data were split 80:20 into training and testing datasets. During each iteration, a Cox proportional hazards regression model was trained on the resampled training dataset, and the top decile threshold was determined using out-of-bag samples (data left out of the resampled training dataset). Model performance was assessed on the VUH testing dataset (internal validation) and the full RHS dataset (external validation). For each performance metric, we report the median along with IQRs across bootstrap iterations. We compared AUROCs and AUPRCs between (1) VSAIL vs psychosocial factors, (2) VSAIL vs VSAIL plus psychosocial factors, and (3) psychosocial factors vs VSAIL plus psychosocial factors using bootstrapped confidence intervals (CIs) for the median difference and Wilcoxon signed rank test. Predictor importance was assessed using the standardized β coefficients from the Cox proportional hazards regression model trained on full VUH dataset. All *P* values were from 2-sided tests and results were deemed statistically significant at *P* < .05.

## Results

Our study included 3382 VUH ED visits for SI (mean [SD] age, 26.1 [15.6] years; 1751 male [51.8%] and 1631 female [48.2%]; 46 Asian [1.4%], 670 Black [19.8%], 285 Hispanic or Latino [8.4%], 2294 White [67.8%], and 240 of other race [7.1%]) and 1279 RHS ED visits for SI (mean [SD] age, 34.5 [18.0] years; 715 male [55.9%] and 564 female [44.1%]; 4 Asian [0.3%], 116 Black [9.1%], 58 Hispanic or Latino [4.5%], 1031 White [80.6%], and 60 of other race [4.7%]) (Table 1). Within 90 days, SAs were reported in 4.7% of VUH ED visits for SI (160 of 3382; *ICD* code: 141 [81.9%], NLP: 29 [18.1%]) and 2.7% of RHS ED visits for SI (34 of 1279; *ICD* code: 31 [91.2%], NLP: 3 [8.8%]). For VUH ED visits for SI, VSAIL scores (median, 0.057 [IQR, 0.031-0.091] vs 0.035 [IQR, 0.016-0.080]; *P* = .001) and proportions of nonzero chronic stress (65.6% [105 of 160] vs 46.2% [1490 of 3222]; *P* < .001), social isolation (25.6% [41 of 160] vs 12.7% [409 of 3222]; *P* = .002), and ACEs (25.0% [40 of 150] vs 12.6% [407 of 3222]; *P* = .002) differed significantly between the SA and no-SA groups (eFigure 2 in Supplement 1). For RHS ED visits for SI, only the proportion of nonzero ACEs (26.5% [9 of 34] vs 10.3% [128 of 1245]; *P* = .005) differed significantly.

**Table 1.** Baseline Patient Demographic, Clinical, and Psychosocial Factors Characteristic of ED Visits for SI

Characteristic	No. (%)		*P* value^a^
	VUH (n = 3382)	RHS (n = 1279)	
No. of EDs	2	3	NA
Geographic location	Urban	Rural	NA
SI source			
*ICD-9* or *ICD-10* code	3319 (98.1)	1279 (100)	NA
Clinical note	63 (1.9)	0	NA
Age at admission, y			
Mean (SD)	26.1 (15.6)	34.5 (18.0)	<.001
Median (IQR)	18.5 (14.4 to 36.5)	31.4 (19.4 to 46.7)	
Sex			
Female	1631 (48.2)	564 (44.1)	.01
Male	1751 (51.8)	715 (55.9)	
Race^b^			
Asian	46 (1.4)	4 (0.3)	<.001
Black	670 (19.8)	116 (9.1)	
White	2294 (67.8)	1031 (80.6)	
Other	240 (7.1)	60 (4.7)	
Unknown	132 (3.9)	68 (5.3)	
Ethnicity^b^			
Hispanic or Latino	285 (8.4)	58 (4.5)	<.001
Non-Hispanic or non-Latino	1937 (57.3)	493 (38.5)	
Unknown	1160 (34.3)	728 (56.9)	
ADI			
Mean (SD)	27.7 (35.5)	50.1 (17.4)	<.001
Median (IQR)	39.9 (−13.6 to 64.2)	53.4 (39.9 to 64.2)	
BMI			
Mean (SD)	25.3 (7.4)	27.9 (8.1)	<.001
Median (IQR)	23.8 (20.2 to 28.9)	26.5 (22.1 to 31.7)	
≥1 Positive assertion in clinical notes			
Homelessness	1012 (29.9)	317 (24.8)	<.001
Financial insecurity	520 (15.4)	224 (17.5)	.08
Chronic stress	1595 (47.2)	594 (46.4)	.68
Social isolation	450 (13.3)	137 (10.7)	.02
Loneliness	260 (7.7)	75 (5.9)	.04
Adverse childhood experiences	447 (13.2)	137 (10.7)	.02

Abbreviations: ADI, Area Deprivation Index; BMI, body mass index (calculated as weight in kilograms divided by height in meters squared); ED, emergency department; *ICD-9*, *International Classification of Diseases, Ninth Revision*; *ICD-10*, *International Statistical Classification of Diseases and Related Health Problems, Tenth Revision*; NA, not applicable; RHS, Regional Health Systems; SI, suicidal ideation; VUH, Vanderbilt University Hospital.

^a^ We performed the Wilcoxon rank sum test (Mann-Whitney test) to compare age at admission, ADI, and BMI and the Pearson χ^2^ test to compare sex, race and ethnicity, and proportion of ED visits for SI with at least 1 positive assertion in clinical notes.

^b^ Race and ethnicity were determined by demographic tables in the electronic health record. The “Other” group includes American Indian or Alaska Native, Native Hawaiian and Other Pacific Islander, and those declining to answer.

### Performance by Data Modality

**Figure 1.**
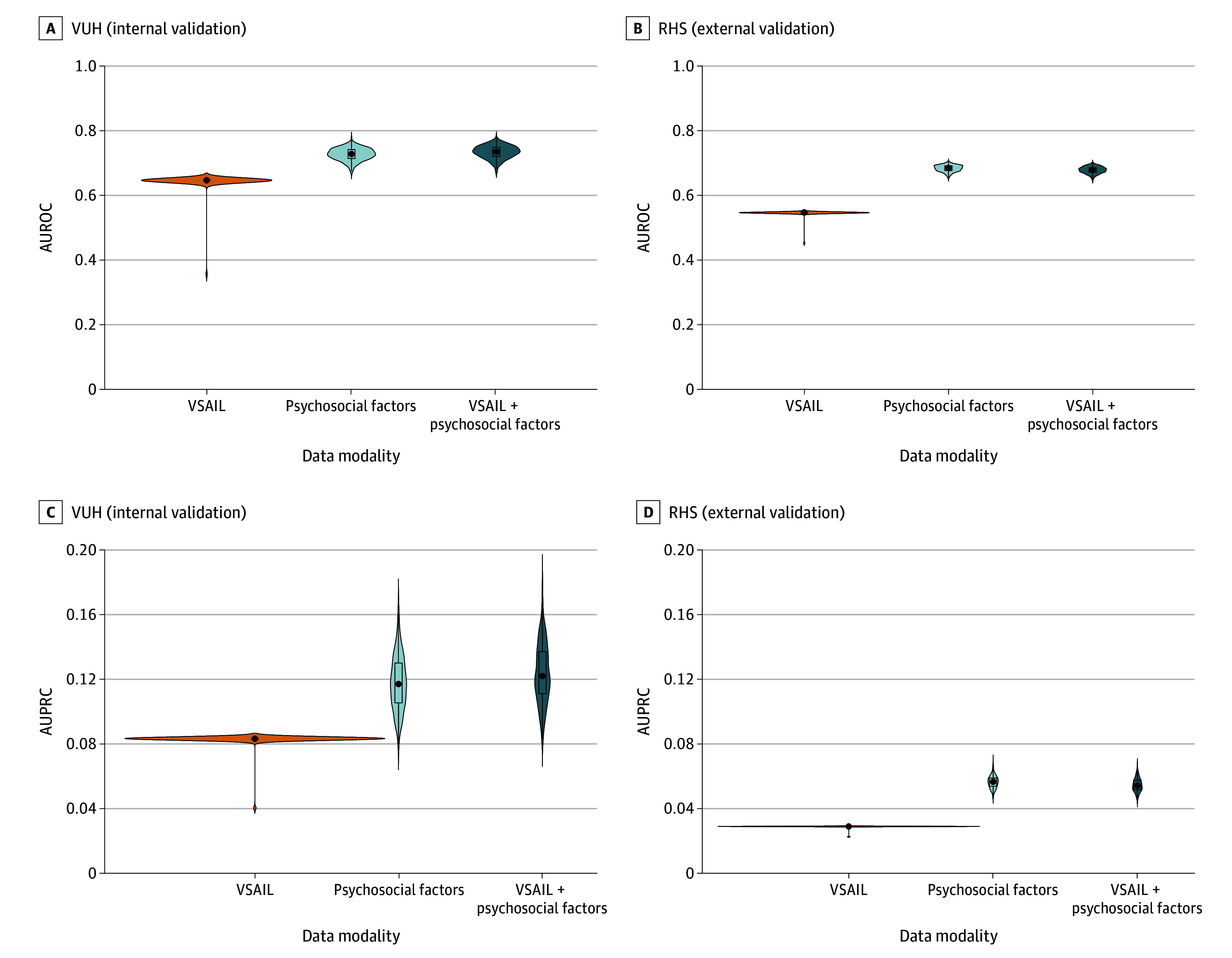
Violin Plots of the Area Under the Receiver Operating Curves (AUROCs) and Area Under the Precision-Recall Curves (AUPRCs) of Bootstrap Resampling for Each Data Modality Configuration The black dots indicate the median AUROC and AUPRC. Models were trained using data from VUH (Vanderbilt University Hospital; Nashville, Tennessee). VUH reflects internal validation performance, and RHS (Regional Health Systems) reflects external validation performance in a separate rural hospital setting serving middle Tennessee (VUH: n = 3382, suicide attempt within 90 days = 160 [4.7%]; RHS: n = 1279, suicide attempt within 90 days = 34 [2.7%]). VSAIL indicates Vanderbilt Suicide Attempt and Ideation Likelihood.

The VSAIL models produced a median AUROC of 0.645 (IQR, 0.645-0.645) and a median AUPRC of 0.083 (IQR, 0.083-0.083) on VUH test data, and a median AUROC of 0.547 (IQR, 0.547-0.547) and a median AUPRC of 0.029 (IQR, 0.029-0.029) on RHS data (Figure 1; eTable 2 in Supplement 1). Compared with VSAIL models, both psychosocial factors and VSAIL plus psychosocial factors models achieved a higher median AUROC (VUH: 0.645 [IQR, 0.645-0.645] vs 0.726 [IQR, 0.713-0.740] and 0.734 [IQR, 0.719-0.747]; RHS: 0.547 [IQR, 0.547-0.547] vs 0.685 [IQR, 0.677-0.692] and 0.680 [IQR, 0.672-0.687]; *P* < .001) and a higher median AUPRC (VUH: 0.083 [IQR, 0.083-0.083] vs 0.117 [IQR, 0.106-0.130] and 0.122 [IQR, 0.111-0.137]; RHS: 0.029 [IQR, 0.029-0.029] vs 0.057 [IQR, 0.054-0.059] and 0.054 [IQR, 0.052-0.058]; *P* < .001). At VUH, the median AUROC increased by 12%, and the median AUPRC increased by 47%; at RHS, the median AUROC increased by 24%, and the median AUPRC increased by 86%. Compared with psychosocial factors models, VSAIL plus psychosocial factors models demonstrated a higher AUROC (0.726 [IQR, 0.713-0.740] vs 0.734 [IQR, 0.719-0.747]; *P* < .001) and a higher AURPC (0.117 [IQR, 0.106-0.130] vs 0.122 [IQR, 0.111-0.137]; *P* < .001) at VUH but a lower AUROC (0.685 [IQR, 0.677-0.692] vs 0.680 [IQR, 0.672-0.687]; *P* < .001) and a lower AUPRC (0.057 [IQR, 0.054-0.059] vs 0.054 [IQR, 0.052-0.058]; *P* < .001) at RHS.

Using the top decile as the threshold, the VSAIL models produced a median PPV of 0.093 (IQR, 0.082-0.094) on VUH test data and 0.042 (IQR, 0.040-0.043) on RHS data (Table 2; eTable 2 in Supplement 1). Compared with VSAIL models, both psychosocial factors and VSAIL plus psychosocial factors models achieved a higher median PPV (VUH: 0.093 [IQR, 0.082-0.094] vs 0.140 [IQR, 0.118-0.161] and 0.143 [IQR, 0.123-0.161]; RHS: 0.042 [IQR, 0.040-0.043] vs 0.110 [IQR, 0.093-0.127] and 0.112 [IQR, 0.096-0.129]; *P* < .001). PPVs of psychosocial factors and VSAIL plus psychosocial factors models were clinically comparable despite statistical significance (VUH: 0.140 [IQR, 0.118-0.161] vs 0.143 [IQR, 0.123-0.161]; bootstrap CI, 0.0002-0.003; *P* = .02; RHS: 0.110 [IQR, 0.093-0.127] vs 0.112 [IQR, 0.096-0.129]; bootstrap CI, 0.001-0.003; *P* = .01). The median specificity exceeded 0.9 across all 3 configurations for both sites.

**Table 2.** Performance Metrics for Predicting Suicide Attempt Within 90 Days^a^

Data modality^b^	Median (IQR)			
	PPV	NPV	Specificity	Sensitivity
**VUH (n = 3382)**				
VSAIL	0.093 (0.082-0.094)	0.957 (0.956-0.957)	0.924 (0.918-0.932)	0.156 (0.156-0.156)
Psychosocial factors	0.140 (0.118-0.161)	0.962 (0.960-0.964)	0.916 (0.909-0.924)	0.281 (0.250-0.313)
VSAIL + psychosocial factors	0.143 (0.123-0.161)	0.963 (0.960-0.964)	0.918 (0.912-0.922)	0.281 (0.250-0.313)
**RHS (n = 1279)**				
VSAIL	0.042 (0.040-0.043)	0.514 (0.511-0.515)	0.907 (0.906-0.910)	0.059 (0.059-0.059)
Psychosocial factors	0.110 (0.093-0.127)	0.493 (0.490-0.495)	0.940 (0.935-0.944)	0.147 (0.118-0.176)
VSAIL + psychosocial factors	0.112 (0.096-0.129)	0.493 (0.491-0.495)	0.940 (0.935-0.945)	0.118 (0.118-0.147)

Abbreviations: NPV, negative predictive value; PPV, positive predictive value; RHS, Regional Health Systems; VSAIL, Vanderbilt Suicide Attempt and Ideation Likelihood; VUH, Vanderbilt University Hospital.

^a^ Performance metrics were based on discrimination at top decile of risk.

^b^ Models were trained using data from VUH (Nashville, Tennessee). VUH reflects internal validation performance, and RHS reflects external validation performance in a separate rural hospital setting serving middle Tennessee.

### Predictor Importance

**Figure 2.**
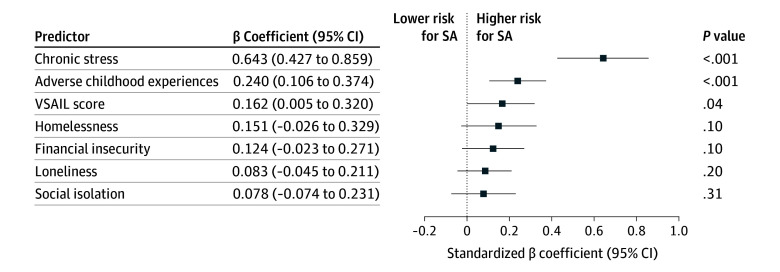
Standardized β Coefficients and 95% CIs for Predictors of 90-Day Suicide Attempt (SA) From a Cox Proportional Hazards Regression Model All predictors were standardized to a mean (SD) value of 0 (1), enabling direct comparison of β coefficients. The Vanderbilt Suicide Attempt and Ideation Likelihood (VSAIL) score represents a composite SA risk estimate, generated by a random forest model trained on structured electronic health record data at Vanderbilt University Medical Center (VUMC). The 6 psychosocial factors were derived as log-transformed cosine similarity metrics (relevance scores), extracted from clinical notes using a vector embedding–based natural language processing algorithm developed, validated, and implemented at VUMC.

Chronic stress was the strongest predictor of SA (β = 0.643 [95% CI, 0.427-0.859]; *P* < .001), followed by ACEs (β = 0.240 [95% CI, 0.106-0.374]; *P* < .001) and VSAIL score (β = 0.162 [95% CI, 0.005-0.320]; *P* = .04) (Figure 2; eTable 3 in Supplement 1). Homelessness (β = 0.151 [95% CI, −0.026 to 0.329]; *P* = .10), financial insecurity (β = 0.124 [95% CI, −0.023 to 0.271]; *P* = .10), loneliness (β = 0.083 [95% CI, −0.045 to 0.211]; *P* = .20), and social isolation (β = 0.078 [95% CI, −0.074 to 0.231]; *P* = .31) were statistically insignificant.

## Discussion

This study aimed to evaluate whether augmenting risk score from a clinical data–based SA risk prediction model with clinical note–extracted psychosocial factors was associated with higher predictive performance, focusing on patients discharged from the ED after presentation for SI. Prognostication in this high-risk group remains an important challenge due to the difficulty of identifying individuals at imminent risk of suicide within a population sharing similar high-risk traits. To our knowledge, this study is the first to incorporate psychosocial factors derived from clinical notes via advanced NLP to improve suicide risk prediction. Incorporating psychosocial factors was associated with significantly higher model performance, with median AUROC increasing by 12% and AUPRC by 47% in VUH and median increasing AUROC by 24% and AUPRC by 86% in RHS. These performance increases were observed in both study sites, demonstrating reproducibility across different hospital settings.

Cox proportional hazards regression models trained solely on psychosocial factors performed comparably with those trained on both VSAIL score and psychosocial factors, suggesting that, within a population with ED visits for SI characterized by shared high suicidality, the clinical factor–based risk represented by VSAIL score does not meaningfully distinguish those who will attempt suicide in the near term. Instead, psychosocial factors, such as chronic stress and ACEs, better capture the underlying vulnerability that differentiates patients at high risk for SA. For ED patients screened for SI, effective stratification of SA risk may rely primarily on psychosocial factors, underscoring the importance of both systematic assessment of psychosocial factors and incorporating them into SA risk stratification.

Chronic stress emerged as the most important predictor of SA, aligning with existing literature demonstrating associations between chronic stress and suicidal crises across diverse populations.^40,41,42^ For instance, Bryan et al^40^ found that chronic, but not acute, stress is associated with SI and SA among US soldiers, and Pettit et al^42^ observed that chronic romantic stress was associated with SA among psychiatric inpatient adolescents. Furthermore, Grover et al^43^ highlighted the potential of problem-solving skills training, such as those provided through cognitive behavioral therapy,^44^ to reduce suicide risk among individuals experiencing chronic stress. These findings underscore the importance of screening for chronic stress for ED patients with SI and integrating problem-solving skills training into treatment plans as a targeted intervention.

A previous study by Wilimitis et al^37^ reported that VSAIL achieved an AUPRC of 0.235 for predicting SA within 30 days among psychiatric ED visits and an AUPRC of 0.007 among general ED visits. At VUH, VSAIL applied to ED visits for SI achieved an AUPRC of 0.083 for predicting SA within 90 days, aligning with anticipated performance. Our findings demonstrate that incorporating psychosocial factors alongside the VSAIL score (AUPRC, 0.122; PPV, 0.143) enhances predictive performance, bridging the gap toward published benchmarks for clinically actionable levels (PPV >0.2-0.3).^45^ The cohort here shared SI, placing them at higher risk than the prior study of a more general ED cohort. This improvement was achieved using only 6 psychosocial factors, suggesting the potential for further performance enhancement with the inclusion of additional factors.

Psychosocial factors are embedded within clinical notes, which poses significant challenges to their practical application. To translate our findings into practice, psychosocial factors must be accessible as structured fields to enable their integration into the existing automated suicide risk stratification workflows. Potential strategies include preextracting psychosocial factors from clinical notes and storing them in structured EHR fields. Although they lack the scalability of our NLP approach, large language models demonstrate strong performance in extracting psychosocial factors from clinical notes.^46,47,48^ This advancement provides an opportunity to translate findings into practice by addressing the challenges of unstructured data integration into clinical workflows.

### Limitations

This study has several limitations. First, our study populations reflected the predominantly White demographics of middle Tennessee, limiting generalizability, particularly because psychosocial factors may disproportionately affect underrepresented individuals. Second, our NLP approach does not inherently differentiate between historical and current SI and SA events. However, a prior validation study demonstrated an AUPRC of 0.77 for identifying current SA events.^28^ We calibrated the relevance score threshold to ensure a PPV of 0.90 for current SI and SA detection. Third, we included only 6 psychosocial factors, which were originally developed for a prior study on predicting unplanned hospitalizations and ED visits.^49^ Future work should include psychosocial factors such as an individual’s reasons for living (eg, sense of purpose, belonging, and responsibility to others). Although most research on suicide focuses on risk factors associated with SI and SA, complementary work emphasizes the importance of understanding what sustains individuals through suicidal crises.^12,50,51,52,53^ Fourth, we assigned a value of zero when psychosocial factor was not documented, recognizing that this may reflect unrecorded evidence rather than true absence, which could bias effect estimates. Fifth, prior studies suggest that integrating self-report surveys and/or clinician assessments with EHR data improves prediction of SA in the ED setting.^37,45,54^ However, the Columbia-Suicide Severity Rating Scale, used in VUMC EDs, was unavailable in the VUMC Synthetic Derivative data repository. Sixth, the EHR data capture only encounters within VUMC, limiting outcome ascertainment to SAs documented at VUMC.

## Conclusions

In this prognostic study of patients discharged from the ED after presentation for SI, suicide risk prediction models based solely on clinical structured EHR data showed limited predictive performance. Incorporating psychosocial factors extracted from clinical notes was associated with higher predictive performance, bringing it toward published benchmarks for clinically actionable levels. Chronic stress emerged as the strongest predictor in the augmented suicide risk prediction model. Identifying more suicide-relevant psychosocial factors will be crucial for further improving these models and achieving more accurate and clinically actionable suicide risk predictions.

## Notes

Supplement 1. **eFigure 1.** Flow Diagram Depicting Inclusion and Exclusion Criteria for ED Visits for SI **eFigure 2.** Density Plots of VSAIL and Log Transformed Psychosocial Factor Relevance Scores Stratified by ED Visits for SI With and Without Suicide Attempt Withing 90 Days **eTable 1.** *International Classification of Diseases* (*ICD*) Codes for Suicide Attempt **eTable 2.** AUROCs and AUPRCs (IQR) and Their Pairwise Statistical Comparison Between Data Modalities Using Bootstrap CIs **eTable 3.** Standardized β Coefficients and 95% CIs for Predictors of 90-Day Suicide Attempt From a Cox Proportional Hazards Regression Model

Supplement 2.
Data Sharing Statement
