# Supplementary material for: Clinical Note–Extracted Psychosocial Factors for Predicting Suicide Attempt Among ED Patients With Suicidal Ideation
Source: JAMA Netw Open. 2026 Mar 4;9(3):e260589. doi: 10.1001/jamanetworkopen.2026.0589 (PMC12961534; doi:10.1001/jamanetworkopen.2026.0589)
Supplement: Supplement 1. — eFigure 1. Flow Diagram Depicting Inclusion and Exclusion Criteria for ED Visits for SI eFigure 2. Density Plots of VSAIL and Log Transformed Psychosocial Factor Relevance Scores Stratified by ED Visits for SI With and Without Suicide Attempt Withing 90 Days eTable 1. International Classification of Diseases (ICD) Codes for Suicide Attempt eTable 2. AUROCs and AUPRCs (IQR) and Their Pairwise Statistical Comparison Between Data Modalities Using Bootstrap CIs eTable 3. Standardized β Coefficients and 95% CIs for Predictors of 90-Day Suicide Attempt From a Cox Proportional Hazards Regression Model [file jamanetwopen-e260589-s001.pdf]

Supplementary Online Content

Lee H, Jadhav K, Ripperger M, et al. Clinical note–extracted psychosocial factors for predicting suicide attempt among ED patients with suicidal ideation. *JAMA Netw Open.* 2026;9(3):e260589. doi:10.1001/jamanetworkopen.2026.0589

eFigure 1. Flow Diagram Depicting Inclusion and Exclusion Criteria for ED Visits for SI

eFigure 2. Density Plots of VSAIL and Log Transformed Psychosocial Factor Relevance Scores Stratified by ED Visits for SI With and Without Suicide Attempt Within 90 Days

eTable 1. *International Classification of Diseases* (*ICD*) Codes for Suicide Attempt

eTable 2. AUROCs and AUPRCs (IQR) and Their Pairwise Statistical Comparison Between Data Modalities Using Bootstrap CIs

eTable 3. Standardized β Coefficients and 95% CIs for Predictors of 90-Day Suicide Attempt From a Cox Proportional Hazards Regression Model

This supplementary material has been provided by the authors to give readers additional information about their work.

© 2026 Lee H et al. *JAMA Network Open.*

eFigure 1. Flow Diagram Depicting Inclusion and Exclusion Criteria for ED Visits for SI

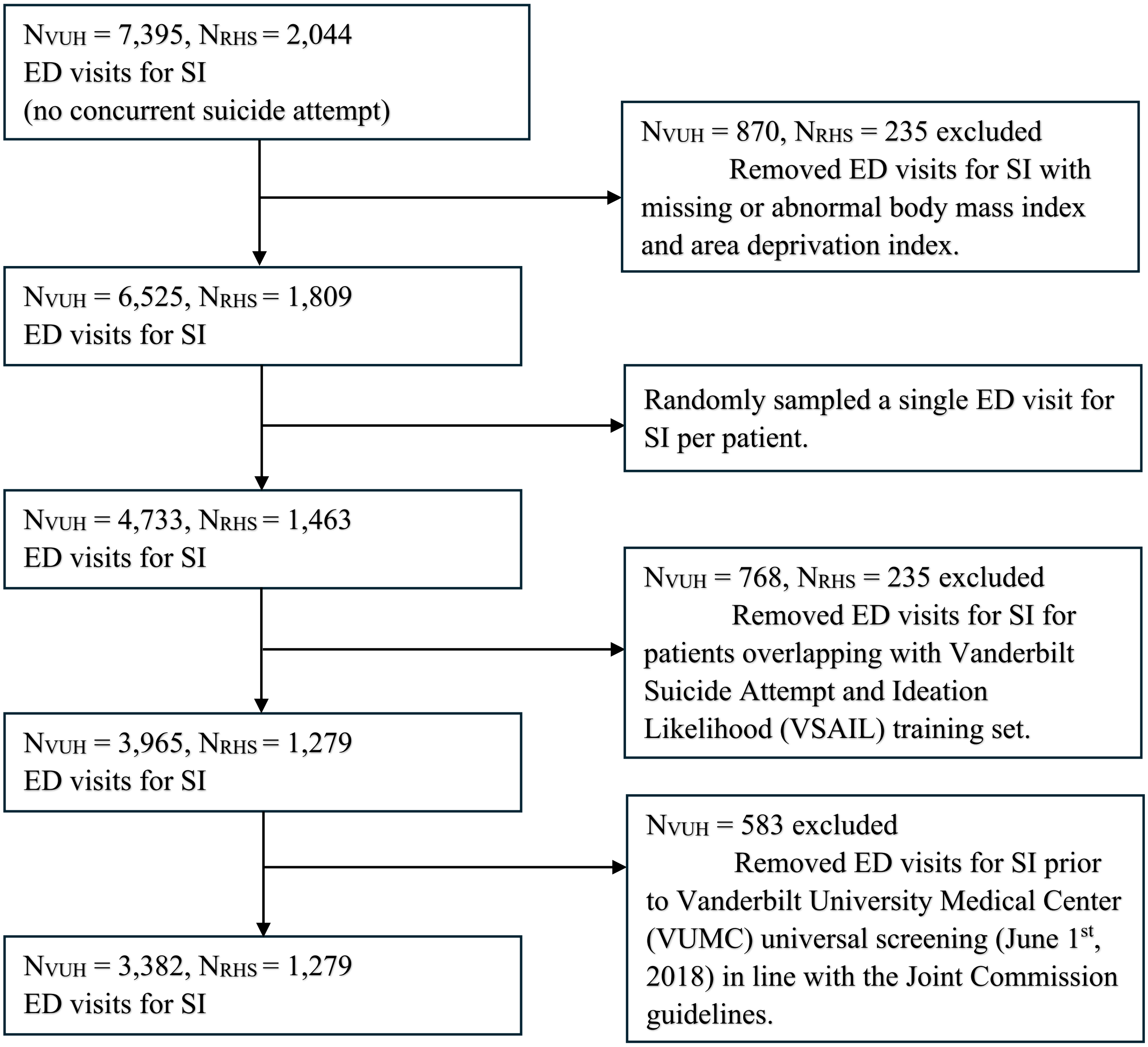

© 2026 Lee H et al. *JAMA Network Open.*

eFigure 2. Density Plots of VSAIL and Log Transformed Psychosocial Factor Relevance Scores Stratified by ED Visits for SI With and Without Suicide Attempt Within 90 Days.

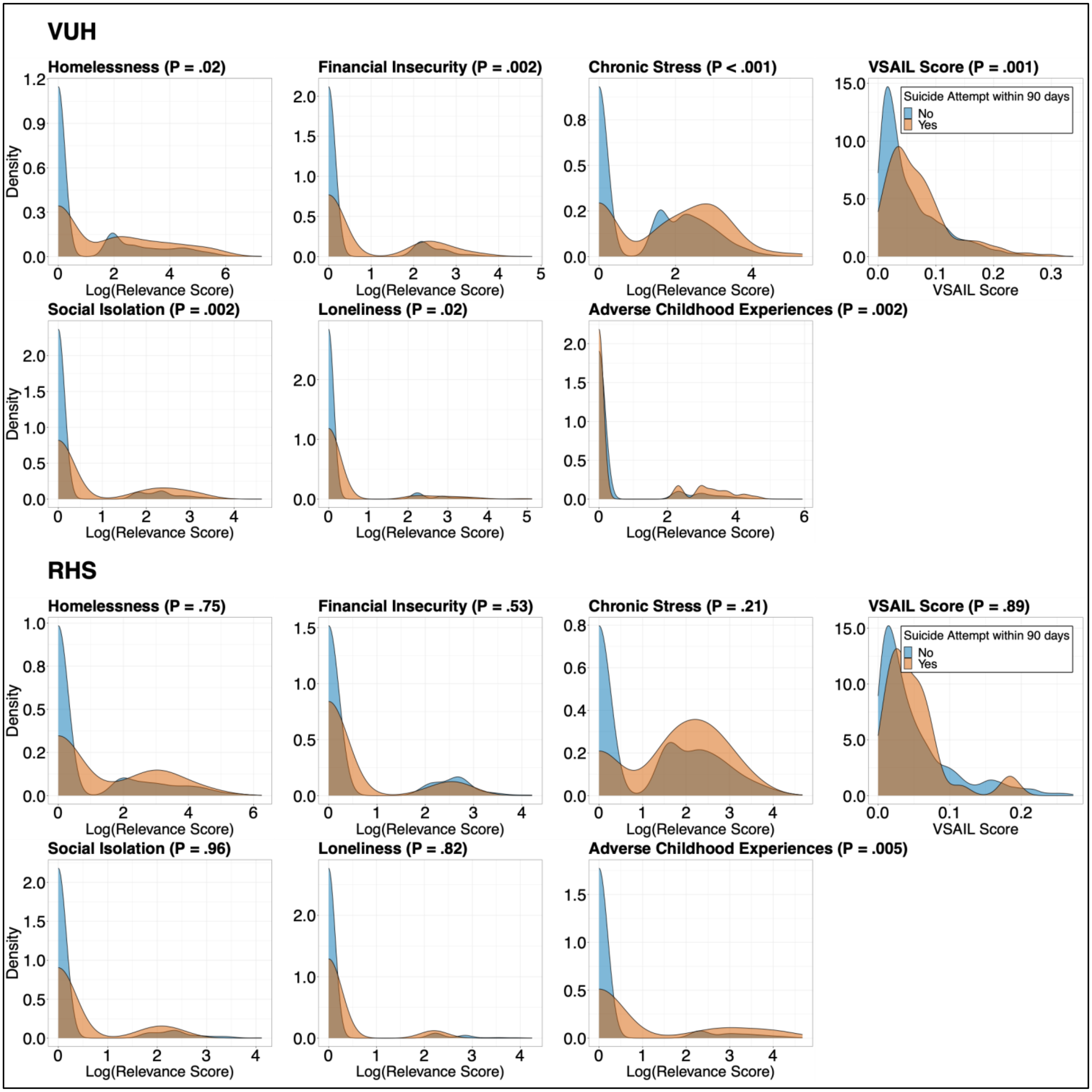

VUH: n = 3,382, Suicide Attempt within 90 Days = 160 [4.7%]; RHS: n = 1,279, Suicide Attempt within 90 Days = 34 [2.7%]. We performed two-sample permutation test by randomly shuffling outcome label (suicide attempt within 90 days) and recalculating the difference between groups 100,000 times. The P was then calculated as the proportion of shuffled differences that were as large as or larger than the observed difference.

© 2026 Lee H et al. *JAMA Network Open.*

eTable 1. *International Classification of Diseases* (*ICD*) Codes for Suicide Attempt

ICD CODE	CODE DESCRIPTION	VERSION
E950	Suicide and self-inflicted poisoning by solid or liquid substances	ICD9CM
E950.0	Suicide and self-inflicted poisoning by analgesics, antipyretics, and antirheumatics	ICD9CM
E950.1	Suicide and self-inflicted poisoning by barbiturates	ICD9CM
E950.2	Suicide and self-inflicted poisoning by other sedatives and hypnotics	ICD9CM
E950.3	Suicide and self-inflicted poisoning by tranquilizers and other psychotropic agents	ICD9CM
E950.4	Suicide and self-inflicted poisoning by other specified drugs and medicinal substances	ICD9CM
E950.5	Suicide and self-inflicted poisoning by unspecified drug or medicinal substance	ICD9CM
E950.6	Suicide and self-inflicted poisoning by agricultural and horticultural chemical and pharmaceutical preparations other than plant foods and fertilizers	ICD9CM
E950.7	Suicide and self-inflicted poisoning by corrosive and caustic substances	ICD9CM
E950.8	Suicide and self-inflicted poisoning by arsenic and its compounds	ICD9CM
E950.9	Suicide and self-inflicted poisoning by other and unspecified solid and liquid substances	ICD9CM
E951	Suicide and self-inflicted poisoning by gases in domestic use	ICD9CM
E951.0	Suicide and self-inflicted poisoning by gas distributed by pipeline	ICD9CM
E951.1	Suicide and self-inflicted poisoning by liquefied petroleum gas distributed in mobile containers	ICD9CM
E951.8	Suicide and self-inflicted poisoning by other utility gas	ICD9CM
E952	Suicide and self-inflicted poisoning by other gases and vapors	ICD9CM
E952.0	Suicide and self-inflicted poisoning by motor vehicle exhaust gas	ICD9CM
E952.1	Suicide and self-inflicted poisoning by other carbon monoxide	ICD9CM
E952.8	Suicide and self-inflicted poisoning by other specified gases and vapors	ICD9CM
E952.9	Suicide and self-inflicted poisoning by unspecified gases and vapors	ICD9CM
E953	Suicide and self-inflicted injury by hanging, strangulation, and suffocation	ICD9CM
E953.0	Suicide and self-inflicted injury by hanging	ICD9CM
E953.1	Suicide and self-inflicted injury by suffocation by plastic bag	ICD9CM
E953.8	Suicide and self-inflicted injury by other specified means	ICD9CM
E953.9	Suicide and self-inflicted injury by unspecified means	ICD9CM
E954	Suicide and self-inflicted injury by submersion [drowning]	ICD9CM
E955	Suicide and self-inflicted injury by firearms, air guns, and explosives	ICD9CM
E955.0	Suicide and self-inflicted injury by handgun	ICD9CM
E955.1	Suicide and self-inflicted injury by shotgun	ICD9CM
E955.2	Suicide and self-inflicted injury by hunting rifle	ICD9CM
E955.3	Suicide and self-inflicted injury by military firearms	ICD9CM
E955.4	Suicide and self-inflicted injury by other and unspecified firearm	ICD9CM
E955.5	Suicide and self-inflicted injury by explosives	ICD9CM
E955.6	Suicide and self-inflicted injury by air gun	ICD9CM
E955.7	Suicide and self-inflicted injury by paintball gun	ICD9CM
E955.9	Suicide and self-inflicted injury by firearms and explosives, unspecified	ICD9CM
E956	Suicide and self-inflicted injury by cutting and piercing instrument	ICD9CM
E957	Suicide and self-inflicted injuries by jumping from high place	ICD9CM
E957.0	Suicide and self-inflicted injuries by jumping from residential premises	ICD9CM
E957.1	Suicide and self-inflicted injuries by jumping from other man-made structures	ICD9CM

© 2026 Lee H et al. *JAMA Network Open.*

E957.2	Suicide and self-inflicted injuries by jumping from natural sites	ICD9CM
E957.9	Suicide and self-inflicted injuries by jumping from unspecified site	ICD9CM
E958	Suicide and self-inflicted injury by other and unspecified means	ICD9CM
E958.0	Suicide and self-inflicted injury by jumping or lying before moving object	ICD9CM
E958.1	Suicide and self-inflicted injury by burns, fire	ICD9CM
E958.2	Suicide and self-inflicted injury by scald	ICD9CM
E958.3	Suicide and self-inflicted injury by extremes of cold	ICD9CM
E958.4	Suicide and self-inflicted injury by electrocution	ICD9CM
E958.5	Suicide and self-inflicted injury by crashing of motor vehicle	ICD9CM
E958.6	Suicide and self-inflicted injury by crashing of aircraft	ICD9CM
E958.7	Suicide and self-inflicted injury by caustic substances, except poisoning	ICD9CM
E958.8	Suicide and self-inflicted injury by other specified means	ICD9CM
E958.9	Suicide and self-inflicted injury by unspecified means	ICD9CM
E959	Late effects of self-inflicted injury	ICD9CM
X60	Intentional self-poisoning by and exposure to nonopioid analgesics, antipyretics and antirheumatics	ICD10
X61	Intentional self-poisoning by and exposure to antiepileptic, sedative-hypnotic, antiparkinsonism and psychotropic drugs, not elsewhere classified	ICD10
X62	Intentional self-poisoning by and exposure to narcotics and psychodysleptics [hallucinogens], not elsewhere classified	ICD10
X63	Intentional self-poisoning by and exposure to other drugs acting on the autonomic nervous system	ICD10
X64	Intentional self-poisoning by and exposure to other and unspecified drugs, medicaments and biological substances	ICD10
X65	Intentional self-poisoning by and exposure to alcohol	ICD10
X66	Intentional self-poisoning by and exposure to organic solvents and halogenated hydrocarbons and their vapours	ICD10
X67	Intentional self-poisoning by and exposure to other gases and vapours	ICD10
X68	Intentional self-poisoning by and exposure to pesticides	ICD10
X69	Intentional self-poisoning by and exposure to other and unspecified chemicals and noxious substances	ICD10
X70	Intentional self-harm by hanging, strangulation and suffocation	ICD10
X71	Intentional self-harm by drowning and submersion	ICD10
X72	Intentional self-harm by handgun discharge	ICD10
X73	Intentional self-harm by rifle, shotgun and larger firearm discharge	ICD10
X74	Intentional self-harm by other and unspecified firearm discharge	ICD10
X75	Intentional self-harm by explosive material	ICD10
X76	Intentional self-harm by smoke, fire and flames	ICD10
X77	Intentional self-harm by steam, hot vapours and hot objects	ICD10
X78	Intentional self-harm by sharp object	ICD10
X79	Intentional self-harm by blunt object	ICD10
X80	Intentional self-harm by jumping from a high place	ICD10
X81	Intentional self-harm by jumping or lying before moving object	ICD10
X82	Intentional self-harm by crashing of motor vehicle	ICD10
X83	Intentional self-harm by other specified means	ICD10
X84	Intentional self-harm by unspecified means	ICD10

© 2026 Lee H et al. *JAMA Network Open.*

Y87.0	Sequelae of intentional self-harm	ICD10
T14.91	Suicide attempt	ICD10CM
T14.91XA	Suicide attempt, initial encounter	ICD10CM
T14.91XD	Suicide attempt, subsequent encounter	ICD10CM
T14.91XS	Suicide attempt, sequela	ICD10CM
T36.0X2	Poisoning by penicillins, intentional self-harm	ICD10CM
T36.0X2A	Poisoning by penicillins, intentional self-harm, initial encounter	ICD10CM
T36.0X2D	Poisoning by penicillins, intentional self-harm, subsequent encounter	ICD10CM
T36.0X2S	Poisoning by penicillins, intentional self-harm, sequela	ICD10CM
T36.1X2	Poisoning by cephalosporins and other beta-lactam antibiotics, intentional self-harm	ICD10CM
T36.1X2A	Poisoning by cephalosporins and other beta-lactam antibiotics, intentional self-harm, initial encounter	ICD10CM
T36.1X2D	Poisoning by cephalosporins and other beta-lactam antibiotics, intentional self-harm, subsequent encounter	ICD10CM
T36.1X2S	Poisoning by cephalosporins and other beta-lactam antibiotics, intentional self-harm, sequela	ICD10CM
T36.2X2	Poisoning by chloramphenicol group, intentional self-harm	ICD10CM
T36.2X2A	Poisoning by chloramphenicol group, intentional self-harm, initial encounter	ICD10CM
T36.2X2D	Poisoning by chloramphenicol group, intentional self-harm, subsequent encounter	ICD10CM
T36.2X2S	Poisoning by chloramphenicol group, intentional self-harm, sequela	ICD10CM
T36.3X2	Poisoning by macrolides, intentional self-harm	ICD10CM
T36.3X2A	Poisoning by macrolides, intentional self-harm, initial encounter	ICD10CM
T36.3X2D	Poisoning by macrolides, intentional self-harm, subsequent encounter	ICD10CM
T36.3X2S	Poisoning by macrolides, intentional self-harm, sequela	ICD10CM
T36.4X2	Poisoning by tetracyclines, intentional self-harm	ICD10CM
T36.4X2A	Poisoning by tetracyclines, intentional self-harm, initial encounter	ICD10CM
T36.4X2D	Poisoning by tetracyclines, intentional self-harm, subsequent encounter	ICD10CM
T36.4X2S	Poisoning by tetracyclines, intentional self-harm, sequela	ICD10CM
T36.5X2	Poisoning by aminoglycosides, intentional self-harm	ICD10CM
T36.5X2A	Poisoning by aminoglycosides, intentional self-harm, initial encounter	ICD10CM
T36.5X2D	Poisoning by aminoglycosides, intentional self-harm, subsequent encounter	ICD10CM
T36.5X2S	Poisoning by aminoglycosides, intentional self-harm, sequela	ICD10CM
T36.6X2	Poisoning by rifampicins, intentional self-harm	ICD10CM
T36.6X2A	Poisoning by rifampicins, intentional self-harm, initial encounter	ICD10CM
T36.6X2D	Poisoning by rifampicins, intentional self-harm, subsequent encounter	ICD10CM
T36.6X2S	Poisoning by rifampicins, intentional self-harm, sequela	ICD10CM
T36.7X2	Poisoning by antifungal antibiotics, systemically used, intentional self-harm	ICD10CM
T36.7X2A	Poisoning by antifungal antibiotics, systemically used, intentional self-harm, initial encounter	ICD10CM
T36.7X2D	Poisoning by antifungal antibiotics, systemically used, intentional self-harm, subsequent encounter	ICD10CM
T36.7X2S	Poisoning by antifungal antibiotics, systemically used, intentional self-harm, sequela	ICD10CM
T36.8X2	Poisoning by other systemic antibiotics, intentional self-harm	ICD10CM
T36.8X2A	Poisoning by other systemic antibiotics, intentional self-harm, initial encounter	ICD10CM
T36.8X2D	Poisoning by other systemic antibiotics, intentional self-harm, subsequent encounter	ICD10CM
T36.8X2S	Poisoning by other systemic antibiotics, intentional self-harm, sequela	ICD10CM

© 2026 Lee H et al. *JAMA Network Open.*

T36.92	Poisoning by unspecified systemic antibiotic, intentional self-harm	ICD10CM
T36.92XA	Poisoning by unspecified systemic antibiotic, intentional self-harm, initial encounter	ICD10CM
T36.92XD	Poisoning by unspecified systemic antibiotic, intentional self-harm, subsequent encounter	ICD10CM
T36.92XS	Poisoning by unspecified systemic antibiotic, intentional self-harm, sequela	ICD10CM
T37.0X2	Poisoning by sulfonamides, intentional self-harm	ICD10CM
T37.0X2A	Poisoning by sulfonamides, intentional self-harm, initial encounter	ICD10CM
T37.0X2D	Poisoning by sulfonamides, intentional self-harm, subsequent encounter	ICD10CM
T37.0X2S	Poisoning by sulfonamides, intentional self-harm, sequela	ICD10CM
T37.1X2	Poisoning by antimycobacterial drugs, intentional self-harm	ICD10CM
T37.1X2A	Poisoning by antimycobacterial drugs, intentional self-harm, initial encounter	ICD10CM
T37.1X2D	Poisoning by antimycobacterial drugs, intentional self-harm, subsequent encounter	ICD10CM
T37.1X2S	Poisoning by antimycobacterial drugs, intentional self-harm, sequela	ICD10CM
T37.2X2	Poisoning by antimalarials and drugs acting on other blood protozoa, intentional self-harm	ICD10CM
T37.2X2A	Poisoning by antimalarials and drugs acting on other blood protozoa, intentional self-harm, initial encounter	ICD10CM
T37.2X2D	Poisoning by antimalarials and drugs acting on other blood protozoa, intentional self-harm, subsequent encounter	ICD10CM
T37.2X2S	Poisoning by antimalarials and drugs acting on other blood protozoa, intentional self-harm, sequela	ICD10CM
T37.3X2	Poisoning by other antiprotozoal drugs, intentional self-harm	ICD10CM
T37.3X2A	Poisoning by other antiprotozoal drugs, intentional self-harm, initial encounter	ICD10CM
T37.3X2D	Poisoning by other antiprotozoal drugs, intentional self-harm, subsequent encounter	ICD10CM
T37.3X2S	Poisoning by other antiprotozoal drugs, intentional self-harm, sequela	ICD10CM
T37.4X2	Poisoning by anthelminthics, intentional self-harm	ICD10CM
T37.4X2A	Poisoning by anthelminthics, intentional self-harm, initial encounter	ICD10CM
T37.4X2D	Poisoning by anthelminthics, intentional self-harm, subsequent encounter	ICD10CM
T37.4X2S	Poisoning by anthelminthics, intentional self-harm, sequela	ICD10CM
T37.5X2	Poisoning by antiviral drugs, intentional self-harm	ICD10CM
T37.5X2A	Poisoning by antiviral drugs, intentional self-harm, initial encounter	ICD10CM
T37.5X2D	Poisoning by antiviral drugs, intentional self-harm, subsequent encounter	ICD10CM
T37.5X2S	Poisoning by antiviral drugs, intentional self-harm, sequela	ICD10CM
T37.8X2	Poisoning by other specified systemic anti-infectives and antiparasitics, intentional self-harm	ICD10CM
T37.8X2A	Poisoning by other specified systemic anti-infectives and antiparasitics, intentional self-harm, initial encounter	ICD10CM
T37.8X2D	Poisoning by other specified systemic anti-infectives and antiparasitics, intentional self-harm, subsequent encounter	ICD10CM
T37.8X2S	Poisoning by other specified systemic anti-infectives and antiparasitics, intentional self-harm, sequela	ICD10CM
T37.92	Poisoning by unspecified systemic anti-infective and antiparasitics, intentional self-harm	ICD10CM
T37.92XA	Poisoning by unspecified systemic anti-infective and antiparasitics, intentional self-harm, initial encounter	ICD10CM
T37.92XD	Poisoning by unspecified systemic anti-infective and antiparasitics, intentional self-harm, subsequent encounter	ICD10CM
T37.92XS	Poisoning by unspecified systemic anti-infective and antiparasitics, intentional self-harm, sequela	ICD10CM
T38.0X2	Poisoning by glucocorticoids and synthetic analogues, intentional self-harm	ICD10CM
T38.0X2A	Poisoning by glucocorticoids and synthetic analogues, intentional self-harm, initial encounter	ICD10CM

© 2026 Lee H et al. *JAMA Network Open.*

T38.0X2D	Poisoning by glucocorticoids and synthetic analogues, intentional self-harm, subsequent encounter	ICD10CM
T38.0X2S	Poisoning by glucocorticoids and synthetic analogues, intentional self-harm, sequela	ICD10CM
T38.1X2	Poisoning by thyroid hormones and substitutes, intentional self-harm	ICD10CM
T38.1X2A	Poisoning by thyroid hormones and substitutes, intentional self-harm, initial encounter	ICD10CM
T38.1X2D	Poisoning by thyroid hormones and substitutes, intentional self-harm, subsequent encounter	ICD10CM
T38.1X2S	Poisoning by thyroid hormones and substitutes, intentional self-harm, sequela	ICD10CM
T38.2X2	Poisoning by antithyroid drugs, intentional self-harm	ICD10CM
T38.2X2A	Poisoning by antithyroid drugs, intentional self-harm, initial encounter	ICD10CM
T38.2X2D	Poisoning by antithyroid drugs, intentional self-harm, subsequent encounter	ICD10CM
T38.2X2S	Poisoning by antithyroid drugs, intentional self-harm, sequela	ICD10CM
T38.3X2	Poisoning by insulin and oral hypoglycemic [antidiabetic] drugs, intentional self-harm	ICD10CM
T38.3X2A	Poisoning by insulin and oral hypoglycemic [antidiabetic] drugs, intentional self-harm, initial encounter	ICD10CM
T38.3X2D	Poisoning by insulin and oral hypoglycemic [antidiabetic] drugs, intentional self-harm, subsequent encounter	ICD10CM
T38.3X2S	Poisoning by insulin and oral hypoglycemic [antidiabetic] drugs, intentional self-harm, sequela	ICD10CM
T38.4X2	Poisoning by oral contraceptives, intentional self-harm	ICD10CM
T38.4X2A	Poisoning by oral contraceptives, intentional self-harm, initial encounter	ICD10CM
T38.4X2D	Poisoning by oral contraceptives, intentional self-harm, subsequent encounter	ICD10CM
T38.4X2S	Poisoning by oral contraceptives, intentional self-harm, sequela	ICD10CM
T38.5X2	Poisoning by other estrogens and progestogens, intentional self-harm	ICD10CM
T38.5X2A	Poisoning by other estrogens and progestogens, intentional self-harm, initial encounter	ICD10CM
T38.5X2D	Poisoning by other estrogens and progestogens, intentional self-harm, subsequent encounter	ICD10CM
T38.5X2S	Poisoning by other estrogens and progestogens, intentional self-harm, sequela	ICD10CM
T38.6X2	Poisoning by antigonadotrophins, antiestrogens, antiandrogens, not elsewhere classified, intentional self-harm	ICD10CM
T38.6X2A	Poisoning by antigonadotrophins, antiestrogens, antiandrogens, not elsewhere classified, intentional self-harm, initial encounter	ICD10CM
T38.6X2D	Poisoning by antigonadotrophins, antiestrogens, antiandrogens, not elsewhere classified, intentional self-harm, subsequent encounter	ICD10CM
T38.6X2S	Poisoning by antigonadotrophins, antiestrogens, antiandrogens, not elsewhere classified, intentional self-harm, sequela	ICD10CM
T38.7X2	Poisoning by androgens and anabolic congeners, intentional self-harm	ICD10CM
T38.7X2A	Poisoning by androgens and anabolic congeners, intentional self-harm, initial encounter	ICD10CM
T38.7X2D	Poisoning by androgens and anabolic congeners, intentional self-harm, subsequent encounter	ICD10CM
T38.7X2S	Poisoning by androgens and anabolic congeners, intentional self-harm, sequela	ICD10CM
T38.802	Poisoning by unspecified hormones and synthetic substitutes, intentional self-harm	ICD10CM
T38.802A	Poisoning by unspecified hormones and synthetic substitutes, intentional self-harm, initial encounter	ICD10CM
T38.802D	Poisoning by unspecified hormones and synthetic substitutes, intentional self-harm, subsequent encounter	ICD10CM
T38.802S	Poisoning by unspecified hormones and synthetic substitutes, intentional self-harm, sequela	ICD10CM
T38.812	Poisoning by anterior pituitary [adenohypophyseal] hormones, intentional self-harm	ICD10CM
T38.812A	Poisoning by anterior pituitary [adenohypophyseal] hormones, intentional self-harm, initial encounter	ICD10CM
T38.812D	Poisoning by anterior pituitary [adenohypophyseal] hormones, intentional self-harm, subsequent encounter	ICD10CM
T38.812S	Poisoning by anterior pituitary [adenohypophyseal] hormones, intentional self-harm, sequela	ICD10CM

© 2026 Lee H et al. *JAMA Network Open.*

T38.892	Poisoning by other hormones and synthetic substitutes, intentional self-harm	ICD10CM
T38.892A	Poisoning by other hormones and synthetic substitutes, intentional self-harm, initial encounter	ICD10CM
T38.892D	Poisoning by other hormones and synthetic substitutes, intentional self-harm, subsequent encounter	ICD10CM
T38.892S	Poisoning by other hormones and synthetic substitutes, intentional self-harm, sequela	ICD10CM
T38.902	Poisoning by unspecified hormone antagonists, intentional self-harm	ICD10CM
T38.902A	Poisoning by unspecified hormone antagonists, intentional self-harm, initial encounter	ICD10CM
T38.902D	Poisoning by unspecified hormone antagonists, intentional self-harm, subsequent encounter	ICD10CM
T38.902S	Poisoning by unspecified hormone antagonists, intentional self-harm, sequela	ICD10CM
T38.992	Poisoning by other hormone antagonists, intentional self-harm	ICD10CM
T38.992A	Poisoning by other hormone antagonists, intentional self-harm, initial encounter	ICD10CM
T38.992D	Poisoning by other hormone antagonists, intentional self-harm, subsequent encounter	ICD10CM
T38.992S	Poisoning by other hormone antagonists, intentional self-harm, sequela	ICD10CM
T39.012	Poisoning by aspirin, intentional self-harm	ICD10CM
T39.012A	Poisoning by aspirin, intentional self-harm, initial encounter	ICD10CM
T39.012D	Poisoning by aspirin, intentional self-harm, subsequent encounter	ICD10CM
T39.012S	Poisoning by aspirin, intentional self-harm, sequela	ICD10CM
T39.092	Poisoning by salicylates, intentional self-harm	ICD10CM
T39.092A	Poisoning by salicylates, intentional self-harm, initial encounter	ICD10CM
T39.092D	Poisoning by salicylates, intentional self-harm, subsequent encounter	ICD10CM
T39.092S	Poisoning by salicylates, intentional self-harm, sequela	ICD10CM
T39.1X2	Poisoning by 4-Aminophenol derivatives, intentional self-harm	ICD10CM
T39.1X2A	Poisoning by 4-Aminophenol derivatives, intentional self-harm, initial encounter	ICD10CM
T39.1X2D	Poisoning by 4-Aminophenol derivatives, intentional self-harm, subsequent encounter	ICD10CM
T39.1X2S	Poisoning by 4-Aminophenol derivatives, intentional self-harm, sequela	ICD10CM
T39.2X2	Poisoning by pyrazolone derivatives, intentional self-harm	ICD10CM
T39.2X2A	Poisoning by pyrazolone derivatives, intentional self-harm, initial encounter	ICD10CM
T39.2X2D	Poisoning by pyrazolone derivatives, intentional self-harm, subsequent encounter	ICD10CM
T39.2X2S	Poisoning by pyrazolone derivatives, intentional self-harm, sequela	ICD10CM
T39.312	Poisoning by propionic acid derivatives, intentional self-harm	ICD10CM
T39.312A	Poisoning by propionic acid derivatives, intentional self-harm, initial encounter	ICD10CM
T39.312D	Poisoning by propionic acid derivatives, intentional self-harm, subsequent encounter	ICD10CM
T39.312S	Poisoning by propionic acid derivatives, intentional self-harm, sequela	ICD10CM
T39.392	Poisoning by other nonsteroidal anti-inflammatory drugs [NSAID], intentional self-harm	ICD10CM
T39.392A	Poisoning by other nonsteroidal anti-inflammatory drugs [NSAID], intentional self-harm, initial encounter	ICD10CM
T39.392D	Poisoning by other nonsteroidal anti-inflammatory drugs [NSAID], intentional self-harm, subsequent encounter	ICD10CM
T39.392S	Poisoning by other nonsteroidal anti-inflammatory drugs [NSAID], intentional self-harm, sequela	ICD10CM
T39.4X2	Poisoning by antirheumatics, not elsewhere classified, intentional self-harm	ICD10CM
T39.4X2A	Poisoning by antirheumatics, not elsewhere classified, intentional self-harm, initial encounter	ICD10CM
T39.4X2D	Poisoning by antirheumatics, not elsewhere classified, intentional self-harm, subsequent encounter	ICD10CM
T39.4X2S	Poisoning by antirheumatics, not elsewhere classified, intentional self-harm, sequela	ICD10CM

© 2026 Lee H et al. *JAMA Network Open.*

T39.8X2	Poisoning by other nonopioid analgesics and antipyretics, not elsewhere classified, intentional self-harm	ICD10CM
T39.8X2A	Poisoning by other nonopioid analgesics and antipyretics, not elsewhere classified, intentional self-harm, initial encounter	ICD10CM
T39.8X2D	Poisoning by other nonopioid analgesics and antipyretics, not elsewhere classified, intentional self-harm, subsequent encounter	ICD10CM
T39.8X2S	Poisoning by other nonopioid analgesics and antipyretics, not elsewhere classified, intentional self-harm, sequela	ICD10CM
T39.92	Poisoning by unspecified nonopioid analgesic, antipyretic and antirheumatic, intentional self-harm	ICD10CM
T39.92XA	Poisoning by unspecified nonopioid analgesic, antipyretic and antirheumatic, intentional self-harm, initial encounter	ICD10CM
T39.92XD	Poisoning by unspecified nonopioid analgesic, antipyretic and antirheumatic, intentional self-harm, subsequent encounter	ICD10CM
T39.92XS	Poisoning by unspecified nonopioid analgesic, antipyretic and antirheumatic, intentional self-harm, sequela	ICD10CM
T40.0X2	Poisoning by opium, intentional self-harm	ICD10CM
T40.0X2A	Poisoning by opium, intentional self-harm, initial encounter	ICD10CM
T40.0X2D	Poisoning by opium, intentional self-harm, subsequent encounter	ICD10CM
T40.0X2S	Poisoning by opium, intentional self-harm, sequela	ICD10CM
T40.1X2	Poisoning by heroin, intentional self-harm	ICD10CM
T40.1X2A	Poisoning by heroin, intentional self-harm, initial encounter	ICD10CM
T40.1X2D	Poisoning by heroin, intentional self-harm, subsequent encounter	ICD10CM
T40.1X2S	Poisoning by heroin, intentional self-harm, sequela	ICD10CM
T40.2X2	Poisoning by other opioids, intentional self-harm	ICD10CM
T40.2X2A	Poisoning by other opioids, intentional self-harm, initial encounter	ICD10CM
T40.2X2D	Poisoning by other opioids, intentional self-harm, subsequent encounter	ICD10CM
T40.2X2S	Poisoning by other opioids, intentional self-harm, sequela	ICD10CM
T40.3X2	Poisoning by methadone, intentional self-harm	ICD10CM
T40.3X2A	Poisoning by methadone, intentional self-harm, initial encounter	ICD10CM
T40.3X2D	Poisoning by methadone, intentional self-harm, subsequent encounter	ICD10CM
T40.3X2S	Poisoning by methadone, intentional self-harm, sequela	ICD10CM
T40.4X2	Poisoning by other synthetic narcotics, intentional self-harm	ICD10CM
T40.4X2A	Poisoning by other synthetic narcotics, intentional self-harm, initial encounter	ICD10CM
T40.4X2D	Poisoning by other synthetic narcotics, intentional self-harm, subsequent encounter	ICD10CM
T40.4X2S	Poisoning by other synthetic narcotics, intentional self-harm, sequela	ICD10CM
T40.5X2	Poisoning by cocaine, intentional self-harm	ICD10CM
T40.5X2A	Poisoning by cocaine, intentional self-harm, initial encounter	ICD10CM
T40.5X2D	Poisoning by cocaine, intentional self-harm, subsequent encounter	ICD10CM
T40.5X2S	Poisoning by cocaine, intentional self-harm, sequela	ICD10CM
T40.602	Poisoning by unspecified narcotics, intentional self-harm	ICD10CM
T40.602A	Poisoning by unspecified narcotics, intentional self-harm, initial encounter	ICD10CM
T40.602D	Poisoning by unspecified narcotics, intentional self-harm, subsequent encounter	ICD10CM
T40.602S	Poisoning by unspecified narcotics, intentional self-harm, sequela	ICD10CM
T40.692	Poisoning by other narcotics, intentional self-harm	ICD10CM
T40.692A	Poisoning by other narcotics, intentional self-harm, initial encounter	ICD10CM
T40.692D	Poisoning by other narcotics, intentional self-harm, subsequent encounter	ICD10CM

© 2026 Lee H et al. *JAMA Network Open.*

T40.692S	Poisoning by other narcotics, intentional self-harm, sequela	ICD10CM
T40.7X2	Poisoning by cannabis (derivatives), intentional self-harm	ICD10CM
T40.7X2A	Poisoning by cannabis (derivatives), intentional self-harm, initial encounter	ICD10CM
T40.7X2D	Poisoning by cannabis (derivatives), intentional self-harm, subsequent encounter	ICD10CM
T40.7X2S	Poisoning by cannabis (derivatives), intentional self-harm, sequela	ICD10CM
T40.8X2	Poisoning by lysergide [LSD], intentional self-harm	ICD10CM
T40.8X2A	Poisoning by lysergide [LSD], intentional self-harm, initial encounter	ICD10CM
T40.8X2D	Poisoning by lysergide [LSD], intentional self-harm, subsequent encounter	ICD10CM
T40.8X2S	Poisoning by lysergide [LSD], intentional self-harm, sequela	ICD10CM
T40.902	Poisoning by unspecified psychodysleptics [hallucinogens], intentional self-harm	ICD10CM
T40.902A	Poisoning by unspecified psychodysleptics [hallucinogens], intentional self-harm, initial encounter	ICD10CM
T40.902D	Poisoning by unspecified psychodysleptics [hallucinogens], intentional self-harm, subsequent encounter	ICD10CM
T40.902S	Poisoning by unspecified psychodysleptics [hallucinogens], intentional self-harm, sequela	ICD10CM
T40.992	Poisoning by other psychodysleptics [hallucinogens], intentional self-harm	ICD10CM
T40.992A	Poisoning by other psychodysleptics [hallucinogens], intentional self-harm, initial encounter	ICD10CM
T40.992D	Poisoning by other psychodysleptics [hallucinogens], intentional self-harm, subsequent encounter	ICD10CM
T40.992S	Poisoning by other psychodysleptics [hallucinogens], intentional self-harm, sequela	ICD10CM
T41.0X2	Poisoning by inhaled anesthetics, intentional self-harm	ICD10CM
T41.0X2A	Poisoning by inhaled anesthetics, intentional self-harm, initial encounter	ICD10CM
T41.0X2D	Poisoning by inhaled anesthetics, intentional self-harm, subsequent encounter	ICD10CM
T41.0X2S	Poisoning by inhaled anesthetics, intentional self-harm, sequela	ICD10CM
T41.1X2	Poisoning by intravenous anesthetics, intentional self-harm	ICD10CM
T41.1X2A	Poisoning by intravenous anesthetics, intentional self-harm, initial encounter	ICD10CM
T41.1X2D	Poisoning by intravenous anesthetics, intentional self-harm, subsequent encounter	ICD10CM
T41.1X2S	Poisoning by intravenous anesthetics, intentional self-harm, sequela	ICD10CM
T41.202	Poisoning by unspecified general anesthetics, intentional self-harm	ICD10CM
T41.202A	Poisoning by unspecified general anesthetics, intentional self-harm, initial encounter	ICD10CM
T41.202D	Poisoning by unspecified general anesthetics, intentional self-harm, subsequent encounter	ICD10CM
T41.202S	Poisoning by unspecified general anesthetics, intentional self-harm, sequela	ICD10CM
T41.292	Poisoning by other general anesthetics, intentional self-harm	ICD10CM
T41.292A	Poisoning by other general anesthetics, intentional self-harm, initial encounter	ICD10CM
T41.292D	Poisoning by other general anesthetics, intentional self-harm, subsequent encounter	ICD10CM
T41.292S	Poisoning by other general anesthetics, intentional self-harm, sequela	ICD10CM
T41.3X2	Poisoning by local anesthetics, intentional self-harm	ICD10CM
T41.3X2A	Poisoning by local anesthetics, intentional self-harm, initial encounter	ICD10CM
T41.3X2D	Poisoning by local anesthetics, intentional self-harm, subsequent encounter	ICD10CM
T41.3X2S	Poisoning by local anesthetics, intentional self-harm, sequela	ICD10CM
T41.42	Poisoning by unspecified anesthetic, intentional self-harm	ICD10CM
T41.42XA	Poisoning by unspecified anesthetic, intentional self-harm, initial encounter	ICD10CM
T41.42XD	Poisoning by unspecified anesthetic, intentional self-harm, subsequent encounter	ICD10CM
T41.42XS	Poisoning by unspecified anesthetic, intentional self-harm, sequela	ICD10CM

© 2026 Lee H et al. *JAMA Network Open.*

T41.5X2	Poisoning by therapeutic gases, intentional self-harm	ICD10CM
T41.5X2A	Poisoning by therapeutic gases, intentional self-harm, initial encounter	ICD10CM
T41.5X2D	Poisoning by therapeutic gases, intentional self-harm, subsequent encounter	ICD10CM
T41.5X2S	Poisoning by therapeutic gases, intentional self-harm, sequela	ICD10CM
T42.0X2	Poisoning by hydantoin derivatives, intentional self-harm	ICD10CM
T42.0X2A	Poisoning by hydantoin derivatives, intentional self-harm, initial encounter	ICD10CM
T42.0X2D	Poisoning by hydantoin derivatives, intentional self-harm, subsequent encounter	ICD10CM
T42.0X2S	Poisoning by hydantoin derivatives, intentional self-harm, sequela	ICD10CM
T42.1X2	Poisoning by iminostilbenes, intentional self-harm	ICD10CM
T42.1X2A	Poisoning by iminostilbenes, intentional self-harm, initial encounter	ICD10CM
T42.1X2D	Poisoning by iminostilbenes, intentional self-harm, subsequent encounter	ICD10CM
T42.1X2S	Poisoning by iminostilbenes, intentional self-harm, sequela	ICD10CM
T42.2X2	Poisoning by succinimides and oxazolidinediones, intentional self-harm	ICD10CM
T42.2X2A	Poisoning by succinimides and oxazolidinediones, intentional self-harm, initial encounter	ICD10CM
T42.2X2D	Poisoning by succinimides and oxazolidinediones, intentional self-harm, subsequent encounter	ICD10CM
T42.2X2S	Poisoning by succinimides and oxazolidinediones, intentional self-harm, sequela	ICD10CM
T42.3X2	Poisoning by barbiturates, intentional self-harm	ICD10CM
T42.3X2A	Poisoning by barbiturates, intentional self-harm, initial encounter	ICD10CM
T42.3X2D	Poisoning by barbiturates, intentional self-harm, subsequent encounter	ICD10CM
T42.3X2S	Poisoning by barbiturates, intentional self-harm, sequela	ICD10CM
T42.4X2	Poisoning by benzodiazepines, intentional self-harm	ICD10CM
T42.4X2A	Poisoning by benzodiazepines, intentional self-harm, initial encounter	ICD10CM
T42.4X2D	Poisoning by benzodiazepines, intentional self-harm, subsequent encounter	ICD10CM
T42.4X2S	Poisoning by benzodiazepines, intentional self-harm, sequela	ICD10CM
T42.5X2	Poisoning by mixed antiepileptics, intentional self-harm	ICD10CM
T42.5X2A	Poisoning by mixed antiepileptics, intentional self-harm, initial encounter	ICD10CM
T42.5X2D	Poisoning by mixed antiepileptics, intentional self-harm, subsequent encounter	ICD10CM
T42.5X2S	Poisoning by mixed antiepileptics, intentional self-harm, sequela	ICD10CM
T42.6X2	Poisoning by other antiepileptic and sedative-hypnotic drugs, intentional self-harm	ICD10CM
T42.6X2A	Poisoning by other antiepileptic and sedative-hypnotic drugs, intentional self-harm, initial encounter	ICD10CM
T42.6X2D	Poisoning by other antiepileptic and sedative-hypnotic drugs, intentional self-harm, subsequent encounter	ICD10CM
T42.6X2S	Poisoning by other antiepileptic and sedative-hypnotic drugs, intentional self-harm, sequela	ICD10CM
T42.72	Poisoning by unspecified antiepileptic and sedative-hypnotic drugs, intentional self-harm	ICD10CM
T42.72XA	Poisoning by unspecified antiepileptic and sedative-hypnotic drugs, intentional self-harm, initial encounter	ICD10CM
T42.72XD	Poisoning by unspecified antiepileptic and sedative-hypnotic drugs, intentional self-harm, subsequent encounter	ICD10CM
T42.72XS	Poisoning by unspecified antiepileptic and sedative-hypnotic drugs, intentional self-harm, sequela	ICD10CM
T42.8X2	Poisoning by antiparkinsonism drugs and other central muscle-tone depressants, intentional self-harm	ICD10CM
T42.8X2A	Poisoning by antiparkinsonism drugs and other central muscle-tone depressants, intentional self-harm, initial encounter	ICD10CM
T42.8X2D	Poisoning by antiparkinsonism drugs and other central muscle-tone depressants, intentional self-harm, subsequent encounter	ICD10CM

© 2026 Lee H et al. *JAMA Network Open.*

T42.8X2S	Poisoning by antiparkinsonism drugs and other central muscle-tone depressants, intentional self-harm, sequela	ICD10CM
T43.012	Poisoning by tricyclic antidepressants, intentional self-harm	ICD10CM
T43.012A	Poisoning by tricyclic antidepressants, intentional self-harm, initial encounter	ICD10CM
T43.012D	Poisoning by tricyclic antidepressants, intentional self-harm, subsequent encounter	ICD10CM
T43.012S	Poisoning by tricyclic antidepressants, intentional self-harm, sequela	ICD10CM
T43.022	Poisoning by tetracyclic antidepressants, intentional self-harm	ICD10CM
T43.022A	Poisoning by tetracyclic antidepressants, intentional self-harm, initial encounter	ICD10CM
T43.022D	Poisoning by tetracyclic antidepressants, intentional self-harm, subsequent encounter	ICD10CM
T43.022S	Poisoning by tetracyclic antidepressants, intentional self-harm, sequela	ICD10CM
T43.0x2A	Poisoning by tricyclic and tetracyclic antidepressants, intentional self-harm, initial encounter	ICD10CM
T43.0x2D	Poisoning by tricyclic and tetracyclic antidepressants, intentional self-harm, subsequent encounter	ICD10CM
T43.0x2S	Poisoning by tricyclic and tetracyclic antidepressants, intentional self-harm, sequela	ICD10CM
T43.1X2	Poisoning by monoamine-oxidase-inhibitor antidepressants, intentional self-harm	ICD10CM
T43.1X2A	Poisoning by monoamine-oxidase-inhibitor antidepressants, intentional self-harm, initial encounter	ICD10CM
T43.1X2D	Poisoning by monoamine-oxidase-inhibitor antidepressants, intentional self-harm, subsequent encounter	ICD10CM
T43.1X2S	Poisoning by monoamine-oxidase-inhibitor antidepressants, intentional self-harm, sequela	ICD10CM
T43.202	Poisoning by unspecified antidepressants, intentional self-harm	ICD10CM
T43.202A	Poisoning by unspecified antidepressants, intentional self-harm, initial encounter	ICD10CM
T43.202D	Poisoning by unspecified antidepressants, intentional self-harm, subsequent encounter	ICD10CM
T43.202S	Poisoning by unspecified antidepressants, intentional self-harm, sequela	ICD10CM
T43.212	Poisoning by selective serotonin and norepinephrine reuptake inhibitors, intentional self-harm	ICD10CM
T43.212A	Poisoning by selective serotonin and norepinephrine reuptake inhibitors, intentional self-harm, initial encounter	ICD10CM
T43.212D	Poisoning by selective serotonin and norepinephrine reuptake inhibitors, intentional self-harm, subsequent encounter	ICD10CM
T43.212S	Poisoning by selective serotonin and norepinephrine reuptake inhibitors, intentional self-harm, sequela	ICD10CM
T43.222	Poisoning by selective serotonin reuptake inhibitors, intentional self-harm	ICD10CM
T43.222A	Poisoning by selective serotonin reuptake inhibitors, intentional self-harm, initial encounter	ICD10CM
T43.222D	Poisoning by selective serotonin reuptake inhibitors, intentional self-harm, subsequent encounter	ICD10CM
T43.222S	Poisoning by selective serotonin reuptake inhibitors, intentional self-harm, sequela	ICD10CM
T43.292	Poisoning by other antidepressants, intentional self-harm	ICD10CM
T43.292A	Poisoning by other antidepressants, intentional self-harm, initial encounter	ICD10CM
T43.292D	Poisoning by other antidepressants, intentional self-harm, subsequent encounter	ICD10CM
T43.292S	Poisoning by other antidepressants, intentional self-harm, sequela	ICD10CM
T43.3X2	Poisoning by phenothiazine antipsychotics and neuroleptics, intentional self-harm	ICD10CM
T43.3X2A	Poisoning by phenothiazine antipsychotics and neuroleptics, intentional self-harm, initial encounter	ICD10CM
T43.3X2D	Poisoning by phenothiazine antipsychotics and neuroleptics, intentional self-harm, subsequent encounter	ICD10CM
T43.3X2S	Poisoning by phenothiazine antipsychotics and neuroleptics, intentional self-harm, sequela	ICD10CM
T43.4X2	Poisoning by butyrophenone and thiothixene neuroleptics, intentional self-harm	ICD10CM
T43.4X2A	Poisoning by butyrophenone and thiothixene neuroleptics, intentional self-harm, initial encounter	ICD10CM

© 2026 Lee H et al. *JAMA Network Open.*

T43.4X2D	Poisoning by butyrophenone and thiothixene neuroleptics, intentional self-harm, subsequent encounter	ICD10CM
T43.4X2S	Poisoning by butyrophenone and thiothixene neuroleptics, intentional self-harm, sequela	ICD10CM
T43.502	Poisoning by unspecified antipsychotics and neuroleptics, intentional self-harm	ICD10CM
T43.502A	Poisoning by unspecified antipsychotics and neuroleptics, intentional self-harm, initial encounter	ICD10CM
T43.502D	Poisoning by unspecified antipsychotics and neuroleptics, intentional self-harm, subsequent encounter	ICD10CM
T43.502S	Poisoning by unspecified antipsychotics and neuroleptics, intentional self-harm, sequela	ICD10CM
T43.592	Poisoning by other antipsychotics and neuroleptics, intentional self-harm	ICD10CM
T43.592A	Poisoning by other antipsychotics and neuroleptics, intentional self-harm, initial encounter	ICD10CM
T43.592D	Poisoning by other antipsychotics and neuroleptics, intentional self-harm, subsequent encounter	ICD10CM
T43.592S	Poisoning by other antipsychotics and neuroleptics, intentional self-harm, sequela	ICD10CM
T43.602	Poisoning by unspecified psychostimulants, intentional self-harm	ICD10CM
T43.602A	Poisoning by unspecified psychostimulants, intentional self-harm, initial encounter	ICD10CM
T43.602D	Poisoning by unspecified psychostimulants, intentional self-harm, subsequent encounter	ICD10CM
T43.602S	Poisoning by unspecified psychostimulants, intentional self-harm, sequela	ICD10CM
T43.612	Poisoning by caffeine, intentional self-harm	ICD10CM
T43.612A	Poisoning by caffeine, intentional self-harm, initial encounter	ICD10CM
T43.612D	Poisoning by caffeine, intentional self-harm, subsequent encounter	ICD10CM
T43.612S	Poisoning by caffeine, intentional self-harm, sequela	ICD10CM
T43.622	Poisoning by amphetamines, intentional self-harm	ICD10CM
T43.622A	Poisoning by amphetamines, intentional self-harm, initial encounter	ICD10CM
T43.622D	Poisoning by amphetamines, intentional self-harm, subsequent encounter	ICD10CM
T43.622S	Poisoning by amphetamines, intentional self-harm, sequela	ICD10CM
T43.632	Poisoning by methylphenidate, intentional self-harm	ICD10CM
T43.632A	Poisoning by methylphenidate, intentional self-harm, initial encounter	ICD10CM
T43.632D	Poisoning by methylphenidate, intentional self-harm, subsequent encounter	ICD10CM
T43.632S	Poisoning by methylphenidate, intentional self-harm, sequela	ICD10CM
T43.642	Poisoning by ecstasy, intentional self-harm	ICD10CM
T43.642A	Poisoning by ecstasy, intentional self-harm, initial encounter	ICD10CM
T43.642D	Poisoning by ecstasy, intentional self-harm, subsequent encounter	ICD10CM
T43.642S	Poisoning by ecstasy, intentional self-harm, sequela	ICD10CM
T43.692	Poisoning by other psychostimulants, intentional self-harm	ICD10CM
T43.692A	Poisoning by other psychostimulants, intentional self-harm, initial encounter	ICD10CM
T43.692D	Poisoning by other psychostimulants, intentional self-harm, subsequent encounter	ICD10CM
T43.692S	Poisoning by other psychostimulants, intentional self-harm, sequela	ICD10CM
T43.6x2A	Poisoning by psychostimulants with abuse potential, intentional self-harm, initial encounter	ICD10CM
T43.6x2D	Poisoning by psychostimulants with abuse potential, intentional self-harm, subsequent encounter	ICD10CM
T43.6x2S	Poisoning by psychostimulants with abuse potential, intentional self-harm, sequela	ICD10CM
T43.8X2	Poisoning by other psychotropic drugs, intentional self-harm	ICD10CM
T43.8X2A	Poisoning by other psychotropic drugs, intentional self-harm, initial encounter	ICD10CM
T43.8X2D	Poisoning by other psychotropic drugs, intentional self-harm, subsequent encounter	ICD10CM
T43.8X2S	Poisoning by other psychotropic drugs, intentional self-harm, sequela	ICD10CM

© 2026 Lee H et al. *JAMA Network Open.*

T43.92	Poisoning by unspecified psychotropic drug, intentional self-harm	ICD10CM
T43.92XA	Poisoning by unspecified psychotropic drug, intentional self-harm, initial encounter	ICD10CM
T43.92XD	Poisoning by unspecified psychotropic drug, intentional self-harm, subsequent encounter	ICD10CM
T43.92XS	Poisoning by unspecified psychotropic drug, intentional self-harm, sequela	ICD10CM
T44.0X2	Poisoning by anticholinesterase agents, intentional self-harm	ICD10CM
T44.0X2A	Poisoning by anticholinesterase agents, intentional self-harm, initial encounter	ICD10CM
T44.0X2D	Poisoning by anticholinesterase agents, intentional self-harm, subsequent encounter	ICD10CM
T44.0X2S	Poisoning by anticholinesterase agents, intentional self-harm, sequela	ICD10CM
T44.1X2	Poisoning by other parasympathomimetics [cholinergics], intentional self-harm	ICD10CM
T44.1X2A	Poisoning by other parasympathomimetics [cholinergics], intentional self-harm, initial encounter	ICD10CM
T44.1X2D	Poisoning by other parasympathomimetics [cholinergics], intentional self-harm, subsequent encounter	ICD10CM
T44.1X2S	Poisoning by other parasympathomimetics [cholinergics], intentional self-harm, sequela	ICD10CM
T44.2X2	Poisoning by ganglionic blocking drugs, intentional self-harm	ICD10CM
T44.2X2A	Poisoning by ganglionic blocking drugs, intentional self-harm, initial encounter	ICD10CM
T44.2X2D	Poisoning by ganglionic blocking drugs, intentional self-harm, subsequent encounter	ICD10CM
T44.2X2S	Poisoning by ganglionic blocking drugs, intentional self-harm, sequela	ICD10CM
T44.3X2	Poisoning by other parasympatholytics [anticholinergics and antimuscarinics] and spasmolytics, intentional self-harm	ICD10CM
T44.3X2A	Poisoning by other parasympatholytics [anticholinergics and antimuscarinics] and spasmolytics, intentional self-harm, initial encounter	ICD10CM
T44.3X2D	Poisoning by other parasympatholytics [anticholinergics and antimuscarinics] and spasmolytics, intentional self-harm, subsequent encounter	ICD10CM
T44.3X2S	Poisoning by other parasympatholytics [anticholinergics and antimuscarinics] and spasmolytics, intentional self-harm, sequela	ICD10CM
T44.4X2	Poisoning by predominantly alpha-adrenoreceptor agonists, intentional self-harm	ICD10CM
T44.4X2A	Poisoning by predominantly alpha-adrenoreceptor agonists, intentional self-harm, initial encounter	ICD10CM
T44.4X2D	Poisoning by predominantly alpha-adrenoreceptor agonists, intentional self-harm, subsequent encounter	ICD10CM
T44.4X2S	Poisoning by predominantly alpha-adrenoreceptor agonists, intentional self-harm, sequela	ICD10CM
T44.5X2	Poisoning by predominantly beta-adrenoreceptor agonists, intentional self-harm	ICD10CM
T44.5X2A	Poisoning by predominantly beta-adrenoreceptor agonists, intentional self-harm, initial encounter	ICD10CM
T44.5X2D	Poisoning by predominantly beta-adrenoreceptor agonists, intentional self-harm, subsequent encounter	ICD10CM
T44.5X2S	Poisoning by predominantly beta-adrenoreceptor agonists, intentional self-harm, sequela	ICD10CM
T44.6X2	Poisoning by alpha-adrenoreceptor antagonists, intentional self-harm	ICD10CM
T44.6X2A	Poisoning by alpha-adrenoreceptor antagonists, intentional self-harm, initial encounter	ICD10CM
T44.6X2D	Poisoning by alpha-adrenoreceptor antagonists, intentional self-harm, subsequent encounter	ICD10CM
T44.6X2S	Poisoning by alpha-adrenoreceptor antagonists, intentional self-harm, sequela	ICD10CM
T44.7X2	Poisoning by beta-adrenoreceptor antagonists, intentional self-harm	ICD10CM
T44.7X2A	Poisoning by beta-adrenoreceptor antagonists, intentional self-harm, initial encounter	ICD10CM
T44.7X2D	Poisoning by beta-adrenoreceptor antagonists, intentional self-harm, subsequent encounter	ICD10CM
T44.7X2S	Poisoning by beta-adrenoreceptor antagonists, intentional self-harm, sequela	ICD10CM
T44.8X2	Poisoning by centrally-acting and adrenergic-neuron-blocking agents, intentional self-harm	ICD10CM
T44.8X2A	Poisoning by centrally-acting and adrenergic-neuron-blocking agents, intentional self-harm, initial encounter	ICD10CM

© 2026 Lee H et al. *JAMA Network Open.*

T44.8X2D	Poisoning by centrally-acting and adrenergic-neuron-blocking agents, intentional self-harm, subsequent encounter	ICD10CM
T44.8X2S	Poisoning by centrally-acting and adrenergic-neuron-blocking agents, intentional self-harm, sequela	ICD10CM
T44.902	Poisoning by unspecified drugs primarily affecting the autonomic nervous system, intentional self-harm	ICD10CM
T44.902A	Poisoning by unspecified drugs primarily affecting the autonomic nervous system, intentional self-harm, initial encounter	ICD10CM
T44.902D	Poisoning by unspecified drugs primarily affecting the autonomic nervous system, intentional self-harm, subsequent encounter	ICD10CM
T44.902S	Poisoning by unspecified drugs primarily affecting the autonomic nervous system, intentional self-harm, sequela	ICD10CM
T44.992	Poisoning by other drug primarily affecting the autonomic nervous system, intentional self-harm	ICD10CM
T44.992A	Poisoning by other drug primarily affecting the autonomic nervous system, intentional self-harm, initial encounter	ICD10CM
T44.992D	Poisoning by other drug primarily affecting the autonomic nervous system, intentional self-harm, subsequent encounter	ICD10CM
T44.992S	Poisoning by other drug primarily affecting the autonomic nervous system, intentional self-harm, sequela	ICD10CM
T45.0X2	Poisoning by antiallergic and antiemetic drugs, intentional self-harm	ICD10CM
T45.0X2A	Poisoning by antiallergic and antiemetic drugs, intentional self-harm, initial encounter	ICD10CM
T45.0X2D	Poisoning by antiallergic and antiemetic drugs, intentional self-harm, subsequent encounter	ICD10CM
T45.0X2S	Poisoning by antiallergic and antiemetic drugs, intentional self-harm, sequela	ICD10CM
T45.1X2	Poisoning by antineoplastic and immunosuppressive drugs, intentional self-harm	ICD10CM
T45.1X2A	Poisoning by antineoplastic and immunosuppressive drugs, intentional self-harm, initial encounter	ICD10CM
T45.1X2D	Poisoning by antineoplastic and immunosuppressive drugs, intentional self-harm, subsequent encounter	ICD10CM
T45.1X2S	Poisoning by antineoplastic and immunosuppressive drugs, intentional self-harm, sequela	ICD10CM
T45.2X2	Poisoning by vitamins, intentional self-harm	ICD10CM
T45.2X2A	Poisoning by vitamins, intentional self-harm, initial encounter	ICD10CM
T45.2X2D	Poisoning by vitamins, intentional self-harm, subsequent encounter	ICD10CM
T45.2X2S	Poisoning by vitamins, intentional self-harm, sequela	ICD10CM
T45.3X2	Poisoning by enzymes, intentional self-harm	ICD10CM
T45.3X2A	Poisoning by enzymes, intentional self-harm, initial encounter	ICD10CM
T45.3X2D	Poisoning by enzymes, intentional self-harm, subsequent encounter	ICD10CM
T45.3X2S	Poisoning by enzymes, intentional self-harm, sequela	ICD10CM
T45.4X2	Poisoning by iron and its compounds, intentional self-harm	ICD10CM
T45.4X2A	Poisoning by iron and its compounds, intentional self-harm, initial encounter	ICD10CM
T45.4X2D	Poisoning by iron and its compounds, intentional self-harm, subsequent encounter	ICD10CM
T45.4X2S	Poisoning by iron and its compounds, intentional self-harm, sequela	ICD10CM
T45.512	Poisoning by anticoagulants, intentional self-harm	ICD10CM
T45.512A	Poisoning by anticoagulants, intentional self-harm, initial encounter	ICD10CM
T45.512D	Poisoning by anticoagulants, intentional self-harm, subsequent encounter	ICD10CM
T45.512S	Poisoning by anticoagulants, intentional self-harm, sequela	ICD10CM
T45.522	Poisoning by antithrombotic drugs, intentional self-harm	ICD10CM
T45.522A	Poisoning by antithrombotic drugs, intentional self-harm, initial encounter	ICD10CM
T45.522D	Poisoning by antithrombotic drugs, intentional self-harm, subsequent encounter	ICD10CM
T45.522S	Poisoning by antithrombotic drugs, intentional self-harm, sequela	ICD10CM

© 2026 Lee H et al. *JAMA Network Open.*

T45.602	Poisoning by unspecified fibrinolysis-affecting drugs, intentional self-harm	ICD10CM
T45.602A	Poisoning by unspecified fibrinolysis-affecting drugs, intentional self-harm, initial encounter	ICD10CM
T45.602D	Poisoning by unspecified fibrinolysis-affecting drugs, intentional self-harm, subsequent encounter	ICD10CM
T45.602S	Poisoning by unspecified fibrinolysis-affecting drugs, intentional self-harm, sequela	ICD10CM
T45.612	Poisoning by thrombolytic drug, intentional self-harm	ICD10CM
T45.612A	Poisoning by thrombolytic drug, intentional self-harm, initial encounter	ICD10CM
T45.612D	Poisoning by thrombolytic drug, intentional self-harm, subsequent encounter	ICD10CM
T45.612S	Poisoning by thrombolytic drug, intentional self-harm, sequela	ICD10CM
T45.622	Poisoning by hemostatic drug, intentional self-harm	ICD10CM
T45.622A	Poisoning by hemostatic drug, intentional self-harm, initial encounter	ICD10CM
T45.622D	Poisoning by hemostatic drug, intentional self-harm, subsequent encounter	ICD10CM
T45.622S	Poisoning by hemostatic drug, intentional self-harm, sequela	ICD10CM
T45.692	Poisoning by other fibrinolysis-affecting drugs, intentional self-harm	ICD10CM
T45.692A	Poisoning by other fibrinolysis-affecting drugs, intentional self-harm, initial encounter	ICD10CM
T45.692D	Poisoning by other fibrinolysis-affecting drugs, intentional self-harm, subsequent encounter	ICD10CM
T45.692S	Poisoning by other fibrinolysis-affecting drugs, intentional self-harm, sequela	ICD10CM
T45.7X2	Poisoning by anticoagulant antagonists, vitamin K and other coagulants, intentional self-harm	ICD10CM
T45.7X2A	Poisoning by anticoagulant antagonists, vitamin K and other coagulants, intentional self-harm, initial encounter	ICD10CM
T45.7X2D	Poisoning by anticoagulant antagonists, vitamin K and other coagulants, intentional self-harm, subsequent encounter	ICD10CM
T45.7X2S	Poisoning by anticoagulant antagonists, vitamin K and other coagulants, intentional self-harm, sequela	ICD10CM
T45.8X2	Poisoning by other primarily systemic and hematological agents, intentional self-harm	ICD10CM
T45.8X2A	Poisoning by other primarily systemic and hematological agents, intentional self-harm, initial encounter	ICD10CM
T45.8X2D	Poisoning by other primarily systemic and hematological agents, intentional self-harm, subsequent encounter	ICD10CM
T45.8X2S	Poisoning by other primarily systemic and hematological agents, intentional self-harm, sequela	ICD10CM
T45.92	Poisoning by unspecified primarily systemic and hematological agent, intentional self-harm	ICD10CM
T45.92XA	Poisoning by unspecified primarily systemic and hematological agent, intentional self-harm, initial encounter	ICD10CM
T45.92XD	Poisoning by unspecified primarily systemic and hematological agent, intentional self-harm, subsequent encounter	ICD10CM
T45.92XS	Poisoning by unspecified primarily systemic and hematological agent, intentional self-harm, sequela	ICD10CM
T46.0X2	Poisoning by cardiac-stimulant glycosides and drugs of similar action, intentional self-harm	ICD10CM
T46.0X2A	Poisoning by cardiac-stimulant glycosides and drugs of similar action, intentional self-harm, initial encounter	ICD10CM
T46.0X2D	Poisoning by cardiac-stimulant glycosides and drugs of similar action, intentional self-harm, subsequent encounter	ICD10CM
T46.0X2S	Poisoning by cardiac-stimulant glycosides and drugs of similar action, intentional self-harm, sequela	ICD10CM
T46.1X2	Poisoning by calcium-channel blockers, intentional self-harm	ICD10CM
T46.1X2A	Poisoning by calcium-channel blockers, intentional self-harm, initial encounter	ICD10CM
T46.1X2D	Poisoning by calcium-channel blockers, intentional self-harm, subsequent encounter	ICD10CM
T46.1X2S	Poisoning by calcium-channel blockers, intentional self-harm, sequela	ICD10CM
T46.2X2	Poisoning by other antidysrhythmic drugs, intentional self-harm	ICD10CM

© 2026 Lee H et al. *JAMA Network Open.*

T46.2X2A	Poisoning by other antidysrhythmic drugs, intentional self-harm, initial encounter	ICD10CM
T46.2X2D	Poisoning by other antidysrhythmic drugs, intentional self-harm, subsequent encounter	ICD10CM
T46.2X2S	Poisoning by other antidysrhythmic drugs, intentional self-harm, sequela	ICD10CM
T46.3X2	Poisoning by coronary vasodilators, intentional self-harm	ICD10CM
T46.3X2A	Poisoning by coronary vasodilators, intentional self-harm, initial encounter	ICD10CM
T46.3X2D	Poisoning by coronary vasodilators, intentional self-harm, subsequent encounter	ICD10CM
T46.3X2S	Poisoning by coronary vasodilators, intentional self-harm, sequela	ICD10CM
T46.4X2	Poisoning by angiotensin-converting-enzyme inhibitors, intentional self-harm	ICD10CM
T46.4X2A	Poisoning by angiotensin-converting-enzyme inhibitors, intentional self-harm, initial encounter	ICD10CM
T46.4X2D	Poisoning by angiotensin-converting-enzyme inhibitors, intentional self-harm, subsequent encounter	ICD10CM
T46.4X2S	Poisoning by angiotensin-converting-enzyme inhibitors, intentional self-harm, sequela	ICD10CM
T46.5X2	Poisoning by other antihypertensive drugs, intentional self-harm	ICD10CM
T46.5X2A	Poisoning by other antihypertensive drugs, intentional self-harm, initial encounter	ICD10CM
T46.5X2D	Poisoning by other antihypertensive drugs, intentional self-harm, subsequent encounter	ICD10CM
T46.5X2S	Poisoning by other antihypertensive drugs, intentional self-harm, sequela	ICD10CM
T46.6X2	Poisoning by antihyperlipidemic and antiarteriosclerotic drugs, intentional self-harm	ICD10CM
T46.6X2A	Poisoning by antihyperlipidemic and antiarteriosclerotic drugs, intentional self-harm, initial encounter	ICD10CM
T46.6X2D	Poisoning by antihyperlipidemic and antiarteriosclerotic drugs, intentional self-harm, subsequent encounter	ICD10CM
T46.6X2S	Poisoning by antihyperlipidemic and antiarteriosclerotic drugs, intentional self-harm, sequela	ICD10CM
T46.7X2	Poisoning by peripheral vasodilators, intentional self-harm	ICD10CM
T46.7X2A	Poisoning by peripheral vasodilators, intentional self-harm, initial encounter	ICD10CM
T46.7X2D	Poisoning by peripheral vasodilators, intentional self-harm, subsequent encounter	ICD10CM
T46.7X2S	Poisoning by peripheral vasodilators, intentional self-harm, sequela	ICD10CM
T46.8X2	Poisoning by antivaricose drugs, including sclerosing agents, intentional self-harm	ICD10CM
T46.8X2A	Poisoning by antivaricose drugs, including sclerosing agents, intentional self-harm, initial encounter	ICD10CM
T46.8X2D	Poisoning by antivaricose drugs, including sclerosing agents, intentional self-harm, subsequent encounter	ICD10CM
T46.8X2S	Poisoning by antivaricose drugs, including sclerosing agents, intentional self-harm, sequela	ICD10CM
T46.902	Poisoning by unspecified agents primarily affecting the cardiovascular system, intentional self-harm	ICD10CM
T46.902A	Poisoning by unspecified agents primarily affecting the cardiovascular system, intentional self-harm, initial encounter	ICD10CM
T46.902D	Poisoning by unspecified agents primarily affecting the cardiovascular system, intentional self-harm, subsequent encounter	ICD10CM
T46.902S	Poisoning by unspecified agents primarily affecting the cardiovascular system, intentional self-harm, sequela	ICD10CM
T46.992	Poisoning by other agents primarily affecting the cardiovascular system, intentional self-harm	ICD10CM
T46.992A	Poisoning by other agents primarily affecting the cardiovascular system, intentional self-harm, initial encounter	ICD10CM
T46.992D	Poisoning by other agents primarily affecting the cardiovascular system, intentional self-harm, subsequent encounter	ICD10CM
T46.992S	Poisoning by other agents primarily affecting the cardiovascular system, intentional self-harm, sequela	ICD10CM
T47.0X2	Poisoning by histamine H2-receptor blockers, intentional self-harm	ICD10CM
T47.0X2A	Poisoning by histamine H2-receptor blockers, intentional self-harm, initial encounter	ICD10CM

© 2026 Lee H et al. *JAMA Network Open.*

T47.0X2D	Poisoning by histamine H2-receptor blockers, intentional self-harm, subsequent encounter	ICD10CM
T47.0X2S	Poisoning by histamine H2-receptor blockers, intentional self-harm, sequela	ICD10CM
T47.1X2	Poisoning by other antacids and anti-gastric-secretion drugs, intentional self-harm	ICD10CM
T47.1X2A	Poisoning by other antacids and anti-gastric-secretion drugs, intentional self-harm, initial encounter	ICD10CM
T47.1X2D	Poisoning by other antacids and anti-gastric-secretion drugs, intentional self-harm, subsequent encounter	ICD10CM
T47.1X2S	Poisoning by other antacids and anti-gastric-secretion drugs, intentional self-harm, sequela	ICD10CM
T47.2X2	Poisoning by stimulant laxatives, intentional self-harm	ICD10CM
T47.2X2A	Poisoning by stimulant laxatives, intentional self-harm, initial encounter	ICD10CM
T47.2X2D	Poisoning by stimulant laxatives, intentional self-harm, subsequent encounter	ICD10CM
T47.2X2S	Poisoning by stimulant laxatives, intentional self-harm, sequela	ICD10CM
T47.3X2	Poisoning by saline and osmotic laxatives, intentional self-harm	ICD10CM
T47.3X2A	Poisoning by saline and osmotic laxatives, intentional self-harm, initial encounter	ICD10CM
T47.3X2D	Poisoning by saline and osmotic laxatives, intentional self-harm, subsequent encounter	ICD10CM
T47.3X2S	Poisoning by saline and osmotic laxatives, intentional self-harm, sequela	ICD10CM
T47.4X2	Poisoning by other laxatives, intentional self-harm	ICD10CM
T47.4X2A	Poisoning by other laxatives, intentional self-harm, initial encounter	ICD10CM
T47.4X2D	Poisoning by other laxatives, intentional self-harm, subsequent encounter	ICD10CM
T47.4X2S	Poisoning by other laxatives, intentional self-harm, sequela	ICD10CM
T47.5X2	Poisoning by digestants, intentional self-harm	ICD10CM
T47.5X2A	Poisoning by digestants, intentional self-harm, initial encounter	ICD10CM
T47.5X2D	Poisoning by digestants, intentional self-harm, subsequent encounter	ICD10CM
T47.5X2S	Poisoning by digestants, intentional self-harm, sequela	ICD10CM
T47.6X2	Poisoning by antidiarrheal drugs, intentional self-harm	ICD10CM
T47.6X2A	Poisoning by antidiarrheal drugs, intentional self-harm, initial encounter	ICD10CM
T47.6X2D	Poisoning by antidiarrheal drugs, intentional self-harm, subsequent encounter	ICD10CM
T47.6X2S	Poisoning by antidiarrheal drugs, intentional self-harm, sequela	ICD10CM
T47.7X2	Poisoning by emetics, intentional self-harm	ICD10CM
T47.7X2A	Poisoning by emetics, intentional self-harm, initial encounter	ICD10CM
T47.7X2D	Poisoning by emetics, intentional self-harm, subsequent encounter	ICD10CM
T47.7X2S	Poisoning by emetics, intentional self-harm, sequela	ICD10CM
T47.8X2	Poisoning by other agents primarily affecting gastrointestinal system, intentional self-harm	ICD10CM
T47.8X2A	Poisoning by other agents primarily affecting gastrointestinal system, intentional self-harm, initial encounter	ICD10CM
T47.8X2D	Poisoning by other agents primarily affecting gastrointestinal system, intentional self-harm, subsequent encounter	ICD10CM
T47.8X2S	Poisoning by other agents primarily affecting gastrointestinal system, intentional self-harm, sequela	ICD10CM
T47.92	Poisoning by unspecified agents primarily affecting the gastrointestinal system, intentional self-harm	ICD10CM
T47.92XA	Poisoning by unspecified agents primarily affecting the gastrointestinal system, intentional self-harm, initial encounter	ICD10CM
T47.92XD	Poisoning by unspecified agents primarily affecting the gastrointestinal system, intentional self-harm, subsequent encounter	ICD10CM
T47.92XS	Poisoning by unspecified agents primarily affecting the gastrointestinal system, intentional self-harm, sequela	ICD10CM

© 2026 Lee H et al. *JAMA Network Open.*

T48.0X2	Poisoning by oxytocic drugs, intentional self-harm	ICD10CM
T48.0X2A	Poisoning by oxytocic drugs, intentional self-harm, initial encounter	ICD10CM
T48.0X2D	Poisoning by oxytocic drugs, intentional self-harm, subsequent encounter	ICD10CM
T48.0X2S	Poisoning by oxytocic drugs, intentional self-harm, sequela	ICD10CM
T48.1X2	Poisoning by skeletal muscle relaxants [neuromuscular blocking agents], intentional self-harm	ICD10CM
T48.1X2A	Poisoning by skeletal muscle relaxants [neuromuscular blocking agents], intentional self-harm, initial encounter	ICD10CM
T48.1X2D	Poisoning by skeletal muscle relaxants [neuromuscular blocking agents], intentional self-harm, subsequent encounter	ICD10CM
T48.1X2S	Poisoning by skeletal muscle relaxants [neuromuscular blocking agents], intentional self-harm, sequela	ICD10CM
T48.202	Poisoning by unspecified drugs acting on muscles, intentional self-harm	ICD10CM
T48.202A	Poisoning by unspecified drugs acting on muscles, intentional self-harm, initial encounter	ICD10CM
T48.202D	Poisoning by unspecified drugs acting on muscles, intentional self-harm, subsequent encounter	ICD10CM
T48.202S	Poisoning by unspecified drugs acting on muscles, intentional self-harm, sequela	ICD10CM
T48.292	Poisoning by other drugs acting on muscles, intentional self-harm	ICD10CM
T48.292A	Poisoning by other drugs acting on muscles, intentional self-harm, initial encounter	ICD10CM
T48.292D	Poisoning by other drugs acting on muscles, intentional self-harm, subsequent encounter	ICD10CM
T48.292S	Poisoning by other drugs acting on muscles, intentional self-harm, sequela	ICD10CM
T48.3X2	Poisoning by antitussives, intentional self-harm	ICD10CM
T48.3X2A	Poisoning by antitussives, intentional self-harm, initial encounter	ICD10CM
T48.3X2D	Poisoning by antitussives, intentional self-harm, subsequent encounter	ICD10CM
T48.3X2S	Poisoning by antitussives, intentional self-harm, sequela	ICD10CM
T48.4X2	Poisoning by expectorants, intentional self-harm	ICD10CM
T48.4X2A	Poisoning by expectorants, intentional self-harm, initial encounter	ICD10CM
T48.4X2D	Poisoning by expectorants, intentional self-harm, subsequent encounter	ICD10CM
T48.4X2S	Poisoning by expectorants, intentional self-harm, sequela	ICD10CM
T48.5X2	Poisoning by other anti-common-cold drugs, intentional self-harm	ICD10CM
T48.5X2A	Poisoning by other anti-common-cold drugs, intentional self-harm, initial encounter	ICD10CM
T48.5X2D	Poisoning by other anti-common-cold drugs, intentional self-harm, subsequent encounter	ICD10CM
T48.5X2S	Poisoning by other anti-common-cold drugs, intentional self-harm, sequela	ICD10CM
T48.6X2	Poisoning by antiasthmatics, intentional self-harm	ICD10CM
T48.6X2A	Poisoning by antiasthmatics, intentional self-harm, initial encounter	ICD10CM
T48.6X2D	Poisoning by antiasthmatics, intentional self-harm, subsequent encounter	ICD10CM
T48.6X2S	Poisoning by antiasthmatics, intentional self-harm, sequela	ICD10CM
T48.902	Poisoning by unspecified agents primarily acting on the respiratory system, intentional self-harm	ICD10CM
T48.902A	Poisoning by unspecified agents primarily acting on the respiratory system, intentional self-harm, initial encounter	ICD10CM
T48.902D	Poisoning by unspecified agents primarily acting on the respiratory system, intentional self-harm, subsequent encounter	ICD10CM
T48.902S	Poisoning by unspecified agents primarily acting on the respiratory system, intentional self-harm, sequela	ICD10CM
T48.992	Poisoning by other agents primarily acting on the respiratory system, intentional self-harm	ICD10CM
T48.992A	Poisoning by other agents primarily acting on the respiratory system, intentional self-harm, initial encounter	ICD10CM
T48.992D	Poisoning by other agents primarily acting on the respiratory system, intentional self-harm, subsequent encounter	ICD10CM

© 2026 Lee H et al. *JAMA Network Open.*

T48.992S	Poisoning by other agents primarily acting on the respiratory system, intentional self-harm, sequela	ICD10CM
T49.0X2	Poisoning by local antifungal, anti-infective and anti-inflammatory drugs, intentional self-harm	ICD10CM
T49.0X2A	Poisoning by local antifungal, anti-infective and anti-inflammatory drugs, intentional self-harm, initial encounter	ICD10CM
T49.0X2D	Poisoning by local antifungal, anti-infective and anti-inflammatory drugs, intentional self-harm, subsequent encounter	ICD10CM
T49.0X2S	Poisoning by local antifungal, anti-infective and anti-inflammatory drugs, intentional self-harm, sequela	ICD10CM
T49.1X2	Poisoning by antipruritics, intentional self-harm	ICD10CM
T49.1X2A	Poisoning by antipruritics, intentional self-harm, initial encounter	ICD10CM
T49.1X2D	Poisoning by antipruritics, intentional self-harm, subsequent encounter	ICD10CM
T49.1X2S	Poisoning by antipruritics, intentional self-harm, sequela	ICD10CM
T49.2X2	Poisoning by local astringents and local detergents, intentional self-harm	ICD10CM
T49.2X2A	Poisoning by local astringents and local detergents, intentional self-harm, initial encounter	ICD10CM
T49.2X2D	Poisoning by local astringents and local detergents, intentional self-harm, subsequent encounter	ICD10CM
T49.2X2S	Poisoning by local astringents and local detergents, intentional self-harm, sequela	ICD10CM
T49.3X2	Poisoning by emollients, demulcents and protectants, intentional self-harm	ICD10CM
T49.3X2A	Poisoning by emollients, demulcents and protectants, intentional self-harm, initial encounter	ICD10CM
T49.3X2D	Poisoning by emollients, demulcents and protectants, intentional self-harm, subsequent encounter	ICD10CM
T49.3X2S	Poisoning by emollients, demulcents and protectants, intentional self-harm, sequela	ICD10CM
T49.4X2	Poisoning by keratolytics, keratoplastics, and other hair treatment drugs and preparations, intentional self-harm	ICD10CM
T49.4X2A	Poisoning by keratolytics, keratoplastics, and other hair treatment drugs and preparations, intentional self-harm, initial encounter	ICD10CM
T49.4X2D	Poisoning by keratolytics, keratoplastics, and other hair treatment drugs and preparations, intentional self-harm, subsequent encounter	ICD10CM
T49.4X2S	Poisoning by keratolytics, keratoplastics, and other hair treatment drugs and preparations, intentional self-harm, sequela	ICD10CM
T49.5X2	Poisoning by ophthalmological drugs and preparations, intentional self-harm	ICD10CM
T49.5X2A	Poisoning by ophthalmological drugs and preparations, intentional self-harm, initial encounter	ICD10CM
T49.5X2D	Poisoning by ophthalmological drugs and preparations, intentional self-harm, subsequent encounter	ICD10CM
T49.5X2S	Poisoning by ophthalmological drugs and preparations, intentional self-harm, sequela	ICD10CM
T49.6X2	Poisoning by otorhinolaryngological drugs and preparations, intentional self-harm	ICD10CM
T49.6X2A	Poisoning by otorhinolaryngological drugs and preparations, intentional self-harm, initial encounter	ICD10CM
T49.6X2D	Poisoning by otorhinolaryngological drugs and preparations, intentional self-harm, subsequent encounter	ICD10CM
T49.6X2S	Poisoning by otorhinolaryngological drugs and preparations, intentional self-harm, sequela	ICD10CM
T49.7X2	Poisoning by dental drugs, topically applied, intentional self-harm	ICD10CM
T49.7X2A	Poisoning by dental drugs, topically applied, intentional self-harm, initial encounter	ICD10CM
T49.7X2D	Poisoning by dental drugs, topically applied, intentional self-harm, subsequent encounter	ICD10CM
T49.7X2S	Poisoning by dental drugs, topically applied, intentional self-harm, sequela	ICD10CM
T49.8X2	Poisoning by other topical agents, intentional self-harm	ICD10CM
T49.8X2A	Poisoning by other topical agents, intentional self-harm, initial encounter	ICD10CM
T49.8X2D	Poisoning by other topical agents, intentional self-harm, subsequent encounter	ICD10CM
T49.8X2S	Poisoning by other topical agents, intentional self-harm, sequela	ICD10CM
T49.92	Poisoning by unspecified topical agent, intentional self-harm	ICD10CM

© 2026 Lee H et al. *JAMA Network Open.*

T49.92XA	Poisoning by unspecified topical agent, intentional self-harm, initial encounter	ICD10CM
T49.92XD	Poisoning by unspecified topical agent, intentional self-harm, subsequent encounter	ICD10CM
T49.92XS	Poisoning by unspecified topical agent, intentional self-harm, sequela	ICD10CM
T50.0X2	Poisoning by mineralocorticoids and their antagonists, intentional self-harm	ICD10CM
T50.0X2A	Poisoning by mineralocorticoids and their antagonists, intentional self-harm, initial encounter	ICD10CM
T50.0X2D	Poisoning by mineralocorticoids and their antagonists, intentional self-harm, subsequent encounter	ICD10CM
T50.0X2S	Poisoning by mineralocorticoids and their antagonists, intentional self-harm, sequela	ICD10CM
T50.1X2	Poisoning by loop [high-ceiling] diuretics, intentional self-harm	ICD10CM
T50.1X2A	Poisoning by loop [high-ceiling] diuretics, intentional self-harm, initial encounter	ICD10CM
T50.1X2D	Poisoning by loop [high-ceiling] diuretics, intentional self-harm, subsequent encounter	ICD10CM
T50.1X2S	Poisoning by loop [high-ceiling] diuretics, intentional self-harm, sequela	ICD10CM
T50.2X2	Poisoning by carbonic-anhydrase inhibitors, benzothiadiazides and other diuretics, intentional self-harm	ICD10CM
T50.2X2A	Poisoning by carbonic-anhydrase inhibitors, benzothiadiazides and other diuretics, intentional self-harm, initial encounter	ICD10CM
T50.2X2D	Poisoning by carbonic-anhydrase inhibitors, benzothiadiazides and other diuretics, intentional self-harm, subsequent encounter	ICD10CM
T50.2X2S	Poisoning by carbonic-anhydrase inhibitors, benzothiadiazides and other diuretics, intentional self-harm, sequela	ICD10CM
T50.3X2	Poisoning by electrolytic, caloric and water-balance agents, intentional self-harm	ICD10CM
T50.3X2A	Poisoning by electrolytic, caloric and water-balance agents, intentional self-harm, initial encounter	ICD10CM
T50.3X2D	Poisoning by electrolytic, caloric and water-balance agents, intentional self-harm, subsequent encounter	ICD10CM
T50.3X2S	Poisoning by electrolytic, caloric and water-balance agents, intentional self-harm, sequela	ICD10CM
T50.4X2	Poisoning by drugs affecting uric acid metabolism, intentional self-harm	ICD10CM
T50.4X2A	Poisoning by drugs affecting uric acid metabolism, intentional self-harm, initial encounter	ICD10CM
T50.4X2D	Poisoning by drugs affecting uric acid metabolism, intentional self-harm, subsequent encounter	ICD10CM
T50.4X2S	Poisoning by drugs affecting uric acid metabolism, intentional self-harm, sequela	ICD10CM
T50.5X2	Poisoning by appetite depressants, intentional self-harm	ICD10CM
T50.5X2A	Poisoning by appetite depressants, intentional self-harm, initial encounter	ICD10CM
T50.5X2D	Poisoning by appetite depressants, intentional self-harm, subsequent encounter	ICD10CM
T50.5X2S	Poisoning by appetite depressants, intentional self-harm, sequela	ICD10CM
T50.6X2	Poisoning by antidotes and chelating agents, intentional self-harm	ICD10CM
T50.6X2A	Poisoning by antidotes and chelating agents, intentional self-harm, initial encounter	ICD10CM
T50.6X2D	Poisoning by antidotes and chelating agents, intentional self-harm, subsequent encounter	ICD10CM
T50.6X2S	Poisoning by antidotes and chelating agents, intentional self-harm, sequela	ICD10CM
T50.7X2	Poisoning by analeptics and opioid receptor antagonists, intentional self-harm	ICD10CM
T50.7X2A	Poisoning by analeptics and opioid receptor antagonists, intentional self-harm, initial encounter	ICD10CM
T50.7X2D	Poisoning by analeptics and opioid receptor antagonists, intentional self-harm, subsequent encounter	ICD10CM
T50.7X2S	Poisoning by analeptics and opioid receptor antagonists, intentional self-harm, sequela	ICD10CM
T50.8X2	Poisoning by diagnostic agents, intentional self-harm	ICD10CM
T50.8X2A	Poisoning by diagnostic agents, intentional self-harm, initial encounter	ICD10CM
T50.8X2D	Poisoning by diagnostic agents, intentional self-harm, subsequent encounter	ICD10CM
T50.8X2S	Poisoning by diagnostic agents, intentional self-harm, sequela	ICD10CM

© 2026 Lee H et al. *JAMA Network Open.*

T50.902	Poisoning by unspecified drugs, medicaments and biological substances, intentional self-harm	ICD10CM
T50.902A	Poisoning by unspecified drugs, medicaments and biological substances, intentional self-harm, initial encounter	ICD10CM
T50.902D	Poisoning by unspecified drugs, medicaments and biological substances, intentional self-harm, subsequent encounter	ICD10CM
T50.902S	Poisoning by unspecified drugs, medicaments and biological substances, intentional self-harm, sequela	ICD10CM
T50.992	Poisoning by other drugs, medicaments and biological substances, intentional self-harm	ICD10CM
T50.992A	Poisoning by other drugs, medicaments and biological substances, intentional self-harm, initial encounter	ICD10CM
T50.992D	Poisoning by other drugs, medicaments and biological substances, intentional self-harm, subsequent encounter	ICD10CM
T50.992S	Poisoning by other drugs, medicaments and biological substances, intentional self-harm, sequela	ICD10CM
T50.A12	Poisoning by pertussis vaccine, including combinations with a pertussis component, intentional self-harm	ICD10CM
T50.A12A	Poisoning by pertussis vaccine, including combinations with a pertussis component, intentional self-harm, initial encounter	ICD10CM
T50.A12D	Poisoning by pertussis vaccine, including combinations with a pertussis component, intentional self-harm, subsequent encounter	ICD10CM
T50.A12S	Poisoning by pertussis vaccine, including combinations with a pertussis component, intentional self-harm, sequela	ICD10CM
T50.A22	Poisoning by mixed bacterial vaccines without a pertussis component, intentional self-harm	ICD10CM
T50.A22A	Poisoning by mixed bacterial vaccines without a pertussis component, intentional self-harm, initial encounter	ICD10CM
T50.A22D	Poisoning by mixed bacterial vaccines without a pertussis component, intentional self-harm, subsequent encounter	ICD10CM
T50.A22S	Poisoning by mixed bacterial vaccines without a pertussis component, intentional self-harm, sequela	ICD10CM
T50.A92	Poisoning by other bacterial vaccines, intentional self-harm	ICD10CM
T50.A92A	Poisoning by other bacterial vaccines, intentional self-harm, initial encounter	ICD10CM
T50.A92D	Poisoning by other bacterial vaccines, intentional self-harm, subsequent encounter	ICD10CM
T50.A92S	Poisoning by other bacterial vaccines, intentional self-harm, sequela	ICD10CM
T50.B12	Poisoning by smallpox vaccines, intentional self-harm	ICD10CM
T50.B12A	Poisoning by smallpox vaccines, intentional self-harm, initial encounter	ICD10CM
T50.B12D	Poisoning by smallpox vaccines, intentional self-harm, subsequent encounter	ICD10CM
T50.B12S	Poisoning by smallpox vaccines, intentional self-harm, sequela	ICD10CM
T50.B92	Poisoning by other viral vaccines, intentional self-harm	ICD10CM
T50.B92A	Poisoning by other viral vaccines, intentional self-harm, initial encounter	ICD10CM
T50.B92D	Poisoning by other viral vaccines, intentional self-harm, subsequent encounter	ICD10CM
T50.B92S	Poisoning by other viral vaccines, intentional self-harm, sequela	ICD10CM
T50.Z12	Poisoning by immunoglobulin, intentional self-harm	ICD10CM
T50.Z12A	Poisoning by immunoglobulin, intentional self-harm, initial encounter	ICD10CM
T50.Z12D	Poisoning by immunoglobulin, intentional self-harm, subsequent encounter	ICD10CM
T50.Z12S	Poisoning by immunoglobulin, intentional self-harm, sequela	ICD10CM
T50.Z92	Poisoning by other vaccines and biological substances, intentional self-harm	ICD10CM
T50.Z92A	Poisoning by other vaccines and biological substances, intentional self-harm, initial encounter	ICD10CM
T50.Z92D	Poisoning by other vaccines and biological substances, intentional self-harm, subsequent encounter	ICD10CM
T50.Z92S	Poisoning by other vaccines and biological substances, intentional self-harm, sequela	ICD10CM
T51.0X2	Toxic effect of ethanol, intentional self-harm	ICD10CM

© 2026 Lee H et al. *JAMA Network Open.*

T51.0X2A	Toxic effect of ethanol, intentional self-harm, initial encounter	ICD10CM
T51.0X2D	Toxic effect of ethanol, intentional self-harm, subsequent encounter	ICD10CM
T51.0X2S	Toxic effect of ethanol, intentional self-harm, sequela	ICD10CM
T51.1X2	Toxic effect of methanol, intentional self-harm	ICD10CM
T51.1X2A	Toxic effect of methanol, intentional self-harm, initial encounter	ICD10CM
T51.1X2D	Toxic effect of methanol, intentional self-harm, subsequent encounter	ICD10CM
T51.1X2S	Toxic effect of methanol, intentional self-harm, sequela	ICD10CM
T51.2X2	Toxic effect of 2-Propanol, intentional self-harm	ICD10CM
T51.2X2A	Toxic effect of 2-Propanol, intentional self-harm, initial encounter	ICD10CM
T51.2X2D	Toxic effect of 2-Propanol, intentional self-harm, subsequent encounter	ICD10CM
T51.2X2S	Toxic effect of 2-Propanol, intentional self-harm, sequela	ICD10CM
T51.3X2	Toxic effect of fusel oil, intentional self-harm	ICD10CM
T51.3X2A	Toxic effect of fusel oil, intentional self-harm, initial encounter	ICD10CM
T51.3X2D	Toxic effect of fusel oil, intentional self-harm, subsequent encounter	ICD10CM
T51.3X2S	Toxic effect of fusel oil, intentional self-harm, sequela	ICD10CM
T51.8X2	Toxic effect of other alcohols, intentional self-harm	ICD10CM
T51.8X2A	Toxic effect of other alcohols, intentional self-harm, initial encounter	ICD10CM
T51.8X2D	Toxic effect of other alcohols, intentional self-harm, subsequent encounter	ICD10CM
T51.8X2S	Toxic effect of other alcohols, intentional self-harm, sequela	ICD10CM
T51.92	Toxic effect of unspecified alcohol, intentional self-harm	ICD10CM
T51.92XA	Toxic effect of unspecified alcohol, intentional self-harm, initial encounter	ICD10CM
T51.92XD	Toxic effect of unspecified alcohol, intentional self-harm, subsequent encounter	ICD10CM
T51.92XS	Toxic effect of unspecified alcohol, intentional self-harm, sequela	ICD10CM
T52.0X2	Toxic effect of petroleum products, intentional self-harm	ICD10CM
T52.0X2A	Toxic effect of petroleum products, intentional self-harm, initial encounter	ICD10CM
T52.0X2D	Toxic effect of petroleum products, intentional self-harm, subsequent encounter	ICD10CM
T52.0X2S	Toxic effect of petroleum products, intentional self-harm, sequela	ICD10CM
T52.1X2	Toxic effect of benzene, intentional self-harm	ICD10CM
T52.1X2A	Toxic effect of benzene, intentional self-harm, initial encounter	ICD10CM
T52.1X2D	Toxic effect of benzene, intentional self-harm, subsequent encounter	ICD10CM
T52.1X2S	Toxic effect of benzene, intentional self-harm, sequela	ICD10CM
T52.2X2	Toxic effect of homologues of benzene, intentional self-harm	ICD10CM
T52.2X2A	Toxic effect of homologues of benzene, intentional self-harm, initial encounter	ICD10CM
T52.2X2D	Toxic effect of homologues of benzene, intentional self-harm, subsequent encounter	ICD10CM
T52.2X2S	Toxic effect of homologues of benzene, intentional self-harm, sequela	ICD10CM
T52.3X2	Toxic effect of glycols, intentional self-harm	ICD10CM
T52.3X2A	Toxic effect of glycols, intentional self-harm, initial encounter	ICD10CM
T52.3X2D	Toxic effect of glycols, intentional self-harm, subsequent encounter	ICD10CM
T52.3X2S	Toxic effect of glycols, intentional self-harm, sequela	ICD10CM
T52.4X2	Toxic effect of ketones, intentional self-harm	ICD10CM
T52.4X2A	Toxic effect of ketones, intentional self-harm, initial encounter	ICD10CM
T52.4X2D	Toxic effect of ketones, intentional self-harm, subsequent encounter	ICD10CM

© 2026 Lee H et al. *JAMA Network Open.*

T52.4X2S	Toxic effect of ketones, intentional self-harm, sequela	ICD10CM
T52.8X2	Toxic effect of other organic solvents, intentional self-harm	ICD10CM
T52.8X2A	Toxic effect of other organic solvents, intentional self-harm, initial encounter	ICD10CM
T52.8X2D	Toxic effect of other organic solvents, intentional self-harm, subsequent encounter	ICD10CM
T52.8X2S	Toxic effect of other organic solvents, intentional self-harm, sequela	ICD10CM
T52.92	Toxic effect of unspecified organic solvent, intentional self-harm	ICD10CM
T52.92XA	Toxic effect of unspecified organic solvent, intentional self-harm, initial encounter	ICD10CM
T52.92XD	Toxic effect of unspecified organic solvent, intentional self-harm, subsequent encounter	ICD10CM
T52.92XS	Toxic effect of unspecified organic solvent, intentional self-harm, sequela	ICD10CM
T53.0X2	Toxic effect of carbon tetrachloride, intentional self-harm	ICD10CM
T53.0X2A	Toxic effect of carbon tetrachloride, intentional self-harm, initial encounter	ICD10CM
T53.0X2D	Toxic effect of carbon tetrachloride, intentional self-harm, subsequent encounter	ICD10CM
T53.0X2S	Toxic effect of carbon tetrachloride, intentional self-harm, sequela	ICD10CM
T53.1X2	Toxic effect of chloroform, intentional self-harm	ICD10CM
T53.1X2A	Toxic effect of chloroform, intentional self-harm, initial encounter	ICD10CM
T53.1X2D	Toxic effect of chloroform, intentional self-harm, subsequent encounter	ICD10CM
T53.1X2S	Toxic effect of chloroform, intentional self-harm, sequela	ICD10CM
T53.2X2	Toxic effect of trichloroethylene, intentional self-harm	ICD10CM
T53.2X2A	Toxic effect of trichloroethylene, intentional self-harm, initial encounter	ICD10CM
T53.2X2D	Toxic effect of trichloroethylene, intentional self-harm, subsequent encounter	ICD10CM
T53.2X2S	Toxic effect of trichloroethylene, intentional self-harm, sequela	ICD10CM
T53.3X2	Toxic effect of tetrachloroethylene, intentional self-harm	ICD10CM
T53.3X2A	Toxic effect of tetrachloroethylene, intentional self-harm, initial encounter	ICD10CM
T53.3X2D	Toxic effect of tetrachloroethylene, intentional self-harm, subsequent encounter	ICD10CM
T53.3X2S	Toxic effect of tetrachloroethylene, intentional self-harm, sequela	ICD10CM
T53.4X2	Toxic effect of dichloromethane, intentional self-harm	ICD10CM
T53.4X2A	Toxic effect of dichloromethane, intentional self-harm, initial encounter	ICD10CM
T53.4X2D	Toxic effect of dichloromethane, intentional self-harm, subsequent encounter	ICD10CM
T53.4X2S	Toxic effect of dichloromethane, intentional self-harm, sequela	ICD10CM
T53.5X2	Toxic effect of chlorofluorocarbons, intentional self-harm	ICD10CM
T53.5X2A	Toxic effect of chlorofluorocarbons, intentional self-harm, initial encounter	ICD10CM
T53.5X2D	Toxic effect of chlorofluorocarbons, intentional self-harm, subsequent encounter	ICD10CM
T53.5X2S	Toxic effect of chlorofluorocarbons, intentional self-harm, sequela	ICD10CM
T53.6X2	Toxic effect of other halogen derivatives of aliphatic hydrocarbons, intentional self-harm	ICD10CM
T53.6X2A	Toxic effect of other halogen derivatives of aliphatic hydrocarbons, intentional self-harm, initial encounter	ICD10CM
T53.6X2D	Toxic effect of other halogen derivatives of aliphatic hydrocarbons, intentional self-harm, subsequent encounter	ICD10CM
T53.6X2S	Toxic effect of other halogen derivatives of aliphatic hydrocarbons, intentional self-harm, sequela	ICD10CM
T53.7X2	Toxic effect of other halogen derivatives of aromatic hydrocarbons, intentional self-harm	ICD10CM
T53.7X2A	Toxic effect of other halogen derivatives of aromatic hydrocarbons, intentional self-harm, initial encounter	ICD10CM
T53.7X2D	Toxic effect of other halogen derivatives of aromatic hydrocarbons, intentional self-harm, subsequent encounter	ICD10CM

© 2026 Lee H et al. *JAMA Network Open.*

T53.7X2S	Toxic effect of other halogen derivatives of aromatic hydrocarbons, intentional self-harm, sequela	ICD10CM
T53.92	Toxic effect of unspecified halogen derivatives of aliphatic and aromatic hydrocarbons, intentional self-harm	ICD10CM
T53.92XA	Toxic effect of unspecified halogen derivatives of aliphatic and aromatic hydrocarbons, intentional self-harm, initial encounter	ICD10CM
T53.92XD	Toxic effect of unspecified halogen derivatives of aliphatic and aromatic hydrocarbons, intentional self-harm, subsequent encounter	ICD10CM
T53.92XS	Toxic effect of unspecified halogen derivatives of aliphatic and aromatic hydrocarbons, intentional self-harm, sequela	ICD10CM
T54.0X2	Toxic effect of phenol and phenol homologues, intentional self-harm	ICD10CM
T54.0X2A	Toxic effect of phenol and phenol homologues, intentional self-harm, initial encounter	ICD10CM
T54.0X2D	Toxic effect of phenol and phenol homologues, intentional self-harm, subsequent encounter	ICD10CM
T54.0X2S	Toxic effect of phenol and phenol homologues, intentional self-harm, sequela	ICD10CM
T54.1X2	Toxic effect of other corrosive organic compounds, intentional self-harm	ICD10CM
T54.1X2A	Toxic effect of other corrosive organic compounds, intentional self-harm, initial encounter	ICD10CM
T54.1X2D	Toxic effect of other corrosive organic compounds, intentional self-harm, subsequent encounter	ICD10CM
T54.1X2S	Toxic effect of other corrosive organic compounds, intentional self-harm, sequela	ICD10CM
T54.2X2	Toxic effect of corrosive acids and acid-like substances, intentional self-harm	ICD10CM
T54.2X2A	Toxic effect of corrosive acids and acid-like substances, intentional self-harm, initial encounter	ICD10CM
T54.2X2D	Toxic effect of corrosive acids and acid-like substances, intentional self-harm, subsequent encounter	ICD10CM
T54.2X2S	Toxic effect of corrosive acids and acid-like substances, intentional self-harm, sequela	ICD10CM
T54.3X2	Toxic effect of corrosive alkalis and alkali-like substances, intentional self-harm	ICD10CM
T54.3X2A	Toxic effect of corrosive alkalis and alkali-like substances, intentional self-harm, initial encounter	ICD10CM
T54.3X2D	Toxic effect of corrosive alkalis and alkali-like substances, intentional self-harm, subsequent encounter	ICD10CM
T54.3X2S	Toxic effect of corrosive alkalis and alkali-like substances, intentional self-harm, sequela	ICD10CM
T54.92	Toxic effect of unspecified corrosive substance, intentional self-harm	ICD10CM
T54.92XA	Toxic effect of unspecified corrosive substance, intentional self-harm, initial encounter	ICD10CM
T54.92XD	Toxic effect of unspecified corrosive substance, intentional self-harm, subsequent encounter	ICD10CM
T54.92XS	Toxic effect of unspecified corrosive substance, intentional self-harm, sequela	ICD10CM
T55.0X2	Toxic effect of soaps, intentional self-harm	ICD10CM
T55.0X2A	Toxic effect of soaps, intentional self-harm, initial encounter	ICD10CM
T55.0X2D	Toxic effect of soaps, intentional self-harm, subsequent encounter	ICD10CM
T55.0X2S	Toxic effect of soaps, intentional self-harm, sequela	ICD10CM
T55.1X2	Toxic effect of detergents, intentional self-harm	ICD10CM
T55.1X2A	Toxic effect of detergents, intentional self-harm, initial encounter	ICD10CM
T55.1X2D	Toxic effect of detergents, intentional self-harm, subsequent encounter	ICD10CM
T55.1X2S	Toxic effect of detergents, intentional self-harm, sequela	ICD10CM
T56.0X2	Toxic effect of lead and its compounds, intentional self-harm	ICD10CM
T56.0X2A	Toxic effect of lead and its compounds, intentional self-harm, initial encounter	ICD10CM
T56.0X2D	Toxic effect of lead and its compounds, intentional self-harm, subsequent encounter	ICD10CM
T56.0X2S	Toxic effect of lead and its compounds, intentional self-harm, sequela	ICD10CM
T56.1X2	Toxic effect of mercury and its compounds, intentional self-harm	ICD10CM
T56.1X2A	Toxic effect of mercury and its compounds, intentional self-harm, initial encounter	ICD10CM

© 2026 Lee H et al. *JAMA Network Open.*

T56.1X2D	Toxic effect of mercury and its compounds, intentional self-harm, subsequent encounter	ICD10CM
T56.1X2S	Toxic effect of mercury and its compounds, intentional self-harm, sequela	ICD10CM
T56.2X2	Toxic effect of chromium and its compounds, intentional self-harm	ICD10CM
T56.2X2A	Toxic effect of chromium and its compounds, intentional self-harm, initial encounter	ICD10CM
T56.2X2D	Toxic effect of chromium and its compounds, intentional self-harm, subsequent encounter	ICD10CM
T56.2X2S	Toxic effect of chromium and its compounds, intentional self-harm, sequela	ICD10CM
T56.3X2	Toxic effect of cadmium and its compounds, intentional self-harm	ICD10CM
T56.3X2A	Toxic effect of cadmium and its compounds, intentional self-harm, initial encounter	ICD10CM
T56.3X2D	Toxic effect of cadmium and its compounds, intentional self-harm, subsequent encounter	ICD10CM
T56.3X2S	Toxic effect of cadmium and its compounds, intentional self-harm, sequela	ICD10CM
T56.4X2	Toxic effect of copper and its compounds, intentional self-harm	ICD10CM
T56.4X2A	Toxic effect of copper and its compounds, intentional self-harm, initial encounter	ICD10CM
T56.4X2D	Toxic effect of copper and its compounds, intentional self-harm, subsequent encounter	ICD10CM
T56.4X2S	Toxic effect of copper and its compounds, intentional self-harm, sequela	ICD10CM
T56.5X2	Toxic effect of zinc and its compounds, intentional self-harm	ICD10CM
T56.5X2A	Toxic effect of zinc and its compounds, intentional self-harm, initial encounter	ICD10CM
T56.5X2D	Toxic effect of zinc and its compounds, intentional self-harm, subsequent encounter	ICD10CM
T56.5X2S	Toxic effect of zinc and its compounds, intentional self-harm, sequela	ICD10CM
T56.6X2	Toxic effect of tin and its compounds, intentional self-harm	ICD10CM
T56.6X2A	Toxic effect of tin and its compounds, intentional self-harm, initial encounter	ICD10CM
T56.6X2D	Toxic effect of tin and its compounds, intentional self-harm, subsequent encounter	ICD10CM
T56.6X2S	Toxic effect of tin and its compounds, intentional self-harm, sequela	ICD10CM
T56.7X2	Toxic effect of beryllium and its compounds, intentional self-harm	ICD10CM
T56.7X2A	Toxic effect of beryllium and its compounds, intentional self-harm, initial encounter	ICD10CM
T56.7X2D	Toxic effect of beryllium and its compounds, intentional self-harm, subsequent encounter	ICD10CM
T56.7X2S	Toxic effect of beryllium and its compounds, intentional self-harm, sequela	ICD10CM
T56.812	Toxic effect of thallium, intentional self-harm	ICD10CM
T56.812A	Toxic effect of thallium, intentional self-harm, initial encounter	ICD10CM
T56.812D	Toxic effect of thallium, intentional self-harm, subsequent encounter	ICD10CM
T56.812S	Toxic effect of thallium, intentional self-harm, sequela	ICD10CM
T56.892	Toxic effect of other metals, intentional self-harm	ICD10CM
T56.892A	Toxic effect of other metals, intentional self-harm, initial encounter	ICD10CM
T56.892D	Toxic effect of other metals, intentional self-harm, subsequent encounter	ICD10CM
T56.892S	Toxic effect of other metals, intentional self-harm, sequela	ICD10CM
T56.8x2A	Toxic effect of other metals, intentional self-harm, initial encounter	ICD10CM
T56.8x2D	Toxic effect of other metals, intentional self-harm, subsequent encounter	ICD10CM
T56.8x2S	Toxic effect of other metals, intentional self-harm, sequela	ICD10CM
T56.92	Toxic effect of unspecified metal, intentional self-harm	ICD10CM
T56.92XA	Toxic effect of unspecified metal, intentional self-harm, initial encounter	ICD10CM
T56.92XD	Toxic effect of unspecified metal, intentional self-harm, subsequent encounter	ICD10CM
T56.92XS	Toxic effect of unspecified metal, intentional self-harm, sequela	ICD10CM
T57.0X2	Toxic effect of arsenic and its compounds, intentional self-harm	ICD10CM

© 2026 Lee H et al. *JAMA Network Open.*

T57.0X2A	Toxic effect of arsenic and its compounds, intentional self-harm, initial encounter	ICD10CM
T57.0X2D	Toxic effect of arsenic and its compounds, intentional self-harm, subsequent encounter	ICD10CM
T57.0X2S	Toxic effect of arsenic and its compounds, intentional self-harm, sequela	ICD10CM
T57.1X2	Toxic effect of phosphorus and its compounds, intentional self-harm	ICD10CM
T57.1X2A	Toxic effect of phosphorus and its compounds, intentional self-harm, initial encounter	ICD10CM
T57.1X2D	Toxic effect of phosphorus and its compounds, intentional self-harm, subsequent encounter	ICD10CM
T57.1X2S	Toxic effect of phosphorus and its compounds, intentional self-harm, sequela	ICD10CM
T57.2X2	Toxic effect of manganese and its compounds, intentional self-harm	ICD10CM
T57.2X2A	Toxic effect of manganese and its compounds, intentional self-harm, initial encounter	ICD10CM
T57.2X2D	Toxic effect of manganese and its compounds, intentional self-harm, subsequent encounter	ICD10CM
T57.2X2S	Toxic effect of manganese and its compounds, intentional self-harm, sequela	ICD10CM
T57.3X2	Toxic effect of hydrogen cyanide, intentional self-harm	ICD10CM
T57.3X2A	Toxic effect of hydrogen cyanide, intentional self-harm, initial encounter	ICD10CM
T57.3X2D	Toxic effect of hydrogen cyanide, intentional self-harm, subsequent encounter	ICD10CM
T57.3X2S	Toxic effect of hydrogen cyanide, intentional self-harm, sequela	ICD10CM
T57.8X2	Toxic effect of other specified inorganic substances, intentional self-harm	ICD10CM
T57.8X2A	Toxic effect of other specified inorganic substances, intentional self-harm, initial encounter	ICD10CM
T57.8X2D	Toxic effect of other specified inorganic substances, intentional self-harm, subsequent encounter	ICD10CM
T57.8X2S	Toxic effect of other specified inorganic substances, intentional self-harm, sequela	ICD10CM
T57.92	Toxic effect of unspecified inorganic substance, intentional self-harm	ICD10CM
T57.92XA	Toxic effect of unspecified inorganic substance, intentional self-harm, initial encounter	ICD10CM
T57.92XD	Toxic effect of unspecified inorganic substance, intentional self-harm, subsequent encounter	ICD10CM
T57.92XS	Toxic effect of unspecified inorganic substance, intentional self-harm, sequela	ICD10CM
T58.02	Toxic effect of carbon monoxide from motor vehicle exhaust, intentional self-harm	ICD10CM
T58.02XA	Toxic effect of carbon monoxide from motor vehicle exhaust, intentional self-harm, initial encounter	ICD10CM
T58.02XD	Toxic effect of carbon monoxide from motor vehicle exhaust, intentional self-harm, subsequent encounter	ICD10CM
T58.02XS	Toxic effect of carbon monoxide from motor vehicle exhaust, intentional self-harm, sequela	ICD10CM
T58.12	Toxic effect of carbon monoxide from utility gas, intentional self-harm	ICD10CM
T58.12XA	Toxic effect of carbon monoxide from utility gas, intentional self-harm, initial encounter	ICD10CM
T58.12XD	Toxic effect of carbon monoxide from utility gas, intentional self-harm, subsequent encounter	ICD10CM
T58.12XS	Toxic effect of carbon monoxide from utility gas, intentional self-harm, sequela	ICD10CM
T58.2X2	Toxic effect of carbon monoxide from incomplete combustion of other domestic fuels, intentional self-harm	ICD10CM
T58.2X2A	Toxic effect of carbon monoxide from incomplete combustion of other domestic fuels, intentional self-harm, initial encounter	ICD10CM
T58.2X2D	Toxic effect of carbon monoxide from incomplete combustion of other domestic fuels, intentional self-harm, subsequent encounter	ICD10CM
T58.2X2S	Toxic effect of carbon monoxide from incomplete combustion of other domestic fuels, intentional self-harm, sequela	ICD10CM
T58.8X2	Toxic effect of carbon monoxide from other source, intentional self-harm	ICD10CM
T58.8X2A	Toxic effect of carbon monoxide from other source, intentional self-harm, initial encounter	ICD10CM
T58.8X2D	Toxic effect of carbon monoxide from other source, intentional self-harm, subsequent encounter	ICD10CM
T58.8X2S	Toxic effect of carbon monoxide from other source, intentional self-harm, sequela	ICD10CM
T58.92	Toxic effect of carbon monoxide from unspecified source, intentional self-harm	ICD10CM

© 2026 Lee H et al. *JAMA Network Open.*

T58.92XA	Toxic effect of carbon monoxide from unspecified source, intentional self-harm, initial encounter	ICD10CM
T58.92XD	Toxic effect of carbon monoxide from unspecified source, intentional self-harm, subsequent encounter	ICD10CM
T58.92XS	Toxic effect of carbon monoxide from unspecified source, intentional self-harm, sequela	ICD10CM
T59.0X2	Toxic effect of nitrogen oxides, intentional self-harm	ICD10CM
T59.0X2A	Toxic effect of nitrogen oxides, intentional self-harm, initial encounter	ICD10CM
T59.0X2D	Toxic effect of nitrogen oxides, intentional self-harm, subsequent encounter	ICD10CM
T59.0X2S	Toxic effect of nitrogen oxides, intentional self-harm, sequela	ICD10CM
T59.1X2	Toxic effect of sulfur dioxide, intentional self-harm	ICD10CM
T59.1X2A	Toxic effect of sulfur dioxide, intentional self-harm, initial encounter	ICD10CM
T59.1X2D	Toxic effect of sulfur dioxide, intentional self-harm, subsequent encounter	ICD10CM
T59.1X2S	Toxic effect of sulfur dioxide, intentional self-harm, sequela	ICD10CM
T59.2X2	Toxic effect of formaldehyde, intentional self-harm	ICD10CM
T59.2X2A	Toxic effect of formaldehyde, intentional self-harm, initial encounter	ICD10CM
T59.2X2D	Toxic effect of formaldehyde, intentional self-harm, subsequent encounter	ICD10CM
T59.2X2S	Toxic effect of formaldehyde, intentional self-harm, sequela	ICD10CM
T59.3X2	Toxic effect of lacrimogenic gas, intentional self-harm	ICD10CM
T59.3X2A	Toxic effect of lacrimogenic gas, intentional self-harm, initial encounter	ICD10CM
T59.3X2D	Toxic effect of lacrimogenic gas, intentional self-harm, subsequent encounter	ICD10CM
T59.3X2S	Toxic effect of lacrimogenic gas, intentional self-harm, sequela	ICD10CM
T59.4X2	Toxic effect of chlorine gas, intentional self-harm	ICD10CM
T59.4X2A	Toxic effect of chlorine gas, intentional self-harm, initial encounter	ICD10CM
T59.4X2D	Toxic effect of chlorine gas, intentional self-harm, subsequent encounter	ICD10CM
T59.4X2S	Toxic effect of chlorine gas, intentional self-harm, sequela	ICD10CM
T59.5X2	Toxic effect of fluorine gas and hydrogen fluoride, intentional self-harm	ICD10CM
T59.5X2A	Toxic effect of fluorine gas and hydrogen fluoride, intentional self-harm, initial encounter	ICD10CM
T59.5X2D	Toxic effect of fluorine gas and hydrogen fluoride, intentional self-harm, subsequent encounter	ICD10CM
T59.5X2S	Toxic effect of fluorine gas and hydrogen fluoride, intentional self-harm, sequela	ICD10CM
T59.6X2	Toxic effect of hydrogen sulfide, intentional self-harm	ICD10CM
T59.6X2A	Toxic effect of hydrogen sulfide, intentional self-harm, initial encounter	ICD10CM
T59.6X2D	Toxic effect of hydrogen sulfide, intentional self-harm, subsequent encounter	ICD10CM
T59.6X2S	Toxic effect of hydrogen sulfide, intentional self-harm, sequela	ICD10CM
T59.7X2	Toxic effect of carbon dioxide, intentional self-harm	ICD10CM
T59.7X2A	Toxic effect of carbon dioxide, intentional self-harm, initial encounter	ICD10CM
T59.7X2D	Toxic effect of carbon dioxide, intentional self-harm, subsequent encounter	ICD10CM
T59.7X2S	Toxic effect of carbon dioxide, intentional self-harm, sequela	ICD10CM
T59.812	Toxic effect of smoke, intentional self-harm	ICD10CM
T59.812A	Toxic effect of smoke, intentional self-harm, initial encounter	ICD10CM
T59.812D	Toxic effect of smoke, intentional self-harm, subsequent encounter	ICD10CM
T59.812S	Toxic effect of smoke, intentional self-harm, sequela	ICD10CM
T59.892	Toxic effect of other specified gases, fumes and vapors, intentional self-harm	ICD10CM
T59.892A	Toxic effect of other specified gases, fumes and vapors, intentional self-harm, initial encounter	ICD10CM

© 2026 Lee H et al. *JAMA Network Open.*

T59.892D	Toxic effect of other specified gases, fumes and vapors, intentional self-harm, subsequent encounter	ICD10CM
T59.892S	Toxic effect of other specified gases, fumes and vapors, intentional self-harm, sequela	ICD10CM
T59.92	Toxic effect of unspecified gases, fumes and vapors, intentional self-harm	ICD10CM
T59.92XA	Toxic effect of unspecified gases, fumes and vapors, intentional self-harm, initial encounter	ICD10CM
T59.92XD	Toxic effect of unspecified gases, fumes and vapors, intentional self-harm, subsequent encounter	ICD10CM
T59.92XS	Toxic effect of unspecified gases, fumes and vapors, intentional self-harm, sequela	ICD10CM
T60.0X2	Toxic effect of organophosphate and carbamate insecticides, intentional self-harm	ICD10CM
T60.0X2A	Toxic effect of organophosphate and carbamate insecticides, intentional self-harm, initial encounter	ICD10CM
T60.0X2D	Toxic effect of organophosphate and carbamate insecticides, intentional self-harm, subsequent encounter	ICD10CM
T60.0X2S	Toxic effect of organophosphate and carbamate insecticides, intentional self-harm, sequela	ICD10CM
T60.1X2	Toxic effect of halogenated insecticides, intentional self-harm	ICD10CM
T60.1X2A	Toxic effect of halogenated insecticides, intentional self-harm, initial encounter	ICD10CM
T60.1X2D	Toxic effect of halogenated insecticides, intentional self-harm, subsequent encounter	ICD10CM
T60.1X2S	Toxic effect of halogenated insecticides, intentional self-harm, sequela	ICD10CM
T60.2X2	Toxic effect of other insecticides, intentional self-harm	ICD10CM
T60.2X2A	Toxic effect of other insecticides, intentional self-harm, initial encounter	ICD10CM
T60.2X2D	Toxic effect of other insecticides, intentional self-harm, subsequent encounter	ICD10CM
T60.2X2S	Toxic effect of other insecticides, intentional self-harm, sequela	ICD10CM
T60.3X2	Toxic effect of herbicides and fungicides, intentional self-harm	ICD10CM
T60.3X2A	Toxic effect of herbicides and fungicides, intentional self-harm, initial encounter	ICD10CM
T60.3X2D	Toxic effect of herbicides and fungicides, intentional self-harm, subsequent encounter	ICD10CM
T60.3X2S	Toxic effect of herbicides and fungicides, intentional self-harm, sequela	ICD10CM
T60.4X2	Toxic effect of rodenticides, intentional self-harm	ICD10CM
T60.4X2A	Toxic effect of rodenticides, intentional self-harm, initial encounter	ICD10CM
T60.4X2D	Toxic effect of rodenticides, intentional self-harm, subsequent encounter	ICD10CM
T60.4X2S	Toxic effect of rodenticides, intentional self-harm, sequela	ICD10CM
T60.8X2	Toxic effect of other pesticides, intentional self-harm	ICD10CM
T60.8X2A	Toxic effect of other pesticides, intentional self-harm, initial encounter	ICD10CM
T60.8X2D	Toxic effect of other pesticides, intentional self-harm, subsequent encounter	ICD10CM
T60.8X2S	Toxic effect of other pesticides, intentional self-harm, sequela	ICD10CM
T60.92	Toxic effect of unspecified pesticide, intentional self-harm	ICD10CM
T60.92XA	Toxic effect of unspecified pesticide, intentional self-harm, initial encounter	ICD10CM
T60.92XD	Toxic effect of unspecified pesticide, intentional self-harm, subsequent encounter	ICD10CM
T60.92XS	Toxic effect of unspecified pesticide, intentional self-harm, sequela	ICD10CM
T61.02	Ciguatera fish poisoning, intentional self-harm	ICD10CM
T61.02XA	Ciguatera fish poisoning, intentional self-harm, initial encounter	ICD10CM
T61.02XD	Ciguatera fish poisoning, intentional self-harm, subsequent encounter	ICD10CM
T61.02XS	Ciguatera fish poisoning, intentional self-harm, sequela	ICD10CM
T61.12	Scombroid fish poisoning, intentional self-harm	ICD10CM
T61.12XA	Scombroid fish poisoning, intentional self-harm, initial encounter	ICD10CM
T61.12XD	Scombroid fish poisoning, intentional self-harm, subsequent encounter	ICD10CM

© 2026 Lee H et al. *JAMA Network Open.*

T61.12XS	Scombroid fish poisoning, intentional self-harm, sequela	ICD10CM
T61.772	Other fish poisoning, intentional self-harm	ICD10CM
T61.772A	Other fish poisoning, intentional self-harm, initial encounter	ICD10CM
T61.772D	Other fish poisoning, intentional self-harm, subsequent encounter	ICD10CM
T61.772S	Other fish poisoning, intentional self-harm, sequela	ICD10CM
T61.782	Other shellfish poisoning, intentional self-harm	ICD10CM
T61.782A	Other shellfish poisoning, intentional self-harm, initial encounter	ICD10CM
T61.782D	Other shellfish poisoning, intentional self-harm, subsequent encounter	ICD10CM
T61.782S	Other shellfish poisoning, intentional self-harm, sequela	ICD10CM
T61.8X2	Toxic effect of other seafood, intentional self-harm	ICD10CM
T61.8X2A	Toxic effect of other seafood, intentional self-harm, initial encounter	ICD10CM
T61.8X2D	Toxic effect of other seafood, intentional self-harm, subsequent encounter	ICD10CM
T61.8X2S	Toxic effect of other seafood, intentional self-harm, sequela	ICD10CM
T61.92	Toxic effect of unspecified seafood, intentional self-harm	ICD10CM
T61.92XA	Toxic effect of unspecified seafood, intentional self-harm, initial encounter	ICD10CM
T61.92XD	Toxic effect of unspecified seafood, intentional self-harm, subsequent encounter	ICD10CM
T61.92XS	Toxic effect of unspecified seafood, intentional self-harm, sequela	ICD10CM
T62.0X2	Toxic effect of ingested mushrooms, intentional self-harm	ICD10CM
T62.0X2A	Toxic effect of ingested mushrooms, intentional self-harm, initial encounter	ICD10CM
T62.0X2D	Toxic effect of ingested mushrooms, intentional self-harm, subsequent encounter	ICD10CM
T62.0X2S	Toxic effect of ingested mushrooms, intentional self-harm, sequela	ICD10CM
T62.1X2	Toxic effect of ingested berries, intentional self-harm	ICD10CM
T62.1X2A	Toxic effect of ingested berries, intentional self-harm, initial encounter	ICD10CM
T62.1X2D	Toxic effect of ingested berries, intentional self-harm, subsequent encounter	ICD10CM
T62.1X2S	Toxic effect of ingested berries, intentional self-harm, sequela	ICD10CM
T62.2X2	Toxic effect of other ingested (parts of) plant(s), intentional self-harm	ICD10CM
T62.2X2A	Toxic effect of other ingested (parts of) plant(s), intentional self-harm, initial encounter	ICD10CM
T62.2X2D	Toxic effect of other ingested (parts of) plant(s), intentional self-harm, subsequent encounter	ICD10CM
T62.2X2S	Toxic effect of other ingested (parts of) plant(s), intentional self-harm, sequela	ICD10CM
T62.8X2	Toxic effect of other specified noxious substances eaten as food, intentional self-harm	ICD10CM
T62.8X2A	Toxic effect of other specified noxious substances eaten as food, intentional self-harm, initial encounter	ICD10CM
T62.8X2D	Toxic effect of other specified noxious substances eaten as food, intentional self-harm, subsequent encounter	ICD10CM
T62.8X2S	Toxic effect of other specified noxious substances eaten as food, intentional self-harm, sequela	ICD10CM
T62.92	Toxic effect of unspecified noxious substance eaten as food, intentional self-harm	ICD10CM
T62.92XA	Toxic effect of unspecified noxious substance eaten as food, intentional self-harm, initial encounter	ICD10CM
T62.92XD	Toxic effect of unspecified noxious substance eaten as food, intentional self-harm, subsequent encounter	ICD10CM
T62.92XS	Toxic effect of unspecified noxious substance eaten as food, intentional self-harm, sequela	ICD10CM
T63.002	Toxic effect of unspecified snake venom, intentional self-harm	ICD10CM
T63.002A	Toxic effect of unspecified snake venom, intentional self-harm, initial encounter	ICD10CM
T63.002D	Toxic effect of unspecified snake venom, intentional self-harm, subsequent encounter	ICD10CM
T63.002S	Toxic effect of unspecified snake venom, intentional self-harm, sequela	ICD10CM

© 2026 Lee H et al. *JAMA Network Open.*

T63.012	Toxic effect of rattlesnake venom, intentional self-harm	ICD10CM
T63.012A	Toxic effect of rattlesnake venom, intentional self-harm, initial encounter	ICD10CM
T63.012D	Toxic effect of rattlesnake venom, intentional self-harm, subsequent encounter	ICD10CM
T63.012S	Toxic effect of rattlesnake venom, intentional self-harm, sequela	ICD10CM
T63.022	Toxic effect of coral snake venom, intentional self-harm	ICD10CM
T63.022A	Toxic effect of coral snake venom, intentional self-harm, initial encounter	ICD10CM
T63.022D	Toxic effect of coral snake venom, intentional self-harm, subsequent encounter	ICD10CM
T63.022S	Toxic effect of coral snake venom, intentional self-harm, sequela	ICD10CM
T63.032	Toxic effect of taipan venom, intentional self-harm	ICD10CM
T63.032A	Toxic effect of taipan venom, intentional self-harm, initial encounter	ICD10CM
T63.032D	Toxic effect of taipan venom, intentional self-harm, subsequent encounter	ICD10CM
T63.032S	Toxic effect of taipan venom, intentional self-harm, sequela	ICD10CM
T63.042	Toxic effect of cobra venom, intentional self-harm	ICD10CM
T63.042A	Toxic effect of cobra venom, intentional self-harm, initial encounter	ICD10CM
T63.042D	Toxic effect of cobra venom, intentional self-harm, subsequent encounter	ICD10CM
T63.042S	Toxic effect of cobra venom, intentional self-harm, sequela	ICD10CM
T63.062	Toxic effect of venom of other North and South American snake, intentional self-harm	ICD10CM
T63.062A	Toxic effect of venom of other North and South American snake, intentional self-harm, initial encounter	ICD10CM
T63.062D	Toxic effect of venom of other North and South American snake, intentional self-harm, subsequent encounter	ICD10CM
T63.062S	Toxic effect of venom of other North and South American snake, intentional self-harm, sequela	ICD10CM
T63.072	Toxic effect of venom of other Australian snake, intentional self-harm	ICD10CM
T63.072A	Toxic effect of venom of other Australian snake, intentional self-harm, initial encounter	ICD10CM
T63.072D	Toxic effect of venom of other Australian snake, intentional self-harm, subsequent encounter	ICD10CM
T63.072S	Toxic effect of venom of other Australian snake, intentional self-harm, sequela	ICD10CM
T63.082	Toxic effect of venom of other African and Asian snake, intentional self-harm	ICD10CM
T63.082A	Toxic effect of venom of other African and Asian snake, intentional self-harm, initial encounter	ICD10CM
T63.082D	Toxic effect of venom of other African and Asian snake, intentional self-harm, subsequent encounter	ICD10CM
T63.082S	Toxic effect of venom of other African and Asian snake, intentional self-harm, sequela	ICD10CM
T63.092	Toxic effect of venom of other snake, intentional self-harm	ICD10CM
T63.092A	Toxic effect of venom of other snake, intentional self-harm, initial encounter	ICD10CM
T63.092D	Toxic effect of venom of other snake, intentional self-harm, subsequent encounter	ICD10CM
T63.092S	Toxic effect of venom of other snake, intentional self-harm, sequela	ICD10CM
T63.112	Toxic effect of venom of gila monster, intentional self-harm	ICD10CM
T63.112A	Toxic effect of venom of gila monster, intentional self-harm, initial encounter	ICD10CM
T63.112D	Toxic effect of venom of gila monster, intentional self-harm, subsequent encounter	ICD10CM
T63.112S	Toxic effect of venom of gila monster, intentional self-harm, sequela	ICD10CM
T63.122	Toxic effect of venom of other venomous lizard, intentional self-harm	ICD10CM
T63.122A	Toxic effect of venom of other venomous lizard, intentional self-harm, initial encounter	ICD10CM
T63.122D	Toxic effect of venom of other venomous lizard, intentional self-harm, subsequent encounter	ICD10CM
T63.122S	Toxic effect of venom of other venomous lizard, intentional self-harm, sequela	ICD10CM
T63.192	Toxic effect of venom of other reptiles, intentional self-harm	ICD10CM

© 2026 Lee H et al. *JAMA Network Open.*

T63.192A	Toxic effect of venom of other reptiles, intentional self-harm, initial encounter	ICD10CM
T63.192D	Toxic effect of venom of other reptiles, intentional self-harm, subsequent encounter	ICD10CM
T63.192S	Toxic effect of venom of other reptiles, intentional self-harm, sequela	ICD10CM
T63.2X2	Toxic effect of venom of scorpion, intentional self-harm	ICD10CM
T63.2X2A	Toxic effect of venom of scorpion, intentional self-harm, initial encounter	ICD10CM
T63.2X2D	Toxic effect of venom of scorpion, intentional self-harm, subsequent encounter	ICD10CM
T63.2X2S	Toxic effect of venom of scorpion, intentional self-harm, sequela	ICD10CM
T63.302	Toxic effect of unspecified spider venom, intentional self-harm	ICD10CM
T63.302A	Toxic effect of unspecified spider venom, intentional self-harm, initial encounter	ICD10CM
T63.302D	Toxic effect of unspecified spider venom, intentional self-harm, subsequent encounter	ICD10CM
T63.302S	Toxic effect of unspecified spider venom, intentional self-harm, sequela	ICD10CM
T63.312	Toxic effect of venom of black widow spider, intentional self-harm	ICD10CM
T63.312A	Toxic effect of venom of black widow spider, intentional self-harm, initial encounter	ICD10CM
T63.312D	Toxic effect of venom of black widow spider, intentional self-harm, subsequent encounter	ICD10CM
T63.312S	Toxic effect of venom of black widow spider, intentional self-harm, sequela	ICD10CM
T63.322	Toxic effect of venom of tarantula, intentional self-harm	ICD10CM
T63.322A	Toxic effect of venom of tarantula, intentional self-harm, initial encounter	ICD10CM
T63.322D	Toxic effect of venom of tarantula, intentional self-harm, subsequent encounter	ICD10CM
T63.322S	Toxic effect of venom of tarantula, intentional self-harm, sequela	ICD10CM
T63.332	Toxic effect of venom of brown recluse spider, intentional self-harm	ICD10CM
T63.332A	Toxic effect of venom of brown recluse spider, intentional self-harm, initial encounter	ICD10CM
T63.332D	Toxic effect of venom of brown recluse spider, intentional self-harm, subsequent encounter	ICD10CM
T63.332S	Toxic effect of venom of brown recluse spider, intentional self-harm, sequela	ICD10CM
T63.392	Toxic effect of venom of other spider, intentional self-harm	ICD10CM
T63.392A	Toxic effect of venom of other spider, intentional self-harm, initial encounter	ICD10CM
T63.392D	Toxic effect of venom of other spider, intentional self-harm, subsequent encounter	ICD10CM
T63.392S	Toxic effect of venom of other spider, intentional self-harm, sequela	ICD10CM
T63.412	Toxic effect of venom of centipedes and venomous millipedes, intentional self-harm	ICD10CM
T63.412A	Toxic effect of venom of centipedes and venomous millipedes, intentional self-harm, initial encounter	ICD10CM
T63.412D	Toxic effect of venom of centipedes and venomous millipedes, intentional self-harm, subsequent encounter	ICD10CM
T63.412S	Toxic effect of venom of centipedes and venomous millipedes, intentional self-harm, sequela	ICD10CM
T63.422	Toxic effect of venom of ants, intentional self-harm	ICD10CM
T63.422A	Toxic effect of venom of ants, intentional self-harm, initial encounter	ICD10CM
T63.422D	Toxic effect of venom of ants, intentional self-harm, subsequent encounter	ICD10CM
T63.422S	Toxic effect of venom of ants, intentional self-harm, sequela	ICD10CM
T63.432	Toxic effect of venom of caterpillars, intentional self-harm	ICD10CM
T63.432A	Toxic effect of venom of caterpillars, intentional self-harm, initial encounter	ICD10CM
T63.432D	Toxic effect of venom of caterpillars, intentional self-harm, subsequent encounter	ICD10CM
T63.432S	Toxic effect of venom of caterpillars, intentional self-harm, sequela	ICD10CM
T63.442	Toxic effect of venom of bees, intentional self-harm	ICD10CM
T63.442A	Toxic effect of venom of bees, intentional self-harm, initial encounter	ICD10CM

© 2026 Lee H et al. *JAMA Network Open.*

T63.442D	Toxic effect of venom of bees, intentional self-harm, subsequent encounter	ICD10CM
T63.442S	Toxic effect of venom of bees, intentional self-harm, sequela	ICD10CM
T63.452	Toxic effect of venom of hornets, intentional self-harm	ICD10CM
T63.452A	Toxic effect of venom of hornets, intentional self-harm, initial encounter	ICD10CM
T63.452D	Toxic effect of venom of hornets, intentional self-harm, subsequent encounter	ICD10CM
T63.452S	Toxic effect of venom of hornets, intentional self-harm, sequela	ICD10CM
T63.462	Toxic effect of venom of wasps, intentional self-harm	ICD10CM
T63.462A	Toxic effect of venom of wasps, intentional self-harm, initial encounter	ICD10CM
T63.462D	Toxic effect of venom of wasps, intentional self-harm, subsequent encounter	ICD10CM
T63.462S	Toxic effect of venom of wasps, intentional self-harm, sequela	ICD10CM
T63.482	Toxic effect of venom of other arthropod, intentional self-harm	ICD10CM
T63.482A	Toxic effect of venom of other arthropod, intentional self-harm, initial encounter	ICD10CM
T63.482D	Toxic effect of venom of other arthropod, intentional self-harm, subsequent encounter	ICD10CM
T63.482S	Toxic effect of venom of other arthropod, intentional self-harm, sequela	ICD10CM
T63.512	Toxic effect of contact with stingray, intentional self-harm	ICD10CM
T63.512A	Toxic effect of contact with stingray, intentional self-harm, initial encounter	ICD10CM
T63.512D	Toxic effect of contact with stingray, intentional self-harm, subsequent encounter	ICD10CM
T63.512S	Toxic effect of contact with stingray, intentional self-harm, sequela	ICD10CM
T63.592	Toxic effect of contact with other venomous fish, intentional self-harm	ICD10CM
T63.592A	Toxic effect of contact with other venomous fish, intentional self-harm, initial encounter	ICD10CM
T63.592D	Toxic effect of contact with other venomous fish, intentional self-harm, subsequent encounter	ICD10CM
T63.592S	Toxic effect of contact with other venomous fish, intentional self-harm, sequela	ICD10CM
T63.612	Toxic effect of contact with Portugese Man-o-war, intentional self-harm	ICD10CM
T63.612A	Toxic effect of contact with Portugese Man-o-war, intentional self-harm, initial encounter	ICD10CM
T63.612D	Toxic effect of contact with Portugese Man-o-war, intentional self-harm, subsequent encounter	ICD10CM
T63.612S	Toxic effect of contact with Portugese Man-o-war, intentional self-harm, sequela	ICD10CM
T63.622	Toxic effect of contact with other jellyfish, intentional self-harm	ICD10CM
T63.622A	Toxic effect of contact with other jellyfish, intentional self-harm, initial encounter	ICD10CM
T63.622D	Toxic effect of contact with other jellyfish, intentional self-harm, subsequent encounter	ICD10CM
T63.622S	Toxic effect of contact with other jellyfish, intentional self-harm, sequela	ICD10CM
T63.632	Toxic effect of contact with sea anemone, intentional self-harm	ICD10CM
T63.632A	Toxic effect of contact with sea anemone, intentional self-harm, initial encounter	ICD10CM
T63.632D	Toxic effect of contact with sea anemone, intentional self-harm, subsequent encounter	ICD10CM
T63.632S	Toxic effect of contact with sea anemone, intentional self-harm, sequela	ICD10CM
T63.692	Toxic effect of contact with other venomous marine animals, intentional self-harm	ICD10CM
T63.692A	Toxic effect of contact with other venomous marine animals, intentional self-harm, initial encounter	ICD10CM
T63.692D	Toxic effect of contact with other venomous marine animals, intentional self-harm, subsequent encounter	ICD10CM
T63.692S	Toxic effect of contact with other venomous marine animals, intentional self-harm, sequela	ICD10CM
T63.712	Toxic effect of contact with venomous marine plant, intentional self-harm	ICD10CM
T63.712A	Toxic effect of contact with venomous marine plant, intentional self-harm, initial encounter	ICD10CM
T63.712D	Toxic effect of contact with venomous marine plant, intentional self-harm, subsequent encounter	ICD10CM

© 2026 Lee H et al. *JAMA Network Open.*

T63.712S	Toxic effect of contact with venomous marine plant, intentional self-harm, sequela	ICD10CM
T63.792	Toxic effect of contact with other venomous plant, intentional self-harm	ICD10CM
T63.792A	Toxic effect of contact with other venomous plant, intentional self-harm, initial encounter	ICD10CM
T63.792D	Toxic effect of contact with other venomous plant, intentional self-harm, subsequent encounter	ICD10CM
T63.792S	Toxic effect of contact with other venomous plant, intentional self-harm, sequela	ICD10CM
T63.812	Toxic effect of contact with venomous frog, intentional self-harm	ICD10CM
T63.812A	Toxic effect of contact with venomous frog, intentional self-harm, initial encounter	ICD10CM
T63.812D	Toxic effect of contact with venomous frog, intentional self-harm, subsequent encounter	ICD10CM
T63.812S	Toxic effect of contact with venomous frog, intentional self-harm, sequela	ICD10CM
T63.822	Toxic effect of contact with venomous toad, intentional self-harm	ICD10CM
T63.822A	Toxic effect of contact with venomous toad, intentional self-harm, initial encounter	ICD10CM
T63.822D	Toxic effect of contact with venomous toad, intentional self-harm, subsequent encounter	ICD10CM
T63.822S	Toxic effect of contact with venomous toad, intentional self-harm, sequela	ICD10CM
T63.832	Toxic effect of contact with other venomous amphibian, intentional self-harm	ICD10CM
T63.832A	Toxic effect of contact with other venomous amphibian, intentional self-harm, initial encounter	ICD10CM
T63.832D	Toxic effect of contact with other venomous amphibian, intentional self-harm, subsequent encounter	ICD10CM
T63.832S	Toxic effect of contact with other venomous amphibian, intentional self-harm, sequela	ICD10CM
T63.892	Toxic effect of contact with other venomous animals, intentional self-harm	ICD10CM
T63.892A	Toxic effect of contact with other venomous animals, intentional self-harm, initial encounter	ICD10CM
T63.892D	Toxic effect of contact with other venomous animals, intentional self-harm, subsequent encounter	ICD10CM
T63.892S	Toxic effect of contact with other venomous animals, intentional self-harm, sequela	ICD10CM
T63.92	Toxic effect of contact with unspecified venomous animal, intentional self-harm	ICD10CM
T63.92XA	Toxic effect of contact with unspecified venomous animal, intentional self-harm, initial encounter	ICD10CM
T63.92XD	Toxic effect of contact with unspecified venomous animal, intentional self-harm, subsequent encounter	ICD10CM
T63.92XS	Toxic effect of contact with unspecified venomous animal, intentional self-harm, sequela	ICD10CM
T64.02	Toxic effect of aflatoxin, intentional self-harm	ICD10CM
T64.02XA	Toxic effect of aflatoxin, intentional self-harm, initial encounter	ICD10CM
T64.02XD	Toxic effect of aflatoxin, intentional self-harm, subsequent encounter	ICD10CM
T64.02XS	Toxic effect of aflatoxin, intentional self-harm, sequela	ICD10CM
T64.82	Toxic effect of other mycotoxin food contaminants, intentional self-harm	ICD10CM
T64.82XA	Toxic effect of other mycotoxin food contaminants, intentional self-harm, initial encounter	ICD10CM
T64.82XD	Toxic effect of other mycotoxin food contaminants, intentional self-harm, subsequent encounter	ICD10CM
T64.82XS	Toxic effect of other mycotoxin food contaminants, intentional self-harm, sequela	ICD10CM
T65.0X2	Toxic effect of cyanides, intentional self-harm	ICD10CM
T65.0X2A	Toxic effect of cyanides, intentional self-harm, initial encounter	ICD10CM
T65.0X2D	Toxic effect of cyanides, intentional self-harm, subsequent encounter	ICD10CM
T65.0X2S	Toxic effect of cyanides, intentional self-harm, sequela	ICD10CM
T65.1X2	Toxic effect of strychnine and its salts, intentional self-harm	ICD10CM
T65.1X2A	Toxic effect of strychnine and its salts, intentional self-harm, initial encounter	ICD10CM
T65.1X2D	Toxic effect of strychnine and its salts, intentional self-harm, subsequent encounter	ICD10CM
T65.1X2S	Toxic effect of strychnine and its salts, intentional self-harm, sequela	ICD10CM

© 2026 Lee H et al. *JAMA Network Open.*

T65.212	Toxic effect of chewing tobacco, intentional self-harm	ICD10CM
T65.212A	Toxic effect of chewing tobacco, intentional self-harm, initial encounter	ICD10CM
T65.212D	Toxic effect of chewing tobacco, intentional self-harm, subsequent encounter	ICD10CM
T65.212S	Toxic effect of chewing tobacco, intentional self-harm, sequela	ICD10CM
T65.222	Toxic effect of tobacco cigarettes, intentional self-harm	ICD10CM
T65.222A	Toxic effect of tobacco cigarettes, intentional self-harm, initial encounter	ICD10CM
T65.222D	Toxic effect of tobacco cigarettes, intentional self-harm, subsequent encounter	ICD10CM
T65.222S	Toxic effect of tobacco cigarettes, intentional self-harm, sequela	ICD10CM
T65.292	Toxic effect of other tobacco and nicotine, intentional self-harm	ICD10CM
T65.292A	Toxic effect of other tobacco and nicotine, intentional self-harm, initial encounter	ICD10CM
T65.292D	Toxic effect of other tobacco and nicotine, intentional self-harm, subsequent encounter	ICD10CM
T65.292S	Toxic effect of other tobacco and nicotine, intentional self-harm, sequela	ICD10CM
T65.3X2	Toxic effect of nitroderivatives and aminoderivatives of benzene and its homologues, intentional self-harm	ICD10CM
T65.3X2A	Toxic effect of nitroderivatives and aminoderivatives of benzene and its homologues, intentional self-harm, initial encounter	ICD10CM
T65.3X2D	Toxic effect of nitroderivatives and aminoderivatives of benzene and its homologues, intentional self-harm, subsequent encounter	ICD10CM
T65.3X2S	Toxic effect of nitroderivatives and aminoderivatives of benzene and its homologues, intentional self-harm, sequela	ICD10CM
T65.4X2	Toxic effect of carbon disulfide, intentional self-harm	ICD10CM
T65.4X2A	Toxic effect of carbon disulfide, intentional self-harm, initial encounter	ICD10CM
T65.4X2D	Toxic effect of carbon disulfide, intentional self-harm, subsequent encounter	ICD10CM
T65.4X2S	Toxic effect of carbon disulfide, intentional self-harm, sequela	ICD10CM
T65.5X2	Toxic effect of nitroglycerin and other nitric acids and esters, intentional self-harm	ICD10CM
T65.5X2A	Toxic effect of nitroglycerin and other nitric acids and esters, intentional self-harm, initial encounter	ICD10CM
T65.5X2D	Toxic effect of nitroglycerin and other nitric acids and esters, intentional self-harm, subsequent encounter	ICD10CM
T65.5X2S	Toxic effect of nitroglycerin and other nitric acids and esters, intentional self-harm, sequela	ICD10CM
T65.6X2	Toxic effect of paints and dyes, not elsewhere classified, intentional self-harm	ICD10CM
T65.6X2A	Toxic effect of paints and dyes, not elsewhere classified, intentional self-harm, initial encounter	ICD10CM
T65.6X2D	Toxic effect of paints and dyes, not elsewhere classified, intentional self-harm, subsequent encounter	ICD10CM
T65.6X2S	Toxic effect of paints and dyes, not elsewhere classified, intentional self-harm, sequela	ICD10CM
T65.812	Toxic effect of latex, intentional self-harm	ICD10CM
T65.812A	Toxic effect of latex, intentional self-harm, initial encounter	ICD10CM
T65.812D	Toxic effect of latex, intentional self-harm, subsequent encounter	ICD10CM
T65.812S	Toxic effect of latex, intentional self-harm, sequela	ICD10CM
T65.822	Toxic effect of harmful algae and algae toxins, intentional self-harm	ICD10CM
T65.822A	Toxic effect of harmful algae and algae toxins, intentional self-harm, initial encounter	ICD10CM
T65.822D	Toxic effect of harmful algae and algae toxins, intentional self-harm, subsequent encounter	ICD10CM
T65.822S	Toxic effect of harmful algae and algae toxins, intentional self-harm, sequela	ICD10CM
T65.832	Toxic effect of fiberglass, intentional self-harm	ICD10CM
T65.832A	Toxic effect of fiberglass, intentional self-harm, initial encounter	ICD10CM
T65.832D	Toxic effect of fiberglass, intentional self-harm, subsequent encounter	ICD10CM

© 2026 Lee H et al. *JAMA Network Open.*

T65.832S	Toxic effect of fiberglass, intentional self-harm, sequela	ICD10CM
T65.892	Toxic effect of other specified substances, intentional self-harm	ICD10CM
T65.892A	Toxic effect of other specified substances, intentional self-harm, initial encounter	ICD10CM
T65.892D	Toxic effect of other specified substances, intentional self-harm, subsequent encounter	ICD10CM
T65.892S	Toxic effect of other specified substances, intentional self-harm, sequela	ICD10CM
T65.92	Toxic effect of unspecified substance, intentional self-harm	ICD10CM
T65.92XA	Toxic effect of unspecified substance, intentional self-harm, initial encounter	ICD10CM
T65.92XD	Toxic effect of unspecified substance, intentional self-harm, subsequent encounter	ICD10CM
T65.92XS	Toxic effect of unspecified substance, intentional self-harm, sequela	ICD10CM
T71.112	Asphyxiation due to smothering under pillow, intentional self-harm	ICD10CM
T71.112A	Asphyxiation due to smothering under pillow, intentional self-harm, initial encounter	ICD10CM
T71.112D	Asphyxiation due to smothering under pillow, intentional self-harm, subsequent encounter	ICD10CM
T71.112S	Asphyxiation due to smothering under pillow, intentional self-harm, sequela	ICD10CM
T71.122	Asphyxiation due to plastic bag, intentional self-harm	ICD10CM
T71.122A	Asphyxiation due to plastic bag, intentional self-harm, initial encounter	ICD10CM
T71.122D	Asphyxiation due to plastic bag, intentional self-harm, subsequent encounter	ICD10CM
T71.122S	Asphyxiation due to plastic bag, intentional self-harm, sequela	ICD10CM
T71.132	Asphyxiation due to being trapped in bed linens, intentional self-harm	ICD10CM
T71.132A	Asphyxiation due to being trapped in bed linens, intentional self-harm, initial encounter	ICD10CM
T71.132D	Asphyxiation due to being trapped in bed linens, intentional self-harm, subsequent encounter	ICD10CM
T71.132S	Asphyxiation due to being trapped in bed linens, intentional self-harm, sequela	ICD10CM
T71.152	Asphyxiation due to smothering in furniture, intentional self-harm	ICD10CM
T71.152A	Asphyxiation due to smothering in furniture, intentional self-harm, initial encounter	ICD10CM
T71.152D	Asphyxiation due to smothering in furniture, intentional self-harm, subsequent encounter	ICD10CM
T71.152S	Asphyxiation due to smothering in furniture, intentional self-harm, sequela	ICD10CM
T71.162	Asphyxiation due to hanging, intentional self-harm	ICD10CM
T71.162A	Asphyxiation due to hanging, intentional self-harm, initial encounter	ICD10CM
T71.162D	Asphyxiation due to hanging, intentional self-harm, subsequent encounter	ICD10CM
T71.162S	Asphyxiation due to hanging, intentional self-harm, sequela	ICD10CM
T71.192	Asphyxiation due to mechanical threat to breathing due to other causes, intentional self-harm	ICD10CM
T71.192A	Asphyxiation due to mechanical threat to breathing due to other causes, intentional self-harm, initial encounter	ICD10CM
T71.192D	Asphyxiation due to mechanical threat to breathing due to other causes, intentional self-harm, subsequent encounter	ICD10CM
T71.192S	Asphyxiation due to mechanical threat to breathing due to other causes, intentional self-harm, sequela	ICD10CM
T71.222	Asphyxiation due to being trapped in a car trunk, intentional self-harm	ICD10CM
T71.222A	Asphyxiation due to being trapped in a car trunk, intentional self-harm, initial encounter	ICD10CM
T71.222D	Asphyxiation due to being trapped in a car trunk, intentional self-harm, subsequent encounter	ICD10CM
T71.222S	Asphyxiation due to being trapped in a car trunk, intentional self-harm, sequela	ICD10CM
T71.232	Asphyxiation due to being trapped in a (discarded) refrigerator, intentional self-harm	ICD10CM
T71.232A	Asphyxiation due to being trapped in a (discarded) refrigerator, intentional self-harm, initial encounter	ICD10CM
T71.232D	Asphyxiation due to being trapped in a (discarded) refrigerator, intentional self-harm, subsequent encounter	ICD10CM

© 2026 Lee H et al. *JAMA Network Open.*

T71.232S	Asphyxiation due to being trapped in a (discarded) refrigerator, intentional self-harm, sequela	ICD10CM
X71	Intentional self-harm by drowning and submersion	ICD10CM
X71.0	Intentional self-harm by drowning and submersion while in bathtub	ICD10CM
X71.0XXA	Intentional self-harm by drowning and submersion while in bathtub, initial encounter	ICD10CM
X71.0XXD	Intentional self-harm by drowning and submersion while in bathtub, subsequent encounter	ICD10CM
X71.0XXS	Intentional self-harm by drowning and submersion while in bathtub, sequela	ICD10CM
X71.1	Intentional self-harm by drowning and submersion while in swimming pool	ICD10CM
X71.1XXA	Intentional self-harm by drowning and submersion while in swimming pool, initial encounter	ICD10CM
X71.1XXD	Intentional self-harm by drowning and submersion while in swimming pool, subsequent encounter	ICD10CM
X71.1XXS	Intentional self-harm by drowning and submersion while in swimming pool, sequela	ICD10CM
X71.2	Intentional self-harm by drowning and submersion after jump into swimming pool	ICD10CM
X71.2XXA	Intentional self-harm by drowning and submersion after jump into swimming pool, initial encounter	ICD10CM
X71.2XXD	Intentional self-harm by drowning and submersion after jump into swimming pool, subsequent encounter	ICD10CM
X71.2XXS	Intentional self-harm by drowning and submersion after jump into swimming pool, sequela	ICD10CM
X71.3	Intentional self-harm by drowning and submersion in natural water	ICD10CM
X71.3XXA	Intentional self-harm by drowning and submersion in natural water, initial encounter	ICD10CM
X71.3XXD	Intentional self-harm by drowning and submersion in natural water, subsequent encounter	ICD10CM
X71.3XXS	Intentional self-harm by drowning and submersion in natural water, sequela	ICD10CM
X71.8	Other intentional self-harm by drowning and submersion	ICD10CM
X71.8XXA	Other intentional self-harm by drowning and submersion, initial encounter	ICD10CM
X71.8XXD	Other intentional self-harm by drowning and submersion, subsequent encounter	ICD10CM
X71.8XXS	Other intentional self-harm by drowning and submersion, sequela	ICD10CM
X71.9	Intentional self-harm by drowning and submersion, unspecified	ICD10CM
X71.9XXA	Intentional self-harm by drowning and submersion, unspecified, initial encounter	ICD10CM
X71.9XXD	Intentional self-harm by drowning and submersion, unspecified, subsequent encounter	ICD10CM
X71.9XXS	Intentional self-harm by drowning and submersion, unspecified, sequela	ICD10CM
X72	Intentional self-harm by handgun discharge	ICD10CM
X72.XXXA	Intentional self-harm by handgun discharge, initial encounter	ICD10CM
X72.XXXD	Intentional self-harm by handgun discharge, subsequent encounter	ICD10CM
X72.XXXS	Intentional self-harm by handgun discharge, sequela	ICD10CM
X73	Intentional self-harm by rifle, shotgun and larger firearm discharge	ICD10CM
X73.0	Intentional self-harm by shotgun discharge	ICD10CM
X73.0XXA	Intentional self-harm by shotgun discharge, initial encounter	ICD10CM
X73.0XXD	Intentional self-harm by shotgun discharge, subsequent encounter	ICD10CM
X73.0XXS	Intentional self-harm by shotgun discharge, sequela	ICD10CM
X73.1	Intentional self-harm by hunting rifle discharge	ICD10CM
X73.1XXA	Intentional self-harm by hunting rifle discharge, initial encounter	ICD10CM
X73.1XXD	Intentional self-harm by hunting rifle discharge, subsequent encounter	ICD10CM
X73.1XXS	Intentional self-harm by hunting rifle discharge, sequela	ICD10CM
X73.2	Intentional self-harm by machine gun discharge	ICD10CM
X73.2XXA	Intentional self-harm by machine gun discharge, initial encounter	ICD10CM

© 2026 Lee H et al. *JAMA Network Open.*

X73.2XXD	Intentional self-harm by machine gun discharge, subsequent encounter	ICD10CM
X73.2XXS	Intentional self-harm by machine gun discharge, sequela	ICD10CM
X73.8	Intentional self-harm by other larger firearm discharge	ICD10CM
X73.8XXA	Intentional self-harm by other larger firearm discharge, initial encounter	ICD10CM
X73.8XXD	Intentional self-harm by other larger firearm discharge, subsequent encounter	ICD10CM
X73.8XXS	Intentional self-harm by other larger firearm discharge, sequela	ICD10CM
X73.9	Intentional self-harm by unspecified larger firearm discharge	ICD10CM
X73.9XXA	Intentional self-harm by unspecified larger firearm discharge, initial encounter	ICD10CM
X73.9XXD	Intentional self-harm by unspecified larger firearm discharge, subsequent encounter	ICD10CM
X73.9XXS	Intentional self-harm by unspecified larger firearm discharge, sequela	ICD10CM
X74	Intentional self-harm by other and unspecified firearm and gun discharge	ICD10CM
X74.0	Intentional self-harm by gas, air or spring-operated guns	ICD10CM
X74.01	Intentional self-harm by airgun	ICD10CM
X74.01XA	Intentional self-harm by airgun, initial encounter	ICD10CM
X74.01XD	Intentional self-harm by airgun, subsequent encounter	ICD10CM
X74.01XS	Intentional self-harm by airgun, sequela	ICD10CM
X74.02	Intentional self-harm by paintball gun	ICD10CM
X74.02XA	Intentional self-harm by paintball gun, initial encounter	ICD10CM
X74.02XD	Intentional self-harm by paintball gun, subsequent encounter	ICD10CM
X74.02XS	Intentional self-harm by paintball gun, sequela	ICD10CM
X74.09	Intentional self-harm by other gas, air or spring-operated gun	ICD10CM
X74.09XA	Intentional self-harm by other gas, air or spring-operated gun, initial encounter	ICD10CM
X74.09XD	Intentional self-harm by other gas, air or spring-operated gun, subsequent encounter	ICD10CM
X74.09XS	Intentional self-harm by other gas, air or spring-operated gun, sequela	ICD10CM
X74.8	Intentional self-harm by other firearm discharge	ICD10CM
X74.8XXA	Intentional self-harm by other firearm discharge, initial encounter	ICD10CM
X74.8XXD	Intentional self-harm by other firearm discharge, subsequent encounter	ICD10CM
X74.8XXS	Intentional self-harm by other firearm discharge, sequela	ICD10CM
X74.9	Intentional self-harm by unspecified firearm discharge	ICD10CM
X74.9XXA	Intentional self-harm by unspecified firearm discharge, initial encounter	ICD10CM
X74.9XXD	Intentional self-harm by unspecified firearm discharge, subsequent encounter	ICD10CM
X74.9XXS	Intentional self-harm by unspecified firearm discharge, sequela	ICD10CM
X75	Intentional self-harm by explosive material	ICD10CM
X75.XXXA	Intentional self-harm by explosive material, initial encounter	ICD10CM
X75.XXXD	Intentional self-harm by explosive material, subsequent encounter	ICD10CM
X75.XXXS	Intentional self-harm by explosive material, sequela	ICD10CM
X76	Intentional self-harm by smoke, fire and flames	ICD10CM
X76.XXXA	Intentional self-harm by smoke, fire and flames, initial encounter	ICD10CM
X76.XXXD	Intentional self-harm by smoke, fire and flames, subsequent encounter	ICD10CM
X76.XXXS	Intentional self-harm by smoke, fire and flames, sequela	ICD10CM
X77	Intentional self-harm by steam, hot vapors and hot objects	ICD10CM
X77.0	Intentional self-harm by steam or hot vapors	ICD10CM

© 2026 Lee H et al. *JAMA Network Open.*

X77.0XXA	Intentional self-harm by steam or hot vapors, initial encounter	ICD10CM
X77.0XXD	Intentional self-harm by steam or hot vapors, subsequent encounter	ICD10CM
X77.0XXS	Intentional self-harm by steam or hot vapors, sequela	ICD10CM
X77.1	Intentional self-harm by hot tap water	ICD10CM
X77.1XXA	Intentional self-harm by hot tap water, initial encounter	ICD10CM
X77.1XXD	Intentional self-harm by hot tap water, subsequent encounter	ICD10CM
X77.1XXS	Intentional self-harm by hot tap water, sequela	ICD10CM
X77.2	Intentional self-harm by other hot fluids	ICD10CM
X77.2XXA	Intentional self-harm by other hot fluids, initial encounter	ICD10CM
X77.2XXD	Intentional self-harm by other hot fluids, subsequent encounter	ICD10CM
X77.2XXS	Intentional self-harm by other hot fluids, sequela	ICD10CM
X77.3	Intentional self-harm by hot household appliances	ICD10CM
X77.3XXA	Intentional self-harm by hot household appliances, initial encounter	ICD10CM
X77.3XXD	Intentional self-harm by hot household appliances, subsequent encounter	ICD10CM
X77.3XXS	Intentional self-harm by hot household appliances, sequela	ICD10CM
X77.8	Intentional self-harm by other hot objects	ICD10CM
X77.8XXA	Intentional self-harm by other hot objects, initial encounter	ICD10CM
X77.8XXD	Intentional self-harm by other hot objects, subsequent encounter	ICD10CM
X77.8XXS	Intentional self-harm by other hot objects, sequela	ICD10CM
X77.9	Intentional self-harm by unspecified hot objects	ICD10CM
X77.9XXA	Intentional self-harm by unspecified hot objects, initial encounter	ICD10CM
X77.9XXD	Intentional self-harm by unspecified hot objects, subsequent encounter	ICD10CM
X77.9XXS	Intentional self-harm by unspecified hot objects, sequela	ICD10CM
X78	Intentional self-harm by sharp object	ICD10CM
X78.0	Intentional self-harm by sharp glass	ICD10CM
X78.0XXA	Intentional self-harm by sharp glass, initial encounter	ICD10CM
X78.0XXD	Intentional self-harm by sharp glass, subsequent encounter	ICD10CM
X78.0XXS	Intentional self-harm by sharp glass, sequela	ICD10CM
X78.1	Intentional self-harm by knife	ICD10CM
X78.1XXA	Intentional self-harm by knife, initial encounter	ICD10CM
X78.1XXD	Intentional self-harm by knife, subsequent encounter	ICD10CM
X78.1XXS	Intentional self-harm by knife, sequela	ICD10CM
X78.2	Intentional self-harm by sword or dagger	ICD10CM
X78.2XXA	Intentional self-harm by sword or dagger, initial encounter	ICD10CM
X78.2XXD	Intentional self-harm by sword or dagger, subsequent encounter	ICD10CM
X78.2XXS	Intentional self-harm by sword or dagger, sequela	ICD10CM
X78.8	Intentional self-harm by other sharp object	ICD10CM
X78.8XXA	Intentional self-harm by other sharp object, initial encounter	ICD10CM
X78.8XXD	Intentional self-harm by other sharp object, subsequent encounter	ICD10CM
X78.8XXS	Intentional self-harm by other sharp object, sequela	ICD10CM
X78.9	Intentional self-harm by unspecified sharp object	ICD10CM
X78.9XXA	Intentional self-harm by unspecified sharp object, initial encounter	ICD10CM

© 2026 Lee H et al. *JAMA Network Open.*

X78.9XXD	Intentional self-harm by unspecified sharp object, subsequent encounter	ICD10CM
X78.9XXS	Intentional self-harm by unspecified sharp object, sequela	ICD10CM
X79	Intentional self-harm by blunt object	ICD10CM
X79.XXXA	Intentional self-harm by blunt object, initial encounter	ICD10CM
X79.XXXD	Intentional self-harm by blunt object, subsequent encounter	ICD10CM
X79.XXXS	Intentional self-harm by blunt object, sequela	ICD10CM
X80	Intentional self-harm by jumping from a high place	ICD10CM
X80.XXXA	Intentional self-harm by jumping from a high place, initial encounter	ICD10CM
X80.XXXD	Intentional self-harm by jumping from a high place, subsequent encounter	ICD10CM
X80.XXXS	Intentional self-harm by jumping from a high place, sequela	ICD10CM
X81	Intentional self-harm by jumping or lying in front of moving object	ICD10CM
X81.0	Intentional self-harm by jumping or lying in front of motor vehicle	ICD10CM
X81.0XXA	Intentional self-harm by jumping or lying in front of motor vehicle, initial encounter	ICD10CM
X81.0XXD	Intentional self-harm by jumping or lying in front of motor vehicle, subsequent encounter	ICD10CM
X81.0XXS	Intentional self-harm by jumping or lying in front of motor vehicle, sequela	ICD10CM
X81.1	Intentional self-harm by jumping or lying in front of (subway) train	ICD10CM
X81.1XXA	Intentional self-harm by jumping or lying in front of (subway) train, initial encounter	ICD10CM
X81.1XXD	Intentional self-harm by jumping or lying in front of (subway) train, subsequent encounter	ICD10CM
X81.1XXS	Intentional self-harm by jumping or lying in front of (subway) train, sequela	ICD10CM
X81.8	Intentional self-harm by jumping or lying in front of other moving object	ICD10CM
X81.8XXA	Intentional self-harm by jumping or lying in front of other moving object, initial encounter	ICD10CM
X81.8XXD	Intentional self-harm by jumping or lying in front of other moving object, subsequent encounter	ICD10CM
X81.8XXS	Intentional self-harm by jumping or lying in front of other moving object, sequela	ICD10CM
X82	Intentional self-harm by crashing of motor vehicle	ICD10CM
X82.0	Intentional collision of motor vehicle with other motor vehicle	ICD10CM
X82.0XXA	Intentional collision of motor vehicle with other motor vehicle, initial encounter	ICD10CM
X82.0XXD	Intentional collision of motor vehicle with other motor vehicle, subsequent encounter	ICD10CM
X82.0XXS	Intentional collision of motor vehicle with other motor vehicle, sequela	ICD10CM
X82.1	Intentional collision of motor vehicle with train	ICD10CM
X82.1XXA	Intentional collision of motor vehicle with train, initial encounter	ICD10CM
X82.1XXD	Intentional collision of motor vehicle with train, subsequent encounter	ICD10CM
X82.1XXS	Intentional collision of motor vehicle with train, sequela	ICD10CM
X82.2	Intentional collision of motor vehicle with tree	ICD10CM
X82.2XXA	Intentional collision of motor vehicle with tree, initial encounter	ICD10CM
X82.2XXD	Intentional collision of motor vehicle with tree, subsequent encounter	ICD10CM
X82.2XXS	Intentional collision of motor vehicle with tree, sequela	ICD10CM
X82.8	Other intentional self-harm by crashing of motor vehicle	ICD10CM
X82.8XXA	Other intentional self-harm by crashing of motor vehicle, initial encounter	ICD10CM
X82.8XXD	Other intentional self-harm by crashing of motor vehicle, subsequent encounter	ICD10CM
X82.8XXS	Other intentional self-harm by crashing of motor vehicle, sequela	ICD10CM
X83	Intentional self-harm by other specified means	ICD10CM
X83.0	Intentional self-harm by crashing of aircraft	ICD10CM

© 2026 Lee H et al. *JAMA Network Open.*

X83.0XXA	Intentional self-harm by crashing of aircraft, initial encounter	ICD10CM
X83.0XXD	Intentional self-harm by crashing of aircraft, subsequent encounter	ICD10CM
X83.0XXS	Intentional self-harm by crashing of aircraft, sequela	ICD10CM
X83.1	Intentional self-harm by electrocution	ICD10CM
X83.1XXA	Intentional self-harm by electrocution, initial encounter	ICD10CM
X83.1XXD	Intentional self-harm by electrocution, subsequent encounter	ICD10CM
X83.1XXS	Intentional self-harm by electrocution, sequela	ICD10CM
X83.2	Intentional self-harm by exposure to extremes of cold	ICD10CM
X83.2XXA	Intentional self-harm by exposure to extremes of cold, initial encounter	ICD10CM
X83.2XXD	Intentional self-harm by exposure to extremes of cold, subsequent encounter	ICD10CM
X83.2XXS	Intentional self-harm by exposure to extremes of cold, sequela	ICD10CM
X83.8	Intentional self-harm by other specified means	ICD10CM
X83.8XXA	Intentional self-harm by other specified means, initial encounter	ICD10CM
X83.8XXD	Intentional self-harm by other specified means, subsequent encounter	ICD10CM
X83.8XXS	Intentional self-harm by other specified means, sequela	ICD10CM

© 2026 Lee H et al. *JAMA Network Open.*

eTable 2. AUROCs and AUPRCs (IQR) and Their Pairwise Statistical Comparison Between Data Modalities Using Bootstrap CIs

**Data Modality**	**VUH**	**RHS**
**AUROC**		
**VSAIL**	0.645 [0.645 – 0.645]	0.547 [0.547 – 0.547]
**Psychosocial factors**	0.726 [0.713 – 0.740]	0.685 [0.677 – 0.692]
**VSAIL + Psychosocial factors**	0.734 [0.719 – 0.747]	0.680 [0.672 – 0.687]
**AUPRC**		
**VSAIL**	0.083 [0.083 – 0.083]	0.029 [0.029 – 0.029]
**Psychosocial factors**	0.117 [0.106 – 0.130]	0.057 [0.054 – 0.059]
**VSAIL + Psychosocial factors**	0.122 [0.111 – 0.137]	0.054 [0.052 – 0.058]
**VUH**		
**AUROC**		
	**Psychosocial factors**	**VSAIL + Psychosocial factors**
**VSAIL**	0.080 – 0.086 (*P*<.001)	0.087 – 0.093 (*P*<.001)
**Psychosocial factors**	-	0.006 – 0.007 (*P*<.001)
**AUPRC**		
	**Psychosocial factors**	**VSAIL + Psychosocial factors**
**VSAIL**	0.033 – 0.037 (*P*<.001)	0.039 – 0.043 (*P*<.001)
**Psychosocial factors**	-	0.006 – 0.007 (*P*<.001)
**PPV**		
	**Psychosocial factors**	**VSAIL + Psychosocial factors**
**VSAIL**	0.048 – 0.054 (*P*<.001)	0.050 – 0.055 (*P*<.001)
**Psychosocial factors**	-	0.0002 – 0.003 (*P*=.02)
**Specificity**		
	**Psychosocial factors**	**VSAIL + Psychosocial factors**
**VSAIL**	-0.009 – -0.007 (*P*<.001)	-0.008 – -0.006 (*P*<.001)
**Psychosocial factors**	-	0.0004 – 0.001 (*P*=.01)
**RHS**		
**AUROC**		
	**Psychosocial factors**	**VSAIL + Psychosocial factors**
**VSAIL**	0.137 – 0.139 (*P*<.001)	0.131 – 0.134 (*P*<.001)
**Psychosocial factors**	-	-0.0055 – -0.0046 (*P*<.001)
**AUPRC**		
	**Psychosocial factors**	**VSAIL + Psychosocial factors**
**VSAIL**	0.027 – 0.028 (*P*<.001)	0.025 – 0.026 (*P*<.001)
**Psychosocial factors**	-	-0.002 – -0.0017 (*P*<.001)
**PPV**		
	**Psychosocial factors**	**VSAIL + Psychosocial factors**
**VSAIL**	0.068 – 0.072 (*P*<.001)	0.069 – 0.074 (*P*<.001)
**Psychosocial factors**	-	0.001 – 0.003 (*P*=.01)
**Specificity**		
	**Psychosocial factors**	**VSAIL + Psychosocial factors**
**VSAIL**	0.031 – 0.033 (*P*<.001)	0.032 – 0.034 (*P*<.001)
**Psychosocial factors**	-	0.0002 – 0.001 (*P*=.006)

Bootstrap confidence intervals and Wilcoxon signed-rank test were used to test the difference in AUROCs and AUPRCs between data modalities.

© 2026 Lee H et al. *JAMA Network Open.*

eTable 3. Standardized β Coefficients and 95% CIs for Predictors of 90-Day Suicide Attempt From a Cox Proportional Hazards Regression Model

**Predictor**	**β Coefficient (95% CI)**	***P* value**
Chronic Stress	0.643(0.427 to 0.859)	<.001
Adverse Childhood Experiences	0.240 (0.106 to 0.374)	<.001
VSAIL score	0.162 (0.005 to 0.320)	.04
Homelessness	0.151 (-0.026 to 0.329)	.10
Financial Insecurity	0.124 (-0.023 to 0.271)	.10
Loneliness	0.083 (-0.045 to 0.211)	.20
Social Isolation	0.078 (-0.074 to 0.231)	.31

All predictors were standardized to a mean of 0 and a standard deviation of 1, enabling direct comparison of β coefficients. The Vanderbilt Suicide Attempt and Ideation Likelihood (VSAIL) score represents a composite suicide attempt risk estimate, generated by a random forest model trained on structured EHR data at VUMC. The six psychosocial factors were derived as log-transformed cosine similarity metrics (relevance scores), extracted from clinical notes using a vector embedding-based natural language processing algorithm developed, validated, and implemented at VUMC.
