# Supplementary material for: Clinical Note–Extracted Psychosocial Factors for Predicting Suicide Attempt Among ED Patients With Suicidal Ideation
Source: JAMA Netw Open. 2026 Mar 4;9(3):e260589. doi: 10.1001/jamanetworkopen.2026.0589 (PMC12961534; doi:10.1001/jamanetworkopen.2026.0589)
Supplement: Supplement 2. — Data Sharing Statement [file jamanetwopen-e260589-s002.pdf]

Data Sharing Statement

Lee. Clinical Note–Extracted Psychosocial Factors for Predicting Suicide Attempt Among ED Patients With Suicidal Ideation. *JAMA Netw Open*. Published March 04, 2026. doi:10.1001/jamanetworkopen.2026.0589

Data
Data available: No

Additional Information
Explanation for why data not available: The data used in this study cannot be made publicly available due to Vanderbilt University Medical Center restrictions relating to the use of electronic health records data.
